# Framework for the development and evaluation of complex interventions: gap analysis, workshop and consultation-informed update

**DOI:** 10.3310/hta25570

**Published:** 2021-09-01

**Authors:** Kathryn Skivington, Lynsay Matthews, Sharon Anne Simpson, Peter Craig, Janis Baird, Jane M Blazeby, Kathleen Anne Boyd, Neil Craig, David P French, Emma McIntosh, Mark Petticrew, Jo Rycroft-Malone, Martin White, Laurence Moore

**Affiliations:** 1 Medical Research Council/Chief Scientist Office Social and Public Health Sciences Unit, Institute of Health and Wellbeing, University of Glasgow, Glasgow, UK; 2 Medical Research Council Lifecourse Epidemiology Unit, University of Southampton, Southampton, UK; 3 Medical Research Council ConDuCT-II Hub for Trials Methodology Research and Bristol Biomedical Research Centre, University of Bristol, Bristol, UK; 4 Health Economics and Health Technology Assessment Unit, Institute of Health and Wellbeing, University of Glasgow, Glasgow, UK; 5 Public Health Scotland, Glasgow, UK; 6 Manchester Centre for Health Psychology, University of Manchester, Manchester, UK; 7 London School of Hygiene and Tropical Medicine, London, UK; 8 Faculty of Health and Medicine, Lancaster University, Lancaster, UK; 9 Medical Research Council Epidemiology Unit, University of Cambridge, Cambridge, UK

## Abstract

**Background:**

The Medical Research Council published the second edition of its framework in 2006 on developing and evaluating complex interventions. Since then, there have been considerable developments in the field of complex intervention research. The objective of this project was to update the framework in the light of these developments. The framework aims to help research teams prioritise research questions and design, and conduct research with an appropriate choice of methods, rather than to provide detailed guidance on the use of specific methods.

**Methods:**

There were four stages to the update: (1) gap analysis to identify developments in the methods and practice since the previous framework was published; (2) an expert workshop of 36 participants to discuss the topics identified in the gap analysis; (3) an open consultation process to seek comments on a first draft of the new framework; and (4) findings from the previous stages were used to redraft the framework, and final expert review was obtained. The process was overseen by a Scientific Advisory Group representing the range of relevant National Institute for Health Research and Medical Research Council research investments.

**Results:**

Key changes to the previous framework include (1) an updated definition of complex interventions, highlighting the dynamic relationship between the intervention and its context; (2) an emphasis on the use of diverse research perspectives: efficacy, effectiveness, theory-based and systems perspectives; (3) a focus on the usefulness of evidence as the basis for determining research perspective and questions; (4) an increased focus on interventions developed outside research teams, for example changes in policy or health services delivery; and (5) the identification of six ‘core elements’ that should guide all phases of complex intervention research: consider context; develop, refine and test programme theory; engage stakeholders; identify key uncertainties; refine the intervention; and economic considerations. We divide the research process into four phases: development, feasibility, evaluation and implementation. For each phase we provide a concise summary of recent developments, key points to address and signposts to further reading. We also present case studies to illustrate the points being made throughout.

**Limitations:**

The framework aims to help research teams prioritise research questions and design and conduct research with an appropriate choice of methods, rather than to provide detailed guidance on the use of specific methods. In many of the areas of innovation that we highlight, such as the use of systems approaches, there are still only a few practical examples. We refer to more specific and detailed guidance where available and note where promising approaches require further development.

**Conclusions:**

This new framework incorporates developments in complex intervention research published since the previous edition was written in 2006. As well as taking account of established practice and recent refinements, we draw attention to new approaches and place greater emphasis on economic considerations in complex intervention research. We have introduced a new emphasis on the importance of context and the value of understanding interventions as ‘events in systems’ that produce effects through interactions with features of the contexts in which they are implemented. The framework adopts a pluralist approach, encouraging researchers and research funders to adopt diverse research perspectives and to select research questions and methods pragmatically, with the aim of providing evidence that is useful to decision-makers.

**Future work:**

We call for further work to develop relevant methods and provide examples in practice. The use of this framework should be monitored and the move should be made to a more fluid resource in the future, for example a web-based format that can be frequently updated to incorporate new material and links to emerging resources.

**Funding:**

This project was jointly funded by the Medical Research Council (MRC) and the National Institute for Health Research (Department of Health and Social Care 73514).

## Introduction

In 2006, the Medical Research Council (MRC) published guidance for developing and evaluating complex interventions,^1^ building on a framework that was published in 2000.^2^ The aim was to help researchers and research funders recognise and adopt appropriate methods to improve the quality of research to develop and evaluate complex interventions and, thereby, maximise its impact. The guidance documents have been highly influential, and the accompanying papers published in the *British Medical Journal* are widely cited.^3,4^

Since the 2006 edition of the guidance was published, there have been considerable developments in the field of complex intervention research. In some areas, the accumulation of experience and knowledge in the application of approaches and methods that were novel or undeveloped at the time of writing the previous guidance has led to the publication of detailed, focused guidance on the conduct and/or reporting of research, including the MRC guidance on ‘process evaluation’.^5^ In other areas, new challenges in complex intervention research have been identified and the reliance on traditional approaches and methods promoted in previous guidance has been challenged.^6–8^ The guidance has also been complemented by MRC guidance on ‘natural experiments’,^9^ an important area of development in methods and practice. Given that complex intervention research is a broader and more active field, this new framework provides a less prescriptive and more flexible guide. The framework aims to improve the design and conduct of complex intervention research to increase its utility, efficiency and impact. Consistent with the principles of increasing the value of research and minimising research waste, the framework (1) emphasises the use of diverse research perspectives and the inclusion of research users, clinicians, patients and the public in research teams; and (2) aims to help research teams prioritise research questions and choose and implement appropriate methods. This aims to provide pragmatic recommendations aimed at an audience from multiple disciplines, and we have taken a pluralist approach.

Updating the framework was a pragmatic, staged process, in which the findings from one stage fed into the next. The next section, therefore, provides the methods followed by the results for each stage (gap analysis, expert workshop, open consultation and writing the new framework). We then provide concluding remarks and suggestions for moving forward. The resulting framework is presented in Chapter 2.

This project was overseen by a Scientific Advisory Group (SAG) that comprised representatives of each of the National Institute for Health Research (NIHR) programmes, the MRC–NIHR Methodology Research Panel, key MRC population health research investments and authors of the 2006 guidance (see Appendix 1). A prospectively agreed protocol, outlining the work plan, was agreed with the MRC and NIHR and signed off by the SAG (see Appendix 2). At various points throughout the writing process, we consulted with other researchers, evidence users, journal editors and funders (see Appendix 3).

As terminology can often be ambiguous, and there are often terms used interchangeably, we have provided a *Glossary* of key terms.

## Methods and results

The framework was updated using multiple methods over several stages:

- stage 1 – a gap analysis of the evidence base for complex interventions
- stage 2 – a workshop that collated insight from current experts in the field
- stage 3 – an open consultation
- stage 4 – drafting the updated framework and a final feedback exercise.

Various stakeholders, for example researchers, research users (patients, public, policy-influencers and NHS), funders and journal editors, were engaged at different stages of the drafting process. The methods and findings from each of the stages are described in the following sections.

### Stage 1: gap analysis

#### Methods for stage 1: gap analysis

The aim of the gap analysis was to identify and summarise aspects of the previous guidance that required updating. We used these gaps as a starting point for discussion within the project team, SAG (for a list of members, see Appendix 3) and identified experts. It was, therefore, a method of agenda setting and, thus, did not aim to be comprehensive. The intention was that issues could be added as the work progressed.

Our first step was a brief horizon scanning review that focused on new approaches/progress since the previous guidance, criticisms of existing guidance and other gaps. Based on initial reading of the literature and the experience of the project team, the SAG were provided with a list of topics for update. This was discussed at the initial SAG meeting (24 November 2017) and the list of topics was updated for more in-depth exploration of the literature.

Separate literature searches were conducted for each of the identified topics using keywords (the topic of interest plus variations of ‘complex intervention’) in Web of Science, restricted to English language with the date limited to those published since 2008. Where there were existing guidance documents relevant to the development, implementation or evaluation of complex interventions, we used these as our starting point and limited our literature review to documents published after these existing guidance documents. For example, guidance existed for natural experiments,^9^ process evaluation^5^ and context,^10^ which we drew heavily on. We also discussed this new framework with those involved in developing other guidance at the time, for example for intervention development,^11^ exploratory studies^12^ and systems thinking.^13,14^ We excluded guidance that did not provide substantive information on methodological issues. Criteria for including other publications were broadly that they provided relevant thinking that could be used to progress the work. A summary of the findings from each topic search was created and used to identify focal points for the expert workshop.

#### Findings from stage 1: gap analysis

Several limitations of the 2006 guidance were highlighted. These mainly related to (1) the focus on effectiveness; (2) considering randomised controlled trials (RCTs) as the gold standard research design; (3) the lack of detail on economic evaluation; (4) the lack of attention to mechanisms that deliver change; (5) the lack of acknowledgement of complex organisational systems or complexity theory; and (6) omission of the importance of policy context, including the impossibility of standardising context. In addition to these issues, there were several areas that had progressed since 2006, which were considered to be important to explore in more detail. Here we provide a brief summary of the gaps that were identified in 2017 and what we initially proposed to be discussed with experts at the workshop:

Complex intervention definition –
  ∘ Key issue for the update – definition is too narrow.
  ∘ There are different dimensions of complexity. How can we improve the definition of complex intervention to better acknowledge contextual complexity and the system-level properties that add to this complexity?
  ∘ Does the term ‘complex intervention’ make sense when complexity arises from the intervention context and the interplay between intervention and context as well as the intervention itself?
Intervention development –
  ∘ Key issue for update – little practical guidance on this phase and subsequent literature provides more detail on certain aspects, for example using a theory-driven approach;^15^,^16^ identifying and delivering the mechanism of change;^17^ co-production and prototyping of public health interventions;^18^ and optimisation of the intervention.^19^
  ∘ There is guidance under way on intervention development (INDEX study^11^) that is about identifying and assessing different approaches to developing complex interventions. Given that this is current and there are clear overlaps, are there any issues that are not covered in the INDEX guidelines that we should consider adding in this document?
Pre-evaluation phase: appraisal of evaluation options or exploratory work –
  ∘ Key issue for update – previous complex intervention guidance highlighted the importance of preparatory work, with the focus being on conducting a pilot study; however, detail on feasibility issues and how to develop the feasibility stage is required.
  ∘ Similar to the development phase, there is work in progress to create guidance for Exploratory Studies of Complex Public Health Interventions (GuESt study^12^), which includes a lot of relevant information. Should the current update include anything further and how can we make it relevant beyond public health?
Context –
  ∘ Key issue to update – although the previous complex intervention guidance states the importance of taking account of context, this is mostly about how context affects outcomes at the evaluation phase and how interventions may require adaptation for implementation in different contexts. There is little attention to the consideration of context throughout the research phases or guidance on how to take it into account.
  ∘ How do ‘context’ and ‘system’ differ/overlap?
  ∘ Context is a critical construct; how do we ensure that we refer to it throughout the research process?
  ∘ Do we want to go further than the recently published guidance on taking account of context in population health intervention research?^10^ What are the key points for considering context in complex intervention research more broadly?
Ideas from complex systems science –
  ∘ Key issue to update – this is an area that has received increasing attention in the last decade, and for this reason the previous complex intervention guidance did not draw on it.
  ∘ Examples of using complex systems thinking in public health research have been limited to describing and modelling systems; this has not yet been taken further and been used to develop and evaluate interventions.^7^
  ∘ When is it critical to embrace a complexity perspective (and when it is not necessary: simple and complicated questions and approaches have their merits) and how can such a perspective be implemented methodologically?
  ∘ How can a complex systems approach guide each phase of complex intervention research?
Programme theory –
  ∘ Key issue to update – the previous complex intervention guidance provided brief information on causal mechanisms and on developing a theoretical understanding of the process of change; however, this lacks the required level of information to guide researchers in developing programme theory from the outset.
  ∘ Further detail is needed to illustrate the steps required to undertake a robust planning phase, including (1) identifying appropriate theories of change, (2) considering potential mechanisms of change, (3) anticipating important contextual factors that could influence the change mechanism and outcomes and (4) mapping appropriate methods to operationalise the chosen theory into practice.
Implementation research –
  ∘ Key issue to update – the previous complex intervention guidance has limited information on the practical implementation process and needs to understand and account for dynamic contextual factors.
  ∘ Successful implementation is critical to the scaling up of interventions and the new framework should reflect this by emphasising implementation throughout the research process.
  ∘ When do you stop doing effectiveness studies and start doing implementation studies?
  ∘ How can we include the wider aspects of implementation that may enable or constrain desired change? For example, how much guidance do we provide on addressing intervention context and addressing future implementation on a greater scale?
  ∘ How do we make the information palatable for decision-makers?
Economic evaluation and priority setting –
  ∘ Key issue to update – the previous complex intervention guidance did not go into any detail on how standard economic evaluation methods need to be adapted to deal with particularly complex interventions.
  ∘ Issues around timeline – outcomes are likely to extend beyond the lifetime of an evaluation – can economists work with proxies to system change?
  ∘ How do we best guide on issues for existing economic evaluation methods where interventions aim to change the properties of complex systems? That is, it is not appropriate to evaluate health outcomes only at the individual level if a component of the intervention is to effect change to the system; outcomes are broader than individual health and costs (is a societal rather than a health-care perspective required?).
  ∘ (How) should we include equity issues and economic evaluation analytical approaches, which are growing and complicated methodological areas?
  ∘ How can we guide on economic evaluation for priority setting? That is, what is the most efficient use of resources (to determine whether or not the additional cost of a research project or particular study design is justified)? Are decision-modelling and value-of-information analysis (VOI) practical propositions?
Systematic reviews of complex interventions –
  ∘ Key issue to update – the previous complex intervention guidance did not address issues related to the inclusion of complex intervention studies in systematic reviews, much beyond acknowledging that they can be problematic. Should we add more?
  ∘ Systematic review methods may differ from standard methods and extra consideration is necessary where the systematic review includes complex interventions (if the review is about complexity), for example in defining the research questions, developing the protocol, the use of theory, searching for relevant evidence, and assessing complexity and quality of evidence (how to identify key components of complex interventions; how to assess study quality).
  ∘ What should be the end point of a systemic review of complex interventions? For example, effect size, decision model, improved theory or supporting policy decisions?
Patient and public involvement (PPI) and co-production –
  ∘ Key issue to update – previous complex intervention guidance mentioned that stakeholders should be consulted at various points, but did not emphasise the need to engage relevant stakeholders throughout the research process or provide any guidance on how to do this.
  ∘ How do we guide on effective engagement of stakeholders throughout?
Evaluation –
  ∘ Key issue to update – the previous complex intervention guidance focused on designing evaluations to minimise bias (i.e. with high internal validity) and, in doing so, did not consider how to maximise the usefulness of evidence for decision-making. These are not mutually exclusive concerns: could both be considered?
  ∘ Should we take an approach that promotes ‘usefulness of evidence’ rather than hierarchy of evaluation study design?
  ∘ Should we present evaluation options that go beyond individual-level primary health outcomes? For example, taking account of system change.
  ∘ Evaluation study designs – what should be added to reflect development in this area? For example, *n*-of-1, adaptive trials. How much information should we present on individual study design?

These topic areas and questions were intended to be a foundation for discussion and further consideration, rather than an exhaustive or definitive list.

### Stage 2: expert workshop

#### Methods for stage 2: expert workshop

A 1-day expert workshop was convened in London in May 2018. A list of those who attended the workshop is given in Appendix 3. The aim of the workshop was to obtain views and record discussions on topics that should be newly covered or updated. Participants were identified by the project team and SAG. We aimed to have at least two experts for each of the identified topics and include a range of people from across the UK, plus international representation as far as budget allowed.

In advance of the workshop, the participants were asked to provide two key points, each with one sentence of explanation, that they felt should be taken into account in the update. These key points, alongside findings from the stage 1 gap analysis and discussion with the SAG, were used to inform the agenda and develop content for an interactive, multidisciplinary expert workshop.

After an introductory presentation by the project team, participants were split into five groups (of seven or eight) for the morning session round-table discussion.

The topics covered for all groups (presented in a different order) were:

- the definition of complexity
- the overall framing and scope
- potential impact of the new framework
- the main diagram of the framework (key elements of the development and evaluation process)
- complex systems thinking.

For each of the two afternoon sessions, participants were split into five ‘expert groups’ aligned with their topic areas of expertise. Topics covered in these smaller specialised groups included:

- options for study design
- the previous guidance’s emphasis on ‘effectiveness’
- choice of outcomes
- considerations for economic evaluation
- pre-evaluation and development phases
- considerations for implementation
- key focus areas to improve applications for funding
- evidence synthesis of complex interventions
- considerations for digital health
- programme theory.

Each session was facilitated by a member of the project team and was supported by a colleague from the MRC/Chief Scientist Office Social and Public Health Sciences Unit, University of Glasgow. Colleagues assisted the facilitators by taking notes of key points during each discussion, clarifying main points with attendees and producing a written summary of each discussion after the workshop. SAG members were also present in each discussion. Round-table discussions were audio-recorded. Throughout the day, participants were asked to provide their thoughts on key points, case study examples and key references on Post-it® notes (3M, Saint Paul, MN, USA) on dedicated noticeboards.

Data from each of the 15 workshop discussions and post-it points were thematically coded, and summaries drawing on all of the data were created for each theme. These workshop summaries were sent to workshop participants by e-mail as a follow-up consultation to ensure that the thematic summaries that we created from the workshops were accurate overviews of the discussions in which they were involved. Final summaries were discussed in detail with the SAG to support the decision-making on the content of the document.

#### Findings from stage 2: expert workshop

Seventy experts were invited to the workshop (with the aim of facilitating a workshop of around 40 participants). In total, 37 experts confirmed their attendance; one who accepted the invitation did not attend (owing to sickness), three people did not respond to our invitation and 30 people could not attend for various reasons, some of whom recommended others who did attend. In total, 36 participants attended the workshop. Key issues that were identified are summarised in Table 1.

##### Decisions taken following the expert workshop

There was considerable agreement across the workshop discussions; however, as seen in Table 1, there were some issues for which consensus was not reached or for which competing points were made in different break-out discussions. The main example of controversy was the purpose of evaluation (theory as an end point, the need for primary outcome). In addition, some of the points that were made were very specialised, for example related to particular methods or specialties. Along with the SAG, the project team determined which focus areas to incorporate in the document, keeping them high level rather than getting into specific detail. With respect to the issues for which views diverged (primarily related to effectiveness and the purpose of evaluation), we consider the document as a ‘thinking tool’ to provide recommendations to arrive at the most appropriate approach for each piece of complex intervention research (with no ‘one size fits all’ approach, instead determined by the problem that is being addressed and taking a ‘usefulness of evidence’ approach).

**Table 1:** Summary of the key points from the expert workshop in 2018

Topic	Key points
Definition of complex intervention	Requires clarity regarding the use of the terms simple and complex, and the use of the term intervention Needs to include context, systems thinking, feedback loops and the fundamental aspects of complexity Thinking about complex interventions in terms of how ‘simple’or ‘complicated’they are is not very helpful, as there are many aspects that make an intervention complex
Framing and scope	Reaching a new audience is important: as well as researchers, the framework needs to reach ‘practitioner researchers’, Health Technology Assessment and policy-makers. A common language is needed and it is, therefore, important to include different stakeholders in this process. Provide clarification of terminology A more iterative and fluid research process needs to be emphasised. More options should be given to avoid saying there’s ‘one way of doing this’
Intervention development	More emphasis on determining the problem and establishing the research questions Acknowledge that there are different starting points to the research process, for example the process does not necessarily start with development, researchers may not be involved in the intervention’s development (e.g. a national policy) and researchers could join/start at any phase
Study design	Agreement regarding the need to choose a design that is best suited to answer the research question in a given context (i.e. there is no novel design that caters for ‘complex interventions’) It is important to emphasise that there are more (and often more suitable) options than RCTs Guide people to think about the function of the intervention in choosing the study design. Give examples of what researchers have undertaken in different contexts and include case studies Usefulness of evidence is a good approach, rather than the previous hierarchy of evidence approach. This requires thinking of the right research questions for the intended use of the research Acknowledge that rapidly changing fields require faster routes of evaluation before the overall context changes (e.g. in digital health)
Systems thinking	General agreement regarding including systems thinking and encouraging researchers to think with a ‘systems lens’ However, there was also agreement that this field is developing and there are limitations to how much guidance we can currently give Introduce three levels of systems thinking: (1) conceptualisation of the system (what does it currently look like), (2) what parts of the system can be influenced (and what are the boundaries) and (3) what is happening outside the boundary (providing richer context) In relation to this, context is often poorly articulated in funding applications and guidance is required
Implementation science	Clarity is required on ongoing modification at the implementation phase Clarity on terminology required (delivery of intervention/implementation) Implementation should be emphasised throughout (e.g. from development stages)
Programme theory	Clarity on terminology is required (e.g. people are typically unclear on what theory is; there do not seem to be consistent definitions of terms, such as ‘logic model’ and ‘programme theory’; and other terms, such as mechanism of action, etc., need to be defined) Include information on theory (e.g. thinking about what the problem is at the start and what should be changed; articulating what theory is and is not; considering how theory may be influenced by context; and encouraging adaptive and iterative theory development) Researchers need to be encouraged to articulate their theory in full, not solely in a visual model that will miss some of the important detail
Economic evaluation	There was general agreement that new methods were not needed, but that standard methods could be adapted to more effectively explore complex interventions Signpost recent developments since the previous guidance and link them to the existing guidance Emphasise the need to measure aspects of programme theory and resource use, rather than just effectiveness of outcomes Mention cost-consequence analysis to help highlight links between processes and outcomes Emphasise the need for a broader range of outcomes End point: it should be about revealing the resource cost/outcome trade-offs and causal connection, as much as it is about producing a ratio or a number. We need the trade-offs for each resource use
Effectiveness	There were diverse views on whether or not traditional effectiveness is an appropriate end point for an evaluation, for example some were keen to abolish effect sizes altogether, look at other things and answer different questions (e.g. what happened?). This would include having theory as an end point in itself. Others disagreed that theory should be an end point and felt strongly that evaluations have to answer the ‘does it work?’question; however, there was general agreement that this (does it work?) in itself is not enough In relation to this, there was debate about whether or not evaluations should have primary (health) outcomes, with some of the opinion that there needs to be a prespecified outcome for the intervention that is being tested, and without a primary outcome there is the risk of ‘cherry picking’ the most improved outcome to make the intervention appear effective. Others disagreed and felt that evaluation should explore the impact on multiple effects, including system change (not individual effect sizes), with the goal of theory development
Stakeholders	There was more emphasis required than in the previous guidance Place higher priority on co-production and non-researcher-led interventions Articulation of the problem needs to come from shared space; genuine co-creation of interventions from this starting point
Evidence synthesis	Acknowledge that methods still need to be developed Context: ∘ The value of an evidence synthesis does not lie only in obtaining the most unbiased estimate (in terms of effect size) or in meta-analysis; there needs to be a focus on exploring heterogeneity across contexts and identifying the mechanisms that drive variation ∘ There will never be a complete suite of studies of complex interventions in all of the relevant contexts, so there needs to be a way of extrapolating from what we have o Provide pointers to things that would help decision-makers know if they could use the evidence and make judgements about transferability, how their context differs and what might be the things that facilitate change ∘ Decision-makers could be encouraged to think about socially significant differences in context One challenge is that, with some exceptions, evidence synthesis organisations focus on trials. The new framework should seek to widen the range of evidence included in syntheses used for decision-making and ensure the inclusion of mixed-methods research. It should also clarify what we mean by ‘evidence’ and acknowledge that this includes theory as well as information about outcomes General agreement that an improvement in primary studies that follows from the recommendations will have a positive impact on evidence synthesis in time (there will be more appropriate studies to synthesise)

Some examples of the decisions taken are as follows:

- Clarity in terminology – include a comprehensive glossary.
- Include a series of case studies as an appendix, highlighting particular aspects of each phase and core elements of the research process.
- Highlight the distinctive methods of evidence generation, emphasising that the research can begin at any stage of the intervention and that there may be different approaches for researchers not involved in intervention development.
- Not to be prescriptive but rather provide options for approaching the research, which should be chosen by taking the problem as the starting point and working out what is most useful in terms of decision-making for policy and practice going forward.
- Update the diagram included in the 2006 guidance that showed ‘Key elements of the development and evaluation process’,^1^ particularly to include context.
- Include a greater focus on programme theory, but one that encourages its consideration and refining throughout.
- Systems thinking – not to provide detailed guidance on systems thinking and methods because this is beyond the scope; rather it will be a starting point for encouraging people to consider how a systems perspective could help develop and evaluate complex interventions, with methodological development to follow.
- Evidence synthesis – following the expert workshop, information that others were developing guidance in this area and discussion with the SAG we took the decision to focus on primary studies; therefore, we did not include a section in the main document on evidence synthesis. It is hoped that an improvement in primary studies, brought about by this new framework, would in time have a positive impact on evidence synthesis. We added an appendix to highlight some of the main considerations for evidence synthesis (see Appendix 5).

Further decisions were taken regarding the need to obtain further expertise in drafting the document. We approached three health economists for a follow-up meeting to discuss further issues related to economic considerations for complex intervention research; following this, they agreed to take on the responsibility of drafting sections that related to economic considerations and became co-authors. We also approached experts in systems thinking to discuss some of the emerging ideas on taking a systems perspective to complex intervention research. We convened a meeting in December 2018 in London with a group of researchers with such expertise (individually acknowledged in this monograph). Similarly, we convened a meeting in January 2019 with researchers who were creating guidelines on intervention development (individually acknowledged in this monograph) to discuss the overlap and the use of the INDEX guidance within the current document.

### Stage 3: open consultation

#### Methods for stage 3: open consultation

The first draft of the updated document was made available for open consultation from Friday 22 March to Friday 5 April 2019.

Potential respondents were targeted, as follows:

- those invited to the expert workshop
- other experts identified from the suggestions of workshop participants, with greater focus on international experts
- early and mid-career researchers (identified via e-mail groups)
- journal editors
- funders
- service users/public
- policy-influencers/-makers.

We e-mailed potential respondents with advance notice of the consultation dates and a link to register their interest in participating, and sent a further message when the consultation opened. Two reminder e-mails were also sent. As well as targeted promotion, we used social media to publicise the consultation and encouraged others to pass on the link.

Consultees were informed that they were responding about an early draft of the revised framework and that their involvement was an important part of the process for its final development. We asked them to relate topics in the draft to a project that they had recently worked on and to provide feedback on its usability.

The online consultation was guided by a questionnaire that was developed by the project team (the questions that all consultees were asked to complete are presented in Appendix 4). Responses were anonymous.

#### Findings from stage 3: open consultation

We received 52 individual responses, plus some follow-up e-mail comments. This amounted to 25,000 words of response. The majority of responses were from researchers, but some identified as funders (*n* = 3), journal editors (*n* = 7), NHS (*n* = 7), policy-influencers (n = 3) and service users (patient or public, *n* = 5). Most of the respondents said that their main field of expertise was public health (*n* = 21) or health services research (*n* = 20), with others stating clinical medicine (*n* = 6), implementing policy (*n* = 3), systems-based research (*n* = 4), patient or public involvement (*n* = 4) and other (*n* = 7: statistics, sociology, health economics and triallist) as their main field of expertise. A summary of the consultation suggestions is provided below; however, it is important to note that there were conflicting views on some aspects, which we have noted.

##### Overall

**Figure 1:**
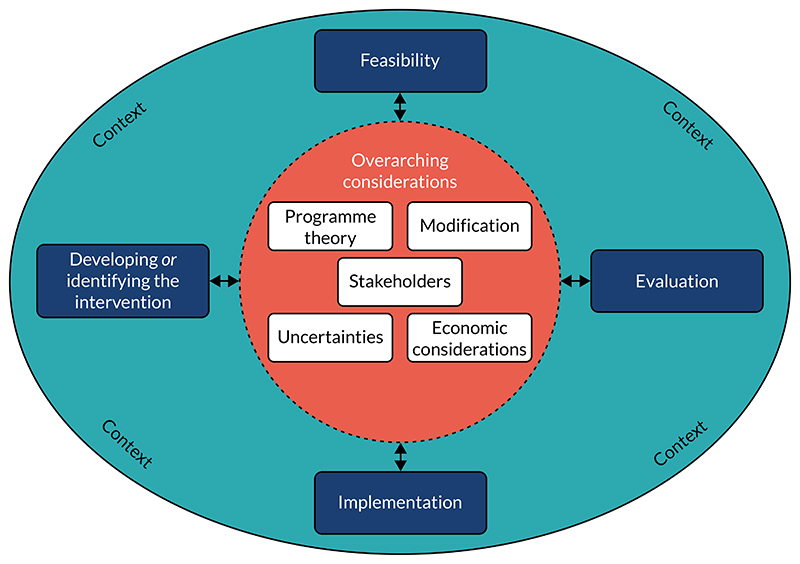
Key elements for developing and evaluating complex interventions (consultation version).

**Figure 2:**
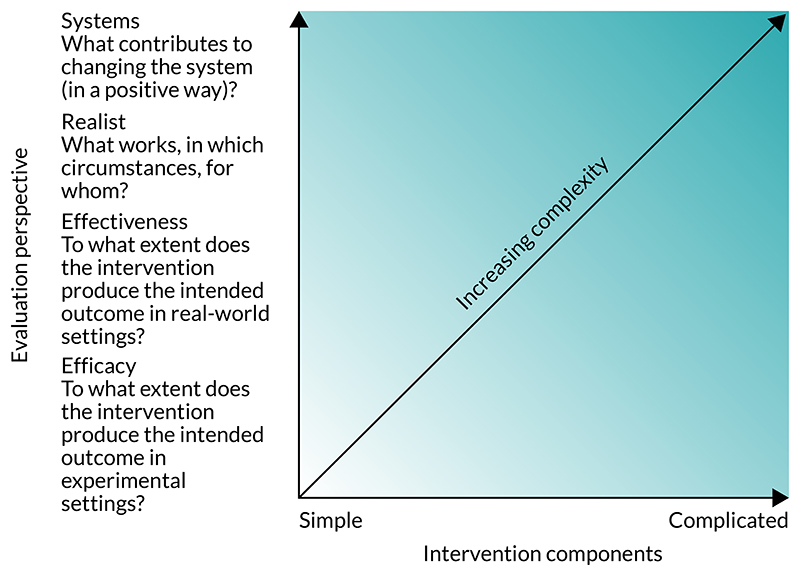
Framework for addressing complexity within evaluation (consultation version).

- Overall layout: extra sections are required – an executive summary and a preface chapter that details how this is related to previous guidance and that this document is a standalone framework that does not require reference back to the 2006 version. Consider placing more emphasis on development in the earlier sections of the document rather than delve straight into evaluation.
- Definition of complexity: the distinction made between complicated and complex interventions was said to be unclear. Respondents stressed that a clear definition of complex intervention and a more accessible account of how complexity affects the research process are required.
- Key elements for developing and evaluating complex interventions (Figure 1): respondents felt that the ‘overarching considerations’ should all be highlighted as central to the research process and that some text detail should be added to each phase box to provide more information on what each means.
- Evaluation perspectives (shown in the x-axis of Figure 2): many respondents felt that the perspectives that we presented were shown to be mutually exclusive and hierarchical (which was not the intention). There was significant pushback on using the term ‘realist’ as an evaluation perspective. Respondents questioned whether or not we were advocating for evaluations that do not measure effectiveness, with some conflicting views on whether or not this was a positive change. It was felt that there was not enough focus on how the perspectives relate to intervention development or to the development of research questions.
- Framework for addressing complexity within an evaluation (see Figure 2): although some liked this framework, on balance respondents did not feel that this figure complemented the text or was very clear. Complexity does not increase in a linear fashion based on intervention components and perspective taken. Context and system were missing from the diagram despite being a large focus of the text.

##### Research phases (shown Figure 1, plus a section of text was also dedicated to each phase)

- Developing and identifying complex interventions: suggestions included that we consider minimising detail in this section and signpost to the MRC-funded INDEX guidance;^11^ clarify the different circumstances in which development versus identification of interventions is appropriate; and consider including something specific on digital interventions.
- Feasibility: make sure that the definition of feasibility is clear, for example in line with other standard definitions. There was a call for additional detail on the role of context in determining uncertainties for feasibility testing.
- Evaluation: as in the expert workshop, there was conflicting feedback from respondents on how to provide guidance on evaluation. Suggestions included highlighting that evaluations must focus on effectiveness, with additions (not replacements) relating to theory and systems perspectives, but also to include better examples of evaluations focusing on systemic questions. Many respondents felt that there was too much focus on realist evaluation and little mention of theory-based evaluation approaches. The section on study design needs to be clearer, particularly on why some designs are included but others are not.
- Implementation: suggestions were made to differentiate between clinical and implementation interventions; add EPOC (Effective Practice and Organisation of Care) criteria^20^ and diagnostic approaches to implementation; and clarify the time and stage of modification in relation to implementation.

##### Overarching considerations (see Figure 1)

- Programme theory: it was suggested that we provide greater detail on theory-led research, with a balance of signposting to appropriate resources; address how theory-based content is written and presented for readers who come from non-theory-based disciplines, to avoid alienating people; and clarify the terminology relating to ‘mechanism’, ‘programme theory’ and ‘logic models’.
- ‘Modification’ (changed to refinement in the final version): there were conflicting opinions on the use of terminology, particularly with the (MRC-/NIHR-funded) adaptation guidance (in preparation^21^). Respondents called for guidance on where/when to perform modifications and how to agree acceptable boundaries, as well as examples to help readers understand the different approaches to modification.
- Stakeholders: respondents encouraged a greater focus on PPI, more consideration of the challenges of ‘stakeholder engagement’, as well as practical examples of how to engage stakeholders.
- Economic considerations: respondents suggested that we make sure that sensitivity analysis is discussed in relation to statistical models as well as economic models; that we give mention to the tensions between equity and efficiency in evaluating complex interventions; and that we provide more detail on generalisability and context dependency of cost-effectiveness as well as effectiveness, and the possibility of using programme theory to achieve this.

The majority of respondents were positive about the document overall, albeit with constructive criticism that required the project team to reconsider various aspects. Examples of changes that were made following consultation (note that this is not an exhaustive list of changes) were:

- Figures –
  ∘ the framework for addressing complexity in evaluation was removed (see Figure 2)
  ∘ Figure 1 was updated to place ‘context’ in the centre with the other ‘overarching considerations’ (re-termed ‘core elements’) and short descriptors added to each of the phases.
- Evaluation perspectives (see Figure 2) – a rewrite of this section was required (now termed ‘research perspectives’). We changed ‘realist perspective’ to ‘theory-based perspective’ to take account of other approaches to evaluation that aim to explore how and why interventions bring about change.
- Terminology –
  ∘ the definition of ‘complex intervention’ was updated
  ∘ ‘modification’ was changed to ‘refinement’
  ∘ programme theory/logic model – a decision was taken to use ‘logic model’ for the visualisation of the ‘programme theory’, with programme theory detailed with text.
- Programme theory: we have clarified terminology in the text and *Glossary*.
- Modification/refinement: we changed the term from ‘intervention modification’ to ‘intervention refinement’, and differentiated from ‘adaptation’. We have added detail to this section on when you would expect interventions to be refined and why, including a separate section on rapid refinement of digital interventions.
- Stakeholders: we separated the section on stakeholders into PPI and professional stakeholders, and added text to highlight the challenges in engaging stakeholders.
- Economic considerations: we have edited and moved some of this section to other parts of the document to avoid repetition. We add detail on the potential trade-off between equity and efficiency.
- Developing and identifying interventions: we removed text and used the INDEX guidance as the basis for this section, adding three more points that were not highlighted in that guidance but were felt to be important throughout the process of developing this document.
- Feasibility: we further clarify what we mean by ‘feasibility’. We have re-ordered this section to improve readability. We have added a section on ‘efficacy signals’ to further show the potential of feasibility studies.
- Evaluation: we have added detail on how the research perspectives are related to evaluation, as well as more case studies to illustrate the main points. We have emphasised the need for qualitative study in an evaluation and have added detail on process evaluation. We have added detail on the strengths and limitations of each type of economic evaluation.
- Implementation: we have considered separately in this section (1) implementation science research, which focuses specifically on the development and evaluation of interventions to maximise effective implementation; and (2) the need to emphasise implementation considerations in earlier phases, including hybrid effectiveness/implementation designs. In the earlier phases and in the core elements, we have highlighted context, stakeholder input and the need for a broader programme theory, all of which contribute to increased consideration of implementation factors.

### Stage 4: writing the updated framework

#### Methods for stage 4: writing the updated framework

The writing of the framework was led by the project team and was supported by co-authors in the writing group and the SAG. Feedback was received at various stages throughout the writing process from members of the MRC’s Population Health Sciences Group (PHSG) and the MRC–NIHR Methodology Research Programme (MRP) Advisory Group.

Given that the document had changed substantially from the open consultation draft, we asked a further set of external individuals to provide comments on the near-final draft. We received feedback from eight people in May/June 2020. The final draft was then sent to all co-authors for approval.

#### Findings from stage 4: final approval and sign-off

The final draft was approved by the MRC’s PHSG in March 2020.

#### Patient and public involvement

This project was methodological; views of patients and the public were included at the open consultation stage of the update. The open consultation, involving access to an initial draft, was promoted to our networks via e-mail and via digital channels, such as our unit Twitter account (Twitter, Inc., San Francisco, CA, USA; www.twitter.com). We received five responses from people who identified as ‘service users’ (rather than researchers or professionals in a relevant capacity). Their input included helpful feedback on the main complexity diagram, the different research perspectives, the challenge of moving interventions between different contexts and overall readability and accessibility of the document. Several respondents also highlighted useful signposts to include for readers.

In relation to broader PPI, the resulting updated framework (see Chapter 2) highlights the need to include PPI at every phase of developing and evaluating complex interventions. We have drawn on and referred to numerous sources that provide further detail or guidance in how to do so.

#### Limitations

There was a huge amount to cover in developing this document. We have not provided detailed methodological guidance where that is covered elsewhere because we have tried to focus on the main areas of change and novelty. In many of these areas of novelty, methods and experience are in some parts quite limited. In addition, we have foregrounded the very important concept of ‘uncertainties’ and, although there are methods of doing this through, for example, decision-modelling and more qualitative soft system methodologies, this area is limited and specific guidance on how to determine uncertainties in a formal way may seem unclear. We recommend that due consideration is given to this concept and call for further work to develop methods and provide examples in practice. Invariably we may have missed something in our writing and, furthermore, the fields will inevitably move on at pace following publication of this document.

## Conclusion

Parts of this text have been reproduced with permission from Skivington *et al*.^26^ This is an Open Access article distributed in accordance with the terms of the Creative Commons Attribution (CC BY 4.0) license, which permits others to distribute, remix, adapt and build upon this work, for commercial use, provided the original work is properly cited. See: https://creativecommons.org/licenses/by/4.0/. The text below includes minor additions and formatting changes to the original text.

In this document, we have incorporated developments in complex intervention research that were published since the previous edition was written in 2006. We have retained the basic structure of the research process as comprising four phases – development, feasibility, evaluation and implementation – but we emphasise that a programme of research may begin at any of these points, depending on what is already known. We have emphasised that complex intervention research will not always involve the development of new researcher-led interventions, but will often involve the evaluation of interventions that are not in the control of the researcher, but instead led by policy-makers or service managers, or are the adaptation of interventions from another context. We have highlighted the importance of engaging stakeholders throughout the research process, including patients, the public, practitioners and decision-makers. We emphasise the value of working with them as partners in research teams to jointly identify or prioritise research questions; develop, identify or prioritise interventions; and agree programme theories, research perspectives, key uncertainties and research questions.

As with earlier editions, we stress the importance of thorough development and feasibility testing prior to large-scale evaluation studies. As well as taking account of established practice and recent refinements in the methodology of intervention development, feasibility and pilot studies, we draw attention to new approaches, such as evaluability assessment, that can be used to engage stakeholders in collaborative ways of planning and conducting research. We place greater emphasis than in the previous edition on economic considerations in complex intervention research. We see these as a vital to all phases of a research project, rather than simply a set of methods for assessing cost-effectiveness.

We have introduced a new emphasis on the importance of context and the value of understanding interventions as ‘events in systems’ that produce effects through interactions with features of the contexts in which they are implemented. We adopt a pluralist approach and encourage consideration and use of diverse research perspectives, namely efficacy, effectiveness, theory-based and systems perspectives, and the pragmatic choice of research questions and methods that are selected to optimally address the key uncertainties that remain. We acknowledge that to generate the most useful evidence for decision-making will often require a trade-off between precise, unbiased answers to narrowly defined questions and less certain answers to broader, more complex questions.

Although we have not explicitly discussed epistemology, we have challenged the position established in earlier editions that unbiased estimates of effectiveness are the cardinal goal of evaluation, and we have emphasised that improving theories and understanding of how and in what circumstances interventions contribute to change is also an important goal for complex intervention research. For many complex intervention research problems, an efficacy or effectiveness perspective will be the optimal approach, for which a RCT will probably provide the best design to achieve an unbiased estimate. For other problems this will not be the case, and alternative perspectives and designs will be more likely to generate useful new knowledge to help reduce decision-maker uncertainty. What is important for the future is that the scope of intervention research commissioned by funders and undertaken by researchers is not constrained to a limited set of perspectives and approaches that may be less risky to commission and more likely to produce a clear and unbiased answer to a specific question. What is needed is a bolder approach, including some methods and perspectives for which experience is still quite limited, where we (supported by our workshop participants and respondents to our consultations) believe that there is an urgent need to make progress by mainstreaming new methods that are not yet widely used, as well as undertaking methodological innovation.

We have emphasised the importance of continued deliberation by the research team of what the key uncertainties are that are relevant to that stage of research, and then defining research questions and selecting research perspectives and methods that will reduce that uncertainty. We reiterate that our recommendation is not to undervalue research principally designed to minimise bias in the estimation of effects; rather, we encourage the use of a wider range of perspectives and methods, augmenting the available toolbox and, thus, increasing the scope of complex intervention research and maximising its utility for decision-makers. This more deliberative, flexible approach is intended to reduce research waste and increase the efficiency with which complex intervention research generates knowledge that contributes to health improvement.

We acknowledge that some readers may prefer more detailed guidance on the design and conduct of any specific complex intervention research project. The approach taken is to help researchers identify the key issues that ideally need to be considered at each stage of the research process, to help research teams choose research perspectives and prioritise research questions, and to design and conduct research with an appropriate choice of methods. We have not provided detailed methodological guidance, primarily because that is well covered elsewhere. We have been fortunate to be able to draw on and refer to many other guidance documents that address specific and vitally important aspects of the complex intervention research process and specific aspects of research design, conduct and reporting. We encourage researchers to consult these sources, which provide more detail than we were able to here. We have provided more emphasis and detail in areas of change and novelty introduced in this edition. However, in many of these areas there is an urgent need for further methods development and guidance for their application and reporting in complex health intervention research. These include more formal methods to quantify or consider uncertainty, for example decision-modelling approaches, Bayesian approaches, uncertainty quantification or more qualitative soft systems methodologies, and methods suited to a systems perspective including simulation approaches and qualitative comparative analysis methods.

### Recommendations

The recommendations of this work are given in *Chapter 2*. At the end of each research phase section (see *Chapter 2*, *Phases of research*) we include a table of elements that we recommend should be considered at that phase. The overall recommendation, therefore, is that people use the tables at the end of each phase when developing research questions and use the checklist in Appendix 6 as a tool to record where/how the recommendations have been followed.

Monitoring the use of this framework and evaluating its acceptability and impact is warranted: this has been lacking in the past. We encourage research funders and journal editors to support the diversity of research perspectives and methods that are advocated and to seek evidence that the key considerations are attended to in research design and conduct. The use of the checklist that we provide to support the preparation of funding applications, research protocols and journal publications (see Appendix 6) offers one way to monitor impact of the framework on researchers, funders and journal editors. Further refinement of the checklist is likely to be helpful.

We recommend that future updates of this framework continue to adopt a broad, pluralist perspective. Given the widening scope and the rich, diverse and constantly evolving body of detailed methods guidance that is now available on specific methods and topics, the framework will most usefully be in the form of a high-level framework with signposting, published in a fluid, web-based format, which will ideally be frequently updated to incorporate new material, both through updates to the text and case studies and through the addition of new links to updated and emerging key resources.

## Data Availability

Owing to this project being methodological in nature there is no data that can be shared. For more information please contact the corresponding author.
