# Supplementary material for: Framework for the development and evaluation of complex interventions: gap analysis, workshop and consultation-informed update
Source: Health Technol Assess. Author manuscript; Available in PMC 2023 Jan 4. (PMC7614019; doi:10.3310/hta25570)
Supplement: Appendix [file EMS157731-supplement-Appendix.pdf]

Appendix 1 Oversight of the project

Who	Role
Project team	To undertake all aspects of the project including leading the data gathering, data analysis and writing the framework document
SAG (see *Appendix 3* for members)	To act as the oversight body for the work, specifically to:<br>• provide expert advice to the project team<br>• monitor progression of the project in relation to overall timelines<br>• approve the proposed methodology<br>• approve the membership of the writing group<br>• approve/comment on the publication policy<br>• approve/comment on the main project outputs<br>• contribute to the promotion and dissemination of the updated framework<br>• follow the terms of reference as agreed
Writing group (authors)	To be part of the author group for the work, adhering to specified authorship criteria. This included:<br>• providing written and critical commentary related to area of expertise<br>• delivering a significant contribution to the main project outputs
MRC PHSG and MRP Boards	Reviewed and approved the framework

Copyright © 2021 Skivington *et al.* This work was produced by Skivington *et al.* under the terms of a commissioning contract issued by the Secretary of State for Health and Social Care. This is an Open Access publication distributed under the terms of the Creative Commons Attribution CC BY 4.0 licence, which permits unrestricted use, distribution, reproduction and adaption in any medium and for any purpose provided that it is properly attributed. See: https://creativecommons.org/licenses/by/4.0/. For attribution the title, original author(s), the publication source – NIHR Journals Library, and the DOI of the publication must be cited.

Appendix 2 Updating the Medical Research Council guidance on complex interventions: an outline proposal for developing an integrated body of guidance

1. Background: the need for updating and integrating the MRC guidance

The MRC guidance on evaluating complex interventions (CIs) was first published in 2000, and updated in 2008. Over this period, interest in complex interventions has grown rapidly, and more specialised guidance has been published or is being developed in several related areas, including intervention development, intervention description, process evaluation, natural experimental approaches, dealing with intervention context, group-delivered interventions, realist trials, surgical trials, exploratory trials, trial management, disability research and knowledge translation [see **References** for examples]. A number of other areas that were identified as gaps in the 2008 guidance, such as research priority-setting and the application of complex systems science to health interventions, have also attracted interest, but are not yet covered by accessible guidance for producers or users of evidence. Both the 2000 and 2008 core guidance documents continue to be highly cited (figure), but given the pace and extent of methodological development, there is a strong case for updating the core guidance, linking it with related developments, and also addressing some of the remaining weaknesses and gaps in the existing guidance.

Aim & objective: To identify and summarise aspects of the 2006 MRC complex intervention guidance that require updating, with the aim of using this gap analysis as a starting point for discussion (through workshops and authorship group) to achieve consensus on focus points and resulting updates for the new guidance.

Copyright © 2021 Skivington *et al.* This work was produced by Skivington *et al.* under the terms of a commissioning contract issued by the Secretary of State for Health and Social Care. This is an Open Access publication distributed under the terms of the Creative Commons Attribution CC BY 4.0 licence, which permits unrestricted use, distribution, reproduction and adaption in any medium and for any purpose provided that it is properly attributed. See: https://creativecommons.org/licenses/by/4.0/. For attribution the title, original author(s), the publication source – NIHR Journals Library, and the DOI of the publication must be cited.

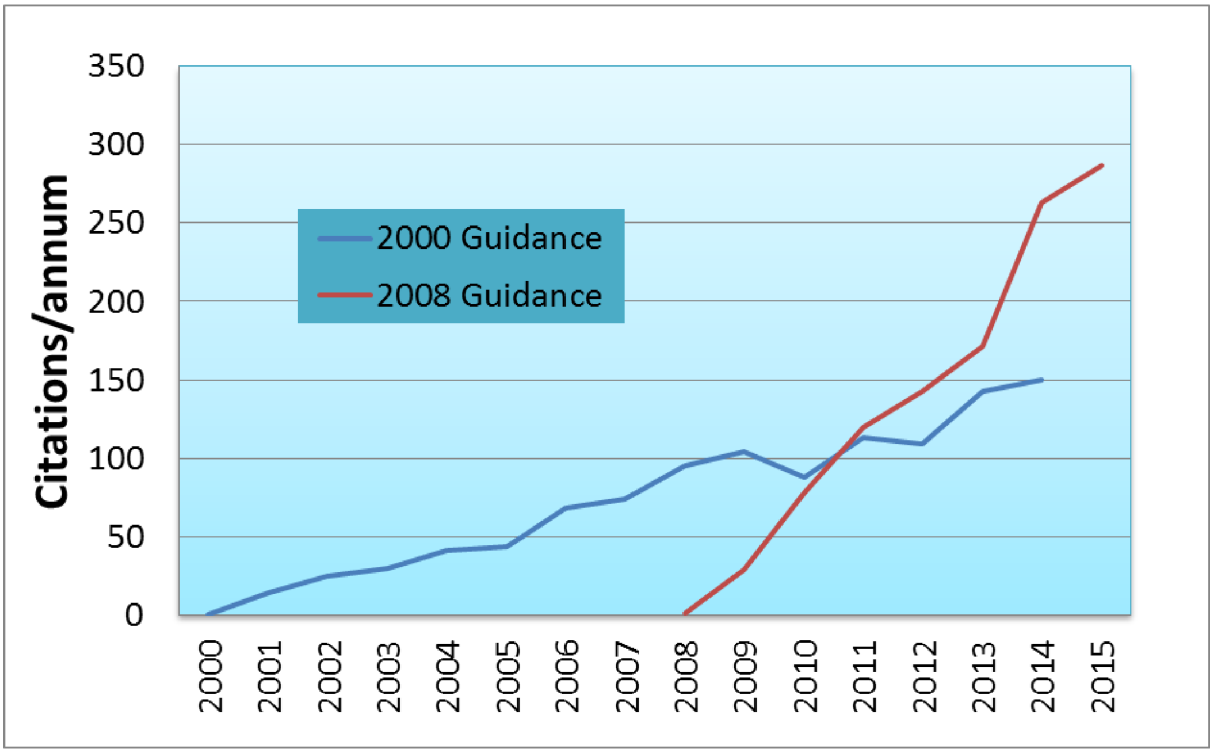

Source: Web of Science. The '2000 Guidance' refers to Campbell *et al*. (2000) and the '2008 Guidance' refers to Craig *et al*. (2008).

2. Proposed outputs

The 2000 and 2008 guidance each comprised a long version, published online by the MRC, and a short version published by the BMJ. The MRC's process evaluation and natural experiments guidance followed a similar model. Citations of the short versions greatly outnumber citations of the long versions, although the longer versions have important additional detail and have a very high number of downloads. For the updated guidance we recommend two products to disseminate the updated core guidance:

a) A **downloadable pdf** version of the core guidance.
b) A **journal article** (ideally published simultaneously in a number of journals, as the CONSORT statements are) which would describe the importance and need for the guidance as well as outlining its content, also referring potential users to the detailed online pdf version and signposting resource.

In addition, we will continue discussions about creating an online resource comprising the updated core guidance with links to signpost other related source documents (e.g. the MRC's process or natural experiments guidance) or to brief summary statements prepared specifically, with links to other useful published resources already available online.

3. Proposed stages of development

- *Gap analysis*: based on scoping reviews to find publications that identify gaps and weaknesses in the existing guidance, or that provide more detailed guidance on specific topics. This will take the form of *horizon scanning and further literature review:* a brief scoping review will be followed by discussion at the Scientific Advisory Group (SAG) meeting (24/11/17). Our initial search (google scholar, forward/backward citations) will focus on: (a) New approaches/progress since previous guidance; (b) Criticisms of existing guidance; (c) Other gaps. Prior to the SAG meeting we will develop a brief list of topics to address in the updated guidance. We will present these topics to the SAG (by sending a summary in advance and by presentation of an overview at the meeting). Following discussion at the SAG meeting we will finalise a list of topics that we will explore in more depth.
- *Expert workshop:* The findings from the gap analysis will inform the agenda for an expert workshop to be held in early 2018. Each of the 'topics/themes' identified for update should be represented (by an expert) at the workshop i.e. we will invite experts based on these themes. The aim of the expert workshop is to achieve consensus on topics that should be newly covered or updated by the new guidance and as a basis for the project team, along with the rest of the authorship group, to produce the updates and additions. Follow-up consultation (email and a consensus meeting) will be used to achieve consensus on the details of the updated guidance.
- *Identify relevant case studies:* Worked examples of the development, implementation, or evaluation of a complex intervention. This will be started at the workshop and finalised through consultation afterwards.
- Convening a steering group to oversee the work, review and approve drafts, and ensure stakeholder commitment; and a writing group to draft the update and linked summaries
- Drafting the update and summaries
- Drafting the journal article and managing the publication process

4. Representation

(a) Steering group: The steering group will meet on an infrequent basis (e.g. two or three times over timeline of activity). The steering group members are: (chair) Martin White (NIHR Public Health Research Programme), David French (MRC–NIHR Methodology Research Programme), Jo Rycroft-Malone (NIHR Health Services Delivery Research Programme), Mark Petticrew (Co-author of previous guidance), Martin Ashton-Key (NIHR HTA Programme & Consultant Adviser, NETSCC), Janis Baird (Co-author of previous process evaluation guidance), Jane Blazeby (MRC Hubs for Trial Methodology Research), Samuel Rowley (Observer), Gavin Malloch (Observer). All will be offered authorship (alongside formal authorship criteria).

(b) Writing group: The writing group will meet on a more regular basis (tbc), be engaged in the progress of the guidance and contribute to the writing process. Members of the writing group will be acknowledged as authors on the final guidance. The project team will be members of the writing group, with additional authors identified as appropriate, e.g. to fill gaps in expertise.

(c) **Project team:** Employed researchers Kathryn Skivington & Lynsay Matthews; senior staff from the host institution Sharon Simpson, Peter Craig, Laurence Moore.

Copyright © 2021 Skivington *et al.* This work was produced by Skivington *et al.* under the terms of a commissioning contract issued by the Secretary of State for Health and Social Care. This is an Open Access publication distributed under the terms of the Creative Commons Attribution CC BY 4.0 licence, which permits unrestricted use, distribution, reproduction and adaption in any medium and for any purpose provided that it is properly attributed. See: https://creativecommons.org/licenses/by/4.0/. For attribution the title, original author(s), the publication source – NIHR Journals Library, and the DOI of the publication must be cited.

References

Craig, P., C. Cooper, et al. (2012). "Using natural experiments to evaluate population health interventions: new Medical Research Council guidance." Journal of Epidemiology and Community Health 66: 1182-1186.

Craig P, Dieppe P, Macintyre S, et al. Developing and evaluating complex interventions: the new Medical Research Council guidance. BMJ 2008;337:a1655.

Hoddinott, P., Allan, K., Avenell, A., Jane Britten, J., 2010. Group interventions to improve health outcomes: a framework for their design and delivery. BMC Public Health 10, 800-9.

Hoffmann, T. C., P. P. Glasziou, et al. (2014). "Better reporting of interventions: template for intervention description and replication (TIDieR) checklist and guide." BMJ 348.

Campbell M, Fitzpatrick R, Haines A, Kinmonth AL, Sandercock P, Spiegelhalter D, et al. Framework for the design and evaluation of complex interventions to improve health. *BMJ* 2000;321:694-6.

McCulloch, P., Altman, D.G., Campbell, W.B., Flum, D.R., Glasziou, P., Marshall, J.C., Nicholl, J., for the Balliol Collaboration, 2009. No surgical innovation without evaluation: the IDEAL recommendations. Lancet 374, 1105–12.

Moore G et al 2015. Process evaluation of complex interventions. Medical Research Council Guidance. BMJ 350:h1258. doi:10.1136/bmj.h1258.

Treweek, S Altman, D, Bower, P et al. (2015) Making randomised trials more efficient: report of the first meeting to discuss the Trial Forge platform. Trials 16:261

Appendix 3 Advisors/consultees

Members of the Scientific Advisory Group

Martin White (chairperson), Martin Ashton-Key, Janis Baird, Jane M Blazeby, David P French, Mark Petticrew and Jo Rycroft-Malone.

Participants of the expert workshop

Jean Adams, Rob Anderson, Till Baernighausen, Janis Baird, Jane Blazeby, Chris Bonell, Kathleen Boyd, Rona Campbell, Neil Craig, Steven Cummins, Frank DeVocht, Matt Egan, Matt Field, David P French, Michael Grayling, Judith Green, Marie Johnston, Frank Kee, Mike Kelly, Natasha Kriznik, James Lewsey, Alastair Leyland, Ronan Lyons, Petra Meier, Susan Michie, Graham Moore, Jane Noyes, Chris Owen, Mark Petticrew, Harry Rutter, Jo Rycroft-Malone, Falko Sniehotta, David Taylor-Robinson, Rebecca Walwyn, Katrina Wyatt and Lucy Yardley.

In addition, input was received from (although invited, could not attend the workshop) Marion Campbell, Ruth Dundas, Pat Hoddinot, Russ Jago, Alicia O'Cathain and Eva Annette Rehfuess.

Systems meeting

Steve Cummins, Matt Egan, Vanessa Er, Mike Kelly, Karen Lock and Harry Rutter (follow-up meeting only).

Intervention development meeting

Graham Moore, Alicia O'Cathain, Lucy Yardley and Ed Duncan.

Provided final feedback

Matt Egan, Matti Heino, Graham Moore, Dave Richards, Mark Robinson, Mike Robling and Jeremy Segrott.

Copyright © 2021 Skivington *et al.* This work was produced by Skivington *et al.* under the terms of a commissioning contract issued by the Secretary of State for Health and Social Care. This is an Open Access publication distributed under the terms of the Creative Commons Attribution CC BY 4.0 licence, which permits unrestricted use, distribution, reproduction and adaption in any medium and for any purpose provided that it is properly attributed. See: https://creativecommons.org/licenses/by/4.0/. For attribution the title, original author(s), the publication source – NIHR Journals Library, and the DOI of the publication must be cited.

Appendix 4 Online consultation questionnaire

Q1. Please indicate which stakeholder category you belong to (tick one or more boxes)	Researcher
	Funder
	Journal editor
	Practitioner
	Policy-maker
	Service user (Patient or Public)
	Other (please specify)
Q2. What is your main field of expertise? (tick one or more boxes)	Public health
	Clinical medicine
	Health services research
	Health technology assessment
	Policy-making
	Systems-based research
	Patient or public involvement
	Other (please specify)

Q3. Figure 2: This diagram presents the key elements for developing and evaluating complex interventions. Is this diagram a useful way of capturing the research process (please elaborate)? [TEXT BOX HERE]

Q4. Definition of complexity and research perspectives: The updated framework presents a new definition of complexity, and suggests different perspectives of the research process. How useful is it to have the framework for addressing complexity presented in this way? [TEXT BOX HERE]

Q5. Figure 3. This figure relates to the concept of using different research perspectives (as noted in Q3). Does *Figure 3* make sense (please elaborate)? [TEXT BOX HERE]

Q6. Missing elements: If you think there are key elements missing from the guidance, can you briefly outline what they are? [TEXT BOX HERE]

Q7. Signposting to other guidance: We are keen to signpost to further guidance where appropriate. If we have missed any specific guidance that may be helpful to readers can you please note it here, thank you. [TEXT BOX HERE]

Q8. Clarity: Was anything in the guidance particularly unclear (please elaborate)? [TEXT BOX HERE]

Q9. Case studies: We are collating case studies to include in the guidance. We would welcome suggestions in the following areas:

- Modifying interventions to improve their intervention design and/or evaluation design
- The use of programme theory throughout the research process
- Involvement of stakeholders throughout the research process
- Economic considerations throughout the research process
- The exploration and use of context throughout the research process
- Addressing uncertainties throughout the research process
- Complexity informed approaches to intervention research

Q10. Other feedback: If you have other comments that may be useful as we finalise the guidance, please provide brief details [TEXT BOX HERE]

Copyright © 2021 Skivington *et al.* This work was produced by Skivington *et al.* under the terms of a commissioning contract issued by the Secretary of State for Health and Social Care. This is an Open Access publication distributed under the terms of the Creative Commons Attribution CC BY 4.0 licence, which permits unrestricted use, distribution, reproduction and adaption in any medium and for any purpose provided that it is properly attributed. See: https://creativecommons.org/licenses/by/4.0/. For attribution the title, original author(s), the publication source – NIHR Journals Library, and the DOI of the publication must be cited.

Appendix 5 Approaching complexity in evidence synthesis

The synthesis of complex intervention studies can be particularly challenging and there are numerous important considerations applicable to conducting evidence synthesis of complex interventions, including (but certainly not limited to: see *Signposts to further reading 12* for detailed discussion and guidance):

- Context – a similar intervention could be implemented in different contexts with very different results; therefore, in synthesising these results it may be important to consider the context in which they were achieved. There are different ways of taking account of context within evidence syntheses: see *Signposts to further reading 12* for further reading. In terms of developing the protocol for a complex intervention evidence synthesis, it may be necessary to adapt traditional frameworks, for example to extract data to consider contextual complexity, such as on timing and setting, dependent on each review's aims.[338]
- Uncertainty – similar to undertaking primary intervention research, whether or not it makes sense to take a systems perspective is dependent on what the users of the review actually want to know, that is where their uncertainty lies and their related priorities.[89,339–341]
- Stakeholder engagement – taking account of multiple perspectives is key to understanding complexity; stakeholders can be involved in each stage, for example determining the scope of the review, framing and defining the review questions, understanding context and interpreting results. It is also important to ensure that the review is relevant and useful to the needs of those with an interest in using the findings, preventing research waste.[341,342]
- Theory-based review – a different approach to the standard evidence synthesis is a 'theory-led' approach, for example a realist review.[343] Realist synthesis takes a different approach to systematic review in that the focus is on coming to an understanding about the theory behind the interventions, exploring the circumstances in which interventions work or do not work, and for whom. The Cochrane Handbook also provides guidance to support the synthesis of studies aiming to increase understanding of intervention complexity, for example through synthesis of qualitative evidence.[344]

Useful discussions and guides about approaching complexity in evidence syntheses have been published; see the box below for further details.

SIGNPOSTS TO FURTHER READING 12 Complexity in systematic reviews

- A special issue in *BMJ Global Health* on concepts and methods for evidence-informed health decisions,[345] including:
  - Determining whether or not a complex systems perspective would be useful for your evidence synthesis: consider the questions laid out by Petticrew *et al.*[89] in box 2 of their paper.
  - Considering complexity within qualitative evidence synthesis[346,347] and in mixed-methods research.[348]
  - Tools and frameworks for considering context within evidence synthesis.[349]
  - WHO-INTEGRATE: a new evidence-to-decision framework tool, particularly developed to be used with complex interventions, to support the transparent consideration of all decision criteria.[350]
  - Special issues of the *Journal of Epidemiology and Community Health*: one largely devoted to considering complexity in systematic reviews of interventions[351] and one to complex interventions in systematic reviews developed by the Agency for Healthcare Research and Quality.[352]
  - A tool for assessing the complexity of interventions within systematic reviews: intervention complexity assessment tool for systematic reviews.[353]

Copyright © 2021 Skivington *et al.* This work was produced by Skivington *et al.* under the terms of a commissioning contract issued by the Secretary of State for Health and Social Care. This is an Open Access publication distributed under the terms of the Creative Commons Attribution CC BY 4.0 licence, which permits unrestricted use, distribution, reproduction and adaption in any medium and for any purpose provided that it is properly attributed. See: https://creativecommons.org/licenses/by/4.0/. For attribution the title, original author(s), the publication source – NIHR Journals Library, and the DOI of the publication must be cited.

SIGNPOSTS TO FURTHER READING 12 Complexity in systematic reviews (*continued*)

- Version 6.1 of the Cochrane Handbook includes new chapters on intervention complexity, qualitative evidence, network meta-analysis and synthesising findings using alternative methods to meta-analysis.[344]
- PRISMA-CI is an extension of PRISMA, the accepted standard for encouraging consistency and transparency in systematic reviews.[354] PRISMA-CI incorporates issues specific to complex interventions, alongside an ‘explanation and elaboration’ guideline to support its use.[355,356]
- Meta-ethnography can inform the development, evaluation and implementation of complex interventions. The eMERGe reporting guidance aims to improve the reporting of meta-ethnography.[357]
- GRADE is being adapted to address non-randomised studies of complex interventions, alongside guidance on how sources of complexity can be considered when using GRADE to rate certainty of evidence.[358]

GRADE, Grading of Recommendations Assessment, Development and Evaluation; PRISMA, Grading of Recommendations Assessment, Development and Evaluation; PRISMA-CI, Grading of Recommendations Assessment, Development and Evaluation – Complex Interventions.

Appendix 6 Checklist for developing and evaluating complex interventions

This checklist is intended as a tool to help researchers prepare funding applications, research protocols and journal publications. It may also help reviewers to assess whether or not the recommendations have been followed.

Item	If NO, please justify. If YES, briefly describe how this has been addressed	Reported on page number(s)
**Addressing uncertainties**		
1. Have you determined the aim(s)/purpose(s) of the intervention?		
2. Have you identified the key uncertainties given existing evidence about the intervention and the context in which it will be tested or implemented?		
3. Do the research questions and methods address the key uncertainties?		
4. Does the choice of research perspective (efficacy, effectiveness, theory-based, systems) reflect the key uncertainties that have been identified?		
**Engaging stakeholders**		
1. Have you engaged stakeholders in the design/identification of the intervention and the development of the research protocol?		
2. Have you engaged stakeholders in the conduct of the research and the dissemination of findings?		
3. Have all stakeholders declared any potential conflicts of interest?		
**Considering context**		
1. Have you identified all the dimensions of context that may influence how the intervention achieves its effects?		
2. Have you considered how context may affect the scaling up or scaling out of the intervention?		
**Developing and refining programme theory**		
1. Have you developed a programme theory for your intervention that describes the key components and mechanisms of the intervention and how it interacts with the context in which it will be implemented?		
2. Have you updated the programme theory to incorporate the new evidence gathered by the study?		
**Refining the intervention**		
1. Have you refined the intervention so that it is optimised for the context in which it will be implemented?		
2. Have you specified how far and in what ways the intervention can be refined during implementation without undermining the programme theory?		
**Economic considerations**		
1. Have you considered whether or not the value of the evidence, in terms of informing future decision-making, justifies the cost of the research?		
2. Have you identified an economic evaluation framework that is appropriate to the expected outcomes of the intervention?		

Copyright © 2021 Skivington *et al.* This work was produced by Skivington *et al.* under the terms of a commissioning contract issued by the Secretary of State for Health and Social Care. This is an Open Access publication distributed under the terms of the Creative Commons Attribution CC BY 4.0 licence, which permits unrestricted use, distribution, reproduction and adaption in any medium and for any purpose provided that it is properly attributed. See: https://creativecommons.org/licenses/by/4.0/. For attribution the title, original author(s), the publication source – NIHR Journals Library, and the DOI of the publication must be cited.

Item	If NO, please justify. If YES, briefly describe how this has been addressed	Reported on page number(s)
**Phase-specific considerations**		
Developing interventions – have you used a formal framework (such as INDEX) to guide development of the intervention?		
Identifying interventions – for policy and practice interventions, have you performed an evaluability assessment to determine whether or not and how an evaluation should be undertaken?		
Feasibility – have you defined and used clear progression criteria to guide decisions about whether to proceed to an evaluation study?		
Evaluation – have you chosen an appropriate study design to answer the research questions and provide robust evidence to inform decision-making about further intervention refinement, evaluation or implementation?		
Implementation – have constraints and enablers of implementation been considered at all phases, from intervention development, through feasibility and effectiveness testing, to large-scale roll-out?		

EME
HS&DR
HTA
PGfAR
PHR

Part of the NIHR Journals Library
www.journalslibrary.nihr.ac.uk

This report presents independent research funded by the National Institute for Health Research (NIHR). The views expressed are those of the author(s) and not necessarily those of the NHS, the NIHR or the Department of Health and Social Care

Published by the NIHR Journals Library
