# Supplementary material for: Framework for the development and evaluation of complex interventions: gap analysis, workshop and consultation-informed update
Source: Health Technol Assess. Author manuscript; Available in PMC 2023 Jan 4. (PMC7614019; doi:10.3310/hta25570)
Supplement: Related texts

# Book: Chapter 2: The new framework

## Introduction

### Aims of the framework

The framework aims to improve the design and conduct of complex intervention research to increase its utility, efficiency and impact. Consistent with the principles of increasing the value of research and minimising research waste,^22^ the framework (1) emphasises the use of diverse research perspectives and the inclusion of research users, clinicians, patients and the public in research teams, and (2) aims to help research teams prioritise research questions and choose and implement appropriate methods.

### Structure of the framework

The framework is presented as follows:

- In *What is a ‘complex intervention’?*, we provide an updated definition of ‘complex intervention’ informed by a broader understanding of complexity, and introduce how different research perspectives can be employed in complex intervention research.
- In *Framework for the main phases and core elements of complex intervention research*, we set out the revised framework for developing and evaluating complex interventions and provide an overview of the key core elements that we recommend are considered repeatedly throughout the phases of complex intervention research.
- In *Phases of research*, we go into further detail about each of the key phases in complex intervention research.
- In *Case studies*, we present illustrative case studies to help exemplify aspects of the framework using a variety of study designs, from a range of disciplines, undertaken in a range of settings.

### How to use the framework

In the 2000^2^ and 2006^1^ versions of this document, there was a key explanatory diagram in which complex intervention research was divided into a number of key phases. In this edition, we have developed a revised explanatory diagram (see Figure 3). This does not offer a linear or even cyclical pathway through the phases of development and evaluation of interventions, but emphasises that at any phase key core elements should be considered to guide decisions as to whether or not the research should proceed to the next phase, return to a previous phase, repeat a phase or be aborted. The framework encourages teams to consider the research perspective(s) most suited to the research challenge that they are aiming to address and to use the six core elements to guide the choices that they make at each research phase. A programme of intervention research may begin at any one of the four phases, depending on the key uncertainties that are associated with the intervention and taking into account existing evidence and/or experience. Users can, therefore, choose which sections of the recommendations are most appropriate to their own research plans. To aid navigation through the document, we provide extensive hyperlinks to cross-references between sections. Throughout the document, we refer to existing detailed guidance and provide key points and signposts to further reading. At the end of the sections on each phase of research (see Tables 4–8) we include a table that lists the core considerations relevant to that phase, which we recommend to be used as a tool in research planning.

Given that this is a pragmatic framework aimed at an audience from multiple disciplines, we have taken a pluralist approach. Terminology that is related to the study of complex interventions is not used consistently across disciplines; our use of terms is detailed in the *Glossary*. Note that we focus on evaluation rather than ex ante appraisal,^23^ and specifically on evaluations that contribute to the scientific evidence base, rather than those that are conducted primarily for monitoring and accountability purposes.

Previous guidance has focused on statistical and qualitative considerations; we refer to this as well as highlight the importance of statistical and qualitative considerations throughout the research process. Economic issues in the context of developing and evaluating complex interventions have been given less attention; however, their consideration is also important from the early phases of research in relation to intervention development, evaluation and future implementation. We, therefore, provide more detail in this section of the framework.

We focus on primary research but note that other types of research, in particular evidence synthesis, must consider how to approach complex interventions as well. In Appendix 5, we provide some brief points and signposts to further reading on approaching complexity for producers and users of evidence syntheses. In Appendix 6, we provide a checklist to support and document the use of this framework in the preparation of funding applications and journal articles.

## What is a ‘complex intervention’?

Few interventions are truly simple. Complexity arises from the properties of the intervention itself, the context in which an intervention is delivered and the interaction between the two.^8,24,25^

### Sources of complexity

#### Complexity owing to characteristics of the intervention

Parts of this text have been reproduced with permission from Skivington *et al*.^26^ This is an Open Access article distributed in accordance with the terms of the Creative Commons Attribution (CC BY 4.0) license, which permits others to distribute, remix, adapt and build upon this work, for commercial use, provided the original work is properly cited. See: https://creativecommons.org/licenses/by/4.0/. The text below includes minor additions and formatting changes to the original text.

Interventions become more complex in line with increasing the:^1^

- number of intervention components and the interactions between them
- range of behaviours, expertise and skills (e.g. particular techniques and communication) required by those delivering or receiving the intervention
- number of groups, organisational levels or settings that are targeted by the intervention
- level of flexibility or tailoring of the intervention or its components that is permitted (i.e. how dynamic or adaptive the intervention is).

For example, the Links Worker Programme^27^ was an intervention in primary care in Glasgow, Scotland, that aimed to link people with community resources that could help them to ‘live well’ in their communities. It targeted individual, primary care [general practitioner (GP) surgery] and community levels; the intervention was flexible in that it could differ between primary care GP surgeries. In addition, there was no single health or well-being issue that the Link Workers specifically supported: bereavement, substance use, employment and learning difficulties were all things that could be included.^27^ The inherent complexity of this intervention had implications for many aspects of its evaluation, such as the choice of appropriate outcomes.

#### Complexity arising from context

Context: *… any feature of the circumstances in which an intervention is conceived, developed, implemented and evaluated*.

*Reproduced with permission from Craig* et al.^10^*Contains information licensed under the Non-Commercial Government Licence v2.0*

Examples of features include social, political, economic and geographical contexts. Whether or not and how an intervention generates outcomes can be dependent on a wide range of contextual factors. These contextual factors will be intervention specific and may be difficult to anticipate. An example of an intervention that may seem simple, until context is considered, is the ‘Lucky Iron Fish’ (Box 1).^10^

It is important to consider what features of context may be important in determining how an intervention achieves outcomes and under what circumstances an intervention may be more or less effective. This is important for all phases, that is for developing a new intervention, adapting or translating an existing intervention to a new context, evaluation and implementation.

#### Interventions as ‘events in systems’

System: a set of things that are interconnected in such a way that they produce their own pattern of behaviour over time.

Meadows and Wright^31^

Systems thinking can help us to understand the interaction between an intervention and the context in which the intervention is implemented in a more dynamic way. Systems can be thought of as complex and adaptive,^32^ in that they are defined by system-level properties, such as feedback, emergence, adaptation and self-organisation (Table 2). We can theorise interventions as ‘events in systems’^24^ and can conceive an intervention’s outcomes as being generated through the interdependence of the intervention and a dynamic system context.

Change in complex systems may be unpredictable. In a social system, people interact with each other and other parts of the system in non-linear and interconnected ways so that the actions of one person alter the context for others.^35^ For example, removing hospital car-parking charges has clear beneficiaries. However, by encouraging people to drive, the policy may reduce demand for public transport, leading to a reduction or withdrawal of services. The net effect may be to reduce accessibility to those without a car. Demand for car parking will increase, possibly beyond capacity, and alternative options will have diminished. What might appear at first sight to be a positive intervention may have adverse effects on health and serve to widen inequalities.

**Signposts to Further Reading 1 Systems thinking in health research**

- For a more detailed accessible introduction to applying systems thinking to public health evaluation, see Egan *et al*.^13,14^
- The Health Foundation’s overarching description and evidence scan.^32^
- Understanding complexity in health systems: international perspectives. A series of academic papers on the topic.^8^
- The Magenta Book^36^ is from the UK Government and provides guidance on the evaluation of government interventions, with recommendations for the planning, conduct and management of the evaluation. It provides relevant guidance on complex systems-informed evaluation and a supplementary guide that specifically focuses on handling complexity in policy evaluation. This highlights the challenges of complexity to policy evaluation and the importance of its consideration in commissioning and managing interventions, including guidance on the approaches available to support such projects.^36^
- A report published by the Centre for Complexity Across the Nexus and commissioned by the Department for Environment, Food and Rural Affairs provides a framework for evaluation, specifically to support evaluations of government policy to consider the implications of complexity theory.^37^

**Table 2:** Properties of complex adaptive systems, with examples

Properties of complex adaptive systems	Example
***Emergence***	
Complex systems have emergent properties that are a feature of the system as a whole. Emergent properties are often unanticipated, arising without intention	Group-based interventions that target at-risk young people may be undermined by the emergence of new social relationships among the group that increase exposure to and reinforce risk behaviours, while reducing their contact with mainstream youth culture where risk-taking is less tolerated^33^ Enhanced recovery pathways are introduced to optimise early discharge and improve patient outcomes. They involve changes across pre-operative care where patient expectations are managed; changes in theatre by minimising the length of surgical incisions and the use of surgical drains; and changes in postoperative care on surgical wards (e.g. the use of physiotherapy). As enhanced recovery pathways are introduced for specific patients it influences management of other patients; thus, the whole culture of surgical practice within a hospital changes towards ‘enhanced recovery’
***Feedback***	
Where one change reinforces, promotes, balances or diminishes another	A smoking ban in public places reduces the visibility and convenience of smoking. Fewer young people start smoking because of its reduced appeal, thus further reducing its visibility and so on in a reinforcing loop^7^
***Adaptation***	
***A*** change of system behaviour in response to an intervention	Retailers adapted to the ban on multibuy discounts for alcohol by placing discounts on individual alcohol products, offering them at the same price individually as they would have been if part of a multibuy offer^34^
***Self-organisation***	
Organisation and order achieved as the product of spontaneous local interaction rather than of a preconceived plan or external control	Individually focused treatment for people who misuse alcohol did not address some social aspects of alcohol dependency; as a result, recovery groups were self-organised in a collective effort and Alcoholics Anonymous (New York, NY, USA) was formed

Reproduced with permission from Skivington *et al.^26^* This is an Open Access article distributed in accordance with the terms of the Creative Commons Attribution (CC BY 4.0) license, which permits others to distribute, remix, adapt and build upon this work, for commercial use, provided the original work is properly cited. See: https://creativecommons.org/licenses/by/4.0/. The table includes minor additions and formatting changes to the original table.

## Complexity and the research perspective

We aim to encourage wider awareness, understanding and use of ‘complexity-informed’ research,^8^ by which we mean research that gives sufficient and appropriate consideration to all of the sources of complexity outlined in the previous section. There are several overlapping perspectives that can be employed in complex intervention research (Box 2), each associated with different types of research questions. Examples of complex intervention studies taking different research perspectives are given at the end of this subsection (see Box 4).

### Efficacy perspective

Research taking an efficacy or effectiveness perspective is principally concerned with obtaining unbiased estimates of the average effect of interventions on predetermined outcomes. Studies from an efficacy perspective aim to test hypotheses about the mechanisms of action of interventions.^38^ Such research aims for high internal validity, taking an explanatory approach to test causal hypotheses about the outcome(s) generated by the intervention. This contrasts with effectiveness studies, which take a more pragmatic approach. The PRagmatic Explanatory Continuum Indicator Summary (PRECIS)-2 tool^39^ identifies nine domains on which study design decisions may vary according to the perspective or approach. Few studies exhibit all of the characteristics of a purely efficacy or a purely effectiveness perspective; there is not necessarily a dichotomy between efficacy and effectiveness studies, rather a continuum.

Studies from an efficacy perspective will typically evaluate interventions in idealised, controlled conditions among a homogeneous group of highly selected participants using a proximal outcome, such as disease activity, with the intervention delivered in a standardised manner with high fidelity to a protocol or manual, by highly trained practitioners, without the flexibility and variability that is likely to occur in real-world practice. Evidence from an efficacy study indicates whether or not an intervention can work in idealised conditions. Typically, efficacy studies control for contextual variation, so do not usually help to illuminate context dependence. An efficacy perspective could be taken to the development, feasibility and evaluation phases of intervention research. However, if found efficacious, this would not provide sufficient evidence for implementation. For example, testing whether or not a vaccine is efficacious in preventing infection under optimal conditions would be undertaken prior to developing a delivery programme, which would then need to be tested for effectiveness in practice. Another example is testing a psychosocial intervention for efficacy in optimal conditions, using experienced practitioners and carefully selected patients, a precursor to a further research cycle to refine and test the intervention for effectiveness in real-world settings.

### Effectiveness perspective

Research from an effectiveness perspective seeks to answer pragmatic questions about the effects that are produced by interventions in routine practice to directly inform the choice between intervention options.^38^ Effectiveness studies aim to test an intervention in samples and settings representative of those in which the intervention would be implemented in everyday practice, usually with a health or health service outcome. Flexibility in intervention delivery and adherence may be permitted to allow for variation in how, where and by whom interventions are delivered and received. Standardisation of interventions may relate more to the underlying process and functions of the intervention than on the specific form of components delivered.^40^ For example, the INCLUSIVE trial^41^ assessed the effectiveness of the Learning Together programme of restorative justice to reduce bullying and aggression in schools. Although key intervention inputs were provided to all intervention schools with the aim that key functions were delivered to trigger the theorised mechanisms of change, each school was encouraged to ensure that the form of local implementation was appropriate for their students and context, with scope for locally decided actions.^41^

### Theory-based perspective

The primary aim of adopting a theory-based perspective is to provide evidence on the processes through which interventions lead to change in outcomes and what prerequisites may be required for this change to take place, thus exploring how and why they bring about change. This differs from developing or evaluating interventions using an effectiveness perspective, which focuses on identifying whether or not they ‘work’ based primarily on average estimates of effect. It also differs from an efficacy perspective, as theory-based perspectives explore interventions in practice, taking account of context, and often explore more than one single theoretical account of how the intervention may work. Such approaches to evaluation aim to broaden the scope of the evaluation to understand how an intervention works and how this may vary across different contexts or for different individuals.^42^ In research taking a theory-based perspective, interventions are developed and evaluated through a continuous process of developing, testing and updating programme theory. Research from this perspective can generate an understanding of how mechanisms and context interact, providing evidence that can be applied in other contexts. For example, there are numerous mechanisms by which group-based weight loss interventions may bring about behaviour change. Change may be motivated by participants’ relationship with the facilitator or by interaction and sharing of experiences among members of the group, as well as by the specific content of the intervention. Whether or not and how such mechanisms generate outcomes will depend on the context in which the intervention is being applied.^43^ Thus, the impacts of interventions cannot confidently be determined in the absence of knowledge of the context in which they have been implemented.^44^

There are several approaches that take a theory-based perspective (Box 3 shows a selection of examples). They are ‘methods neutral’ in the sense that they draw on both quantitative and qualitative study designs to test and refine programme theories.

### Systems perspective

A systems perspective suggests that interventions can be better understood with an examination of the system(s) in which they are embedded or the systems that they set out to change. A systems perspective treats interventions as events within, or disruptions to, systems.^24^ The properties of a system cannot be fully explained by understanding only each of the system’s individual parts.^49^ This perspective is concerned with an awareness and understanding of the whole system:

The essential point is that the theory driving the intervention is about the dynamics of the context or system, not the psyche or attributes of the individuals within it.

*Hawe* et al.^24^

Key to a systems perspective is considering the relationships between intervention and context, engaging with multiple perspectives, and identifying and reflecting on system boundaries.^50^ A systems perspective encourages researchers to consider how the intervention may be influenced by and impact on many elements of the system, and over an extended period of time. Rather than focusing on a narrow and predetermined set of individuals and outcomes within a fixed time period, a systems perspective will aim to consider multiple ways by which an intervention may contribute to system change. These may occur through multiple, often indirect, routes over an extended time period and through spillover and diffusion processes, some of which may be unintended. It is not usually possible to take account of a whole, often open, system; therefore, to make an evaluation tractable it will be necessary to limit its scope by determining a system boundary and restricting the range of potential mechanisms or explanations that are investigated.

### Summary

Interventions rarely achieve effects evenly across individuals, populations and contexts. For complex intervention research to be useful to decision-makers it needs to take into account the complexity that arises both from the intervention’s components and from the intervention’s interaction with context. Intervention effects can be dependent on, can influence or change contexts, and this interdependence can vary over time. Complex intervention research should be conducted with an awareness of these multiple potential sources of complexity, with the design of any specific research study adopting the research perspective that is most suited to the research question that it aims to address (see Box 4 for examples). The preponderance of complex health intervention research to date has taken an efficacy or effectiveness perspective, and there will continue to be many situations in which research questions for which these perspectives are most suited will continue to be prioritised. However, for many interventions, the most critical research questions and the needs of decision-makers who use research evidence are not met by research that is restricted to questions of efficacy and effectiveness; therefore, a wider range of research perspectives and methods needs to be considered and used by researchers, and supported by funders. This may particularly be the case in health service delivery and public health research, in which organisational-level and population-level interventions and outcomes are research priorities that are not well served by traditional methods.^6,7^ Key questions for evaluation include identifying generalisable determinants of beneficial outcomes and assessing how an intervention contributes to reshaping a system in favourable ways, rather than the dominant focus on the binary question of effectiveness in terms of individual-level outcomes. In the next section, we set out the revised framework that aims to help research teams consider the research perspective(s) most suited to the research challenge that they are aiming to address, to prioritise research questions, and choose and implement appropriate methods.

**Key Points 1 Intervention complexity and research perspectives**

- Complexity arises from properties of the intervention, from the context in which it is delivered and from the interaction between the two.
- Consider different research perspectives and how they could be used to conceptualise and evaluate the intervention, its place in the wider system in which it is implemented and the interdependence between the two over time.
- The key to choosing a research perspective is to consider the available approaches and use those that are most helpful, given your research questions.
- Complexity-informed research with an awareness of system(s) can encourage (1) researchers to develop research questions that take into account the wider contextual factors that influence and/or are influenced by an intervention over time, and (2) researchers, funders, practitioners and policy-makers to develop, evaluate and implement interventions using the most appropriate tools and methods.

## Framework for the main phases and core elements of complex intervention research

### Overview

This section presents an overview of the main phases and core elements of complex intervention research.

Figure 3 shows the four main phases of intervention research: development or identification, feasibility, evaluation and implementation. Each phase is connected to a set of core elements considering context, developing and refining programme theory, engaging stakeholders, identifying key uncertainties, refining the intervention and economic considerations. We recommend that these elements are revisited continually throughout the research process and especially before moving between phases (e.g. between feasibility assessment and evaluation). It is vital that stakeholder input, contextual considerations and economic considerations are included early and continually throughout the research process, given that there is little point in going through the other phases if ultimately the intervention is unlikely to ever be implementable in real-world practice. *Case study 1* provides an example of a complex intervention throughout its journey from development to implementation and its adaptation to different contexts.

A complex intervention research programme may begin at any phase, depending on what the key uncertainties are about the intervention in question. The research may involve numerous iterative stages. Repeating of phases is preferable to automatic progression if uncertainties remain unresolved. As with drug development research, for which only a tiny minority of promising drug discoveries make it through to implementation,^63^ it is likely that many new intervention ideas will prove not to be worthwhile. If the feasibility phase shows that the initial intervention idea is not deliverable or acceptable, a decision is needed on whether to stop the research altogether or to do further development work, rather than press ahead with an effectiveness study. In the case of policies or programmes, for which stopping the intervention is not within the researchers’ control, an evaluability assessment (see *Evaluability assessment*) may show that the intervention cannot be usefully evaluated in its current form and that further development is needed before an informative evaluation can be undertaken.

**Figure 3:** Main phases and core elements of complex intervention research. The diagram illustrates the non-linear, iterative pathway through the research process. The core elements should be repeatedly considered and may lead to further work at the same phase or movement to another phase. Reproduced with permission from Skivington *et al*.^26^ This is an Open Access article distributed in accordance with the terms of the Creative Commons Attribution (CC BY 4.0) license, which permits others to distribute, remix, adapt and build upon this work, for commercial use, provided the original work is properly cited. See: https://creativecommons.org/licenses/by/4.0/. The figure includes minor additions and formatting changes to the original figure.

Definitions of terms used in Figure 3:

- Context: any feature of the circumstances in which an intervention is conceived, developed, evaluated and implemented.
- Programme theory: how an intervention is expected to lead to its effects and under what conditions. The programme theory should be tested and refined at all stages and used to guide the identification of uncertainties and research questions.
- Stakeholders: those who are targeted by the intervention or policy, involved in its development or delivery, or more broadly those whose personal or professional interests are affected (i.e. who have a stake in the topic). This includes patients and members of the public, as well as those linked in a professional capacity.
- Refinement: the process of ‘fine tuning’ or making changes to the intervention once a preliminary version (prototype) has been developed.
- Uncertainties: identifying the key uncertainties that exist given what is already known and what the programme theory, research team and stakeholders identify as being most important to find out. These judgements inform the framing of research questions that, in turn, govern the choice of research perspective.
- Economic considerations: what are the comparative resource and outcome consequences of the interventions for those people and organisations affected?

### Core elements

Parts of this text have been reproduced with permission from Skivington *et al*.^26^ This is an Open Access article distributed in accordance with the terms of the Creative Commons Attribution (CC BY 4.0) license, which permits others to distribute, remix, adapt and build upon this work, for commercial use, provided the original work is properly cited. See: https://creativecommons.org/licenses/by/4.0/. The text below includes minor additions and formatting changes to the original text.

We have identified six core elements that require consideration throughout all of the phases of complex intervention research. These are (1) context; (2) development and ongoing iteration of programme theory; (3) involvement of relevant stakeholders; (4) identification of key uncertainties to define and prioritise research questions; (5) ongoing intervention refinement; and (6) economic considerations. This section provides an overview of these core elements, with the following sections linking them to the four research phases (development, feasibility, evaluation and implementation).

#### Context

Context: … any feature of the circumstances in which an intervention is conceived, developed, implemented and evaluated.

*Reproduced with permission from Craig* et al.^10^ *Contains information licensed under the Non-Commercial Government Licence v2.0*

Context is dynamic and multidimensional. Key dimensions include physical, spatial, social, cultural, digital, political or economic features of the circumstances in which interventions are implemented. Context ‘interacts, influences, modifies and facilitates or constrains the intervention’.^64^ How an intervention works may vary across context and what works in one context may not be as effective, or may even be harmful, in different contexts (see *Case study 2*). The impact of a new intervention will depend on what provision already exists. Interventions may need to be explicitly adapted to fit different contexts (Box 5). As well as being influenced by context, many ‘interventions work to change the context within which health is produced and become part of the context for future health behaviours, interventions and outcomes’ (reproduced with permission from Craig *et al*.^10^

Contains information licensed under the Non-Commercial Government Licence v2.0). Theorising interventions as interruptions in systems highlights the dynamic interdependence of intervention and context over time. Taking context into account is crucial throughout the research process:

- in theorising how the intervention will generate effects, intended and unintended
- in anticipating and understanding barriers to and facilitators of the implementation, and scaling up and scaling out of interventions
- in the development phase to make it more likely that the intervention is effective and implementable among the target population and setting
- at the evaluation phase, to understand how interventions ‘work’ in terms of how they interact with their context
- in the reporting of research, to help decision-makers understand whether or not the intervention will work and for whom in the context for which they are responsible.^10^

Circumstances surrounding the intervention may change after the study has begun, for example through the introduction of a new policy or service or, in the case of evaluations with long-term follow-up, more fundamental changes in the social or economic context. The context may also interact with the intervention and interventions may be designed to be adaptive and responsive to context.

It is important to develop a clear understanding of what aspects of context may be important in a broader consideration of intervention outcomes and processes, and which of these are particularly relevant to the research questions to be addressed. Considering these wider contextual factors at the earliest stage can help to ensure that the research improves understanding of how an intervention works and how it might operate in or respond to different circumstances. It allows interventions to be developed, refined and adapted so that they fit with the contexts in which they are implemented.

**Key Points 2 Context**

- The context (see *Context*) in which an intervention is being delivered has a role in its success, or otherwise.
- Theorising an intervention as an event in a system highlights the dynamic interdependence of intervention and context over time.
- Context is multidimensional and it is important to be clear about which aspects of context are likely to influence the delivery, effectiveness, reach and sustainability of the intervention.
- An understanding of the interdependence between context, intervention and outcomes should be developed at the outset and continuously revisited and documented throughout the research process.
- Clear understanding, description and explanation of context enables judgements to be made about transferability of the intervention and better enables intervention adaptation and implementation.

**Signposts to Further Reading 2 Context**

- *Taking Account of Context in Population Health Intervention Research: Guidance for Producers, Users and Funders of Research*.^10^
- Pfadenhauer *et al*.’s^70^ the Context and Implementation of Complex Interventions (CICI) framework.
- A realist conceptualisation of context.^71^
- Viewing intervention context in a more dynamic way through a systems lens provides many challenges to complex intervention research.^62^

#### Programme theory

Programme theory describes how an intervention is expected to lead to a set of specified outcomes and under what conditions. It articulates (1) the key components of the intervention and how they interact; (2) the mechanisms of the intervention; (3) the features of the context that are expected to influence those mechanisms; and (4) how those mechanisms may influence the context.^72^

*Case study 3* provides an example of how programme theory has been developed and used. For interventions designed to be adaptable across contexts, the programme theory should articulate the key functions that must be consistently delivered and indicate how far interventions can be responsive to different and changing contexts.^40^,^73^ A programme theory may include aspects of mid-range or grand theories, but its aim is more specific: to articulate how and why an intervention is expected to work.

##### Developing programme theory

Programme theory can be used to promote a shared understanding of the intervention and how it works among diverse stakeholders involved in intervention implementation and evaluation, as well as among those consuming and using the research. It can also be used to identify key uncertainties and research questions. Programme theory should be ‘adaptive’, continually updated, developed and refined as understanding of the intervention and its interaction with the context and system changes over time. The best practice is to develop programme theory at the beginning of the research project based on the evidence base and theory from relevant fields with involvement of diverse stakeholders, and to refine it during successive stages of the research to produce an updated programme theory at project completion. A refined programme theory is an important evaluation outcome and is the principal aim for which a theory-based perspective is taken. Improved theory will help inform transferability of interventions across settings and help produce evidence and understanding that is useful to decision-makers. It may demonstrate that an intervention is less likely to be effective when transferred to a given context; this in itself is a useful outcome.

Where researchers are involved in evaluating an intervention (such as a policy) developed by others, they still need to theorise the intervention before attempting to evaluate it.^74^ This involves defining the content and purpose of the policy clearly, and articulating and refining a programme theory. As with researcher-developed interventions, involvement of stakeholders is critical. Key documents can also be drawn on (e.g. policy documents or existing funding applications) to refine programme theory. Developing a programme theory is part of completing an evaluability assessment of an existing or proposed policy intervention,^75^ discussed further in *Evaluability assessment*.

For further guidance, see *Signposts to further reading 3: programme theory*.

##### Representing programme theory

Programme theory should always be clearly articulated. It can be helpful to provide a visual representation of aspects of the programme theory. This is particularly useful for shared understanding among stakeholders of the intervention and the potentially multiple ways that it may generate outcomes. It is important to note that any single visual representation will often not be sufficient to fully articulate the programme theory. There are various ways of depicting a programme theory, for example logic models, realist matrices and systems maps. Each method shows something slightly different and is, therefore, useful in different circumstances.

###### Logic models

The terms logic model and programme theory have been used inconsistently in the past, with logic model often being used interchangeably with programme or intervention theory. Given that logic models (a method of visually representing some elements of the programme theory) have varying levels of detail, typically showing the inputs, outputs and intended outcomes but not always considering the intervention mechanisms or context dependencies, we distinguish the terms to highlight the importance of clearly articulating the full programme theory. Nonetheless, logic models can be very useful alongside programme theory to clarify thinking, to gain a shared definition and understanding of the intervention among diverse stakeholders, and to guide and communicate with others about the outcome evaluation. More complex forms of logic model are increasingly used. They include causal logic models that show mechanisms and pre-conditions (see *Case study 4*) and ‘dark logic models’ that depict unintended and potentially negative consequences of the intervention (see *Case study 5*).^76^

###### Realist approaches

Programme theory is at the core of realist evaluation. There are a variety of methods for developing and presenting realist programme theory, such as creating multiple draft theories for different aspects of the intervention to inform the overall programme theory^77^ and the use of realist matrices.^72^ A realist matrix is a table addressing aspects of the intervention, such as agency, context, resources, mechanisms and outcomes (see *Case study* 6). Realist matrices present the hypothesised causal mechanisms of the intervention and attempt to address the question ‘what works in which circumstances and for whom?’.

###### System maps

If taking a systems perspective, it may be appropriate to create a system map that embeds the intervention in the wider dynamic context. Here, the intervention is viewed as an event within a complex system, in which different components of the system and relationships between them will react in various ways, with the potential to affect different outcomes and generate system change. A system map is a conceptual visual map that is created to describe the components of the system and the relationships between them. Group model building and other soft systems methodologies can be used to create system maps using inputs from multiple perspectives to gain a holistic and shared understanding.^78–81^ An initial system map, created with the input of various stakeholders, may help to refine the programme theory and to define the scope of the evaluation. A systems perspective supported by systems mapping will support the inclusion of longer-term, spill-over feedback and potential emergent effects of interventions that would potentially be missed by other research perspectives that tend to focus on more proximal, linear effects. For example, an evaluation of the UK Soft Drinks Industry Levy (SDIL) used system mapping, involving multiple stakeholders, as a starting point for developing programme theory (see *Case study* 7). System maps can also be complementary to the realist approaches described above.^82^

**Key Points 3 Programme theory**

- Programme theory describes how an intervention is expected to lead to a set of specified outcomes, and under what conditions. It should be developed at the outset as a collaborative process involving researchers and stakeholders.
- The development of programme theory is an iterative process. An updated programme theory should be produced at the end of each phase of development and evaluation to capture learning and guide next steps.
- Use the programme theory to choose appropriate evaluation outcomes, including measurement and testing of the theory.
- Visual representation of the programme theory can be useful for summarising programme theory and communicating with stakeholders. However, a comprehensive programme theory should always be clearly articulated and not just represented in a logic model(s) or other simplified visual representation.

**Signposts to Further Reading 3 Programme theory**

- Overview of the various types of theory.^83^
- The use of programme theory within intervention development.^11^
- The use of programme theory for evaluating policy-led processes and outcomes.^74^
- The 6SQuID model for intervention development provides detail on the use of theory.^17^
- Intervention mapping is a theory-led approach to developing interventions and their evaluation.^84^
- Extending logic models with a complexity perspective.^85^
- *What theory, for whom and in which context? Reflections on the application of theory in the development and evaluation of complex population health interventions*.^86^
- *Purposeful Program Theory. Effective Use of Theories of Change and Logic Models*.^72^
- Realist reviews to inform programme theories.^87^

6SQuID, 6 Steps in Quality Intervention Development.

### Stakeholders

Stakeholder engagement is required throughout the research process to maximise the likelihood that the research addresses relevant questions and leads to changes in practice or policy:

**Stakeholders**: Those who are targeted by the intervention or policy, involved in its development or delivery, or more broadly those whose personal or professional interests are affected i.e. who have a stake in the topic. This includes patients and members of the public as well as those linked in a professional capacity.

Stakeholders should be involved early in the research process and remain engaged throughout. Reciprocity is vital, and the goal should be the development of collaborative relationships that continue throughout the phases of development, evaluation and implementation.

The purpose of stakeholder engagement will differ depending on the context and phase of the research, but could include drawing on stakeholders’ practical experience; obtaining stakeholders’ perspectives on the problem being addressed by the intervention; gaining stakeholders’ input to and shared understanding of the programme theory, including understanding of the context and system properties; identifying potential areas for change; choosing appropriate outcomes; and ensuring that all ‘voices’ are heard. It can also help with prioritising research questions, choosing the most useful evaluation perspective, building consensus and fostering relationships to make evaluation more straightforward, for example by accessing gatekeepers, supporting intervention refinement and considering issues around transferability and implementation. Where a systems perspective is taken, stakeholder engagement can help to define the system boundaries and, thus, the scope of the evaluation.

The risks of not engaging relevant stakeholders in complex intervention research include the intervention being theorised or considered from a narrow perspective only; the intervention not being readily implementable or supported by existing system context; the intervention being infeasible or unacceptable; and the chosen outcomes not being important to the patients, public, policy or practice. For example, outcomes of surgical interventions for severe and complex obesity depend on patient engagement with new lifestyle and diet, and work better with multidisciplinary care from psychologists and dieticians in addition to the traditional surgical teams.^88^ The development and evaluation of a surgical intervention like this example, therefore, requires multidisciplinary stakeholder engagement, not least with the patients who may receive the intervention. Broad thinking is needed to identify a diverse range of appropriate stakeholders beyond the usual suspects.

#### Stakeholders from professional groups

Engaging stakeholders from professional groups can be difficult for practical reasons: because they have other priorities or because they have competing or vested interests. For example, social security policy-makers may attach low priority to the health impact of welfare reform because their chief concern is the effects on labour market participation; therefore, engaging them in research to evaluate health outcomes is difficult. There may be drawbacks from aligning research too closely with stakeholder priorities. It is important to elicit their research priorities, but also to consider why they are priorities and what decisions they are likely to influence.

These issues may also apply to researchers, particularly those who have been involved in intervention development and who may have a stake in the success of the intervention.^89^ Studies have shown that early positive results are often not replicated in independent evaluation.^90^ Systematic bias has also been evidenced in drug trials, whereby the products of the company funding the research are shown to be favoured over others.^91^ Thorough consideration of the governance issues, conflicts of interest, risks and benefits is particularly important for research partnerships with commercial organisations. Unhealthy commodity industries have been shown to engage in numerous tactics when engaging with research,^92^ for example to divert attention away from population-level to individual-level interventions, despite the former having greater potential to improve population health.^93^

Researchers need to be aware of these sensitive issues. There should be thorough consideration of the appropriate involvement of identified stakeholders with integrity and transparency. An element of critical (and self-critical) distance is needed without losing the benefits of working effectively with stakeholders (see *Signposts to further reading 4: stakeholders*).

Co-production of interventions with effective collaboration between a group of stakeholders can support the move from interventions being driven in a closed manner by researchers, to interventions being developed in partnership with a range of stakeholders.^94^ As noted above for stakeholder engagement, however, partnership and co-production must be conducted openly and in ways that do not distort research priorities or compromise research integrity.

#### Patient and public involvement

One group of stakeholders that is particularly important to involve in research is the potential recipients of an intervention. PPI in research is defined as ‘research being carried out “with” or “by” members of the public rather than “to”, ”about” or “for” them’ (reproduced with permission from INVOLVE).^95^ As well as collaboratively developing research questions and interventions that are relevant and appropriate to participants, and identifying outcomes that are important to them, appropriate PPI is likely to improve the recruitment and retention in evaluation studies.^96^ In particular, people with lived experience of the condition or issue should be seen as having expertise that others cannot bring to the team.^97^ (*Case study 8* illustrates this ‘patient-as-partner approach’.)

It is critical that PPI is meaningful and robustly conducted, rather than tokenistic.^98^ The type of engagement should also be appropriate to the project, for example to the particular condition or issue of interest, which could be sensitive or stigmatised.^99^ There are numerous challenges to overcome to engage patients and the public effectively in research. They include power imbalances between researchers and patients/public, competing priorities, engagement difficulties and creating effective partnerships. Researchers should consider following a formal framework for engaging, monitoring and evaluating stakeholder involvement (see *Signposts to further reading 4: stakeholders*). To improve PPI, research should also include some evaluation of the engagement process,^100^ which should be published where possible, to enable shared learning.^101^

**Key Points 4 Stakeholders**

- ‘Stakeholder’ is a wide-reaching term for anyone who has a stake in the intervention and/or its evaluation or implementation; therefore, careful consideration at the outset of the relevant stakeholders (for different phases of the research) is important.
- It is particularly important to involve patients and the public in research. Consider following a predefined framework for PPI in your research.
- Stakeholder engagement needs to be genuine rather than to satisfy criteria for funding, publication, etc. and should include the prioritisation and design of the research rather than merely its delivery.
- Involvement of appropriate stakeholders is crucial to each phase of research, and ultimately to the prospects of the intervention being effective and the research leading to changes in policy or practice. At the same time, an element of critical distance between researchers and stakeholders is required, and researchers need to beware of sensitive issues that involving certain stakeholders may invoke. The nature of stakeholder involvement should be transparent, with upfront and agreed terms of references and processes for declaring conflicts of interest.

**Signposts to Further Reading 4 Stakeholders**

- INVOLVE [www.invo.org.uk/resource-centre/resource-for-researchers/ (accessed 1 March 2020)] is a national advisory group funded by NIHR to support active public and patient involvement in NHS, public health and social care research. There is an online resource to support researchers to involve the public in how research is identified, prioritised, designed, conducted and disseminated. INVOLVE’s Values and Principles Framework^102^ aims to support the building of collaborative partnerships in research and to collaboratively produce national standards for public involvement.
- See the National Standards for Public Involvement in Research website for updates.^103^
- NIHR handbook for researchers on PPI in health and social care research.^104^
- Different frameworks exist for supporting PPI in research; for a critical overview see Greenhalgh *et al*.^105^
- Recommendations for effective engagement with stakeholders for research prioritisation, alongside a checklist of practices.^106^
- Reporting of PPI in research: primary research guidelines^107^ and systematic review guidelines.^108^
- Guidance on collaboration with industry:
  ∘ MRC *Industry Charter*.^109^
  ∘ NIHR *Model Industry Collaborative Research Agreement* (mICRA Guidance)^110^ and *MRC Industry Collaboration Agreement* (*MICA*).^111^
  ∘ *MRC UKPRP: Principles for Working with Industry*.^112^
  ∘ The Lambert Toolkit for universities and companies that wish to undertake collaborative research projects.^113^
- Researchers can draw on different tools to engage stakeholders, for example:
  ∘ For systems methodology – *Systems Concepts in Action: A Practitioner’s Toolkit.,^114^* and *Soft Systems Methodology in Action.^115^* Examples of soft systems methodology include rich pictures, context diagrams, multiperspective diagrams, group model building, laddering, concept maps, cognitive maps and causal loop diagrams.^81^
  ∘ The Network for Transdisciplinary Research provides a series of methods and tools for collaboration between experts and stakeholders (e.g. ideas for clarifying expectations and dealing with power issues in the multidisciplinary stakeholder group).^116,117^

### Key uncertainties

A major challenge for complex intervention research is that, at any phase, there are multiple research questions that could be usefully addressed and alternative research perspectives that may seem equally plausible. Key to maximising the efficiency and value of research is to focus on addressing the key uncertainties that exist, taking into account what is already known and what the programme theory, research team and stakeholders identify as being the most important to find out. These judgements inform the framing of research questions that, in turn, govern the choice of research perspective. Even if the fullest possible assessment of an intervention depends on understanding its impact on the system in which it is embedded, that understanding may need to be developed through a series of studies that adopt a range of different perspectives, chosen at each phase to be the most relevant to addressing the key uncertainty at that phase. For example, a common approach that fits well with the linear stages of research that were proposed in the 2000 complex intervention framework,^3^ is to focus on establishing whether or not the intervention ‘works’ by obtaining an unbiased estimate of the average effect. This is undertaken initially through an efficacy perspective that is applied within tightly controlled conditions, and then through effectiveness research that is conducted in more realistic conditions. For interventions where the programme theory suggests that there are homogeneous effects across population subgroups, with a low degree of system and context dependence, efficacy and effectiveness are likely to be the key initial uncertainties, with implementation and transferability being the focus of later stages of research. However, for interventions that are designed to be flexible and adaptable or that interact with contexts and systems in a dynamic and variable way, the key initial uncertainties may relate less to efficacy and more to acceptability and compatibility with the wider systems in which they are embedded, with the assessment of impact on key outcomes taking place at a later evaluation phase.

At any given phase of research there may be more than one key uncertainty, which can be addressed through separate research questions and parallel paths of inquiry. For example, modelling the effectiveness and economic implications of scaling up an intervention may be worthwhile alongside a development or feasibility study, to reduce the risk of developing and testing an intervention that has little real prospect of being implemented in practice (Box 6).

Prioritising research questions and choosing a research perspective will be influenced both through the identification of key uncertainties and through the assessment of the likelihood of achieving an unequivocal answer to that question. There is a risk that research questions, research perspectives and research designs that are favoured by existing guidance and research funding mechanisms are those that have a greater probability of finding a certain answer, even though the question may be of less importance. One of the motivations for updating this framework was to answer calls for a change in research priorities, towards allocating greater effort and funding to research that can have the greatest impact on health-care and population health outcomes. This may require a shift away from, for example, efficacy trials of relatively uncomplicated interventions in tightly controlled conditions, for which research questions are answered with great certainty but for which the translation of this evidence into diverse, real-world settings is often highly problematic,^119^ requiring heroic assumptions about the potential for generalisation and implementation. In many areas of health intervention research, it may be more useful to give higher priority to an evaluation that is sensitive to complexity, from which improvements in understanding and important implications for decision-makers can be identified, albeit with caveats, assumptions and limitations.^7^ Thus, theory-based or systems evaluations of complex population-level interventions and mixed-methods evaluations that emphasise implementation, context and system fit may provide more useful knowledge to address key uncertainties for decision-makers than evaluations that seek to provide unequivocal answers to tightly specified questions of less importance. For example, decision-makers may place greater value on research from a systems perspective that assesses an intervention in terms of its potential to contribute to long-term impacts on multiple outcomes, through multiple and often indirect pathways that will vary over place and time, than on research from an effectiveness perspective that measures short-term effects on a single prespecified outcome.

Identifying key uncertainties, priority research questions and optimal research perspectives will ideally involve the active participation of a multidisciplinary research team and a diversity of stakeholders, and ideally with independent oversight, perhaps from a funders’ steering group. Different stakeholders will have different views on what is the most important to find out. Some may not be in equipoise about the intervention’s effectiveness because they are satisfied with what is already known and may, therefore, be more focused on questions of reach, context dependence and issues of implementation; others may feel that greater certainty of effectiveness is required owing to limitations and potential bias in the existing knowledge base. Choice of outcome may also vary according to stakeholder perspective. For example, a local authority evaluating a suite of policies to increase physical activity may prioritise a natural experiment using routinely collected data on pre- and post-intervention use of leisure centres and green space in localities with and without the policy. Researchers may place higher priority on validated measures of physical activity in exposed and unexposed groups to be convinced of the effect of the intervention. An evaluability assessment would be a useful tool in this case to help identify the most worthwhile design and focus of evaluation (see *Evaluability assessment*).

Key uncertainties should be continually revisited as data accumulate and the programme theory and intervention are updated and refined. Formal methods, such as decision analysis, VOI analysis (see Box 6) or agent-based modelling (ABM) with uncertainty quantification, may be useful in prioritising research questions and optimising research design.^120–122^ However, in many situations this will be largely a deliberative process underpinned by the purpose of the evaluation and the needs of research users. Traditionally, research funders and researchers have prioritised research questions and their associated designs and methods that have a good chance of being answered with a high degree of certainty and are strongly influenced by hierarchies of evidence. For many complex interventions, the needs of research users and the complexity of the evaluation may favour decision-making based on a greater diversity of evidence, thus valuing a wider range of research perspectives and methods, and on ‘balance of probabilities’ rather than ‘evidence beyond reasonable doubt’. By involving a wider range of stakeholders in the research team, a consideration of uncertainty in prioritising the next stage of research may lead to a greater focus on reducing uncertainty around important questions than on answering less important questions with certainty; the findings may be equivocal but will nevertheless be of greater value.

**Key Points 5 Uncertainties**

- Identify key uncertainties that the research aims to address, given what is already known and the intervention programme theory. What are the key uncertainties for decision-makers?
- Formulate and prioritise research questions accordingly.
- Uncertainties will change as evidence accumulates, so should be reviewed at each phase of the research process.
- Engage stakeholders to develop a detailed understanding of where uncertainty lies for those funding, delivering or receiving the intervention.
- Consider both the importance of the uncertainty and the likelihood of reducing that uncertainty when prioritising questions and perspectives.
- Identifying which outcomes are important and/or measurable may help prioritise the focus of research.
- In the case of interventions that are occurring anyway outside the control of the research team, such as changes in policy or health-care delivery, evaluability assessment can support the identification of key uncertainties.

**Signposts to Further Reading 5 Uncertainties**

- Uncertainty and the evaluation of health and social interventions.^118,123^
- Decision-theoretic approaches for appraising evidence.^124^
- Two discussions of the uncertainty of evidence surrounding face masks for the protection of the public during COVID-19.^125,126^

### Intervention refinement

Within each phase of complex intervention research and on transition from one phase to another, there may be good reason, based on the data collected and/or the development of programme theory, to refine or optimise the intervention:

**Refinement**: The process of ‘fine tuning’ or making changes to the intervention once a preliminary version (prototype) has been developed.

*O’Cathain* et al.^11^

Intervention refinement (see *Case study 9*) in the development, feasibility and implementation phases can be relatively straightforward, provided that the changes are consistent with the intervention’s programme theory. Such refinements are covered by the argument that the form of an intervention can be flexible as long as the key functions specified in the programme theory are delivered.^40^ However, where the changes are substantial and imply a significant change to the programme theory, such adaptation may require a more fundamental rethinking of the research process. This more fundamental kind of adaptation is distinct from refinement, in that it refers to an existing intervention being adapted to a new population, setting, target outcome or context, and is discussed in *Developingan intervention*.

Intervention refinement and flexibility are discouraged in an efficacy evaluation, in which standardisation and consistency of intervention delivery are prioritised to maximise internal validity. However, from a systems perspective, adaptability and responsiveness are likely to be desirable features of the intervention and a key focus of the research. The extent to which variation in programme delivery is desirable in an effectiveness or theory-based evaluation will depend on the extent to which a flexible form of delivery is allowed, while preserving the delivery of the intervention’s key functions.^40^

In instances of an ‘identified intervention’ (i.e. one that is driven by policy or practice, rather than researcher led; see *Identifying an intervention*), limits on refinement may be set by the policy or practice context. Refinement of policy- or practice-led interventions will require negotiation with those co-ordinating the intervention and all other relevant stakeholders, but may be valuably informed by evaluation results.

#### Rapid refinement of digital interventions

There are specific challenges that are associated with evaluating digital interventions. These interventions offer the potential for wide reach and high impact at a relatively low cost. However, such interventions may require a rapid route to implementation and frequent refinements to avoid becoming ‘out of date’.^127^ Considerations specific to evaluating digital interventions have been summarised by Murray *et al*.,^128^ who suggest that traditional evaluation methods, such as RCTs, could be combined with a more iterative approach to intervention development and refinement. To allow for multiple versions of web-based or mobile interventions to be deployed simultaneously, Continuous Evaluation of Evolving Behavioural Intervention Technologies (CEEBIT) trials have been developed.^129^

#### Transparent reporting of changes

The level of refinement that is considered acceptable at different points in the research process will differ depending on the programme theory and research perspective. However, if refinements are made to an intervention, they should be reported transparently and with justification.

**Key Points 6 Intervention refinement**

- Refinements to an intervention should retain the key features of the programme theory. Therefore, acceptable boundaries of refinement should be agreed beforehand.
- It benefits all stakeholders for the optimal version of an intervention to be evaluated and/or implemented.
- Ongoing refinement can improve the potential implementability of the intervention.

**Signposts to Further Reading 6 Intervention refinement**

- The INDEX study: guidance on developing complex interventions to improve health and health care.^11^
- *Normalisation process theory: a framework for developing, evaluating and implementing complex interventions* [see also www.normalizationprocess.org/ (accessed on 1 March 2021)].^130^
- *A conceptual framework for adaptive preventive* interventions.^131^
- *Developing and evaluating digital interventions to promote behaviour change in health and health care: recommendations resulting from an international workshop*.^127^
- CEEBIT trials: *Continuous evaluation of evolving behavioural intervention technologies.^129^*

### Economic considerations

**Economic considerations**: What are the comparative resource and outcome consequences of the interventions for those people and organisations affected?

Nearly all complex interventions will be costly to implement and will impose costs on a range of populations or organisations. Economic evaluation, the comparative analysis of alternative courses of action in terms of both costs (resource use) and consequences (outcomes and effects), should be a core component of all phases of intervention research. Early identification of the potential costs and outcomes can help with the setting of research questions and the design of an appropriate economic evaluation (see *Case study 10*). Methods for the economic evaluation of health interventions are well developed.^132^ Here, we concentrate on aspects that are particularly important for complex interventions.

Early engagement of economic expertise will help identify which perspective to adopt to answer questions that matter most to decision-makers.^133^ Recent National Institute for Health and Care Excellence (NICE) guidance^134^ recognises that complex interventions are likely to have costs and outcomes across different sectors, and recommends the adoption of a broad (e.g. societal) rather than a narrow (e.g. health service) perspective. Use of an impact inventory is a good way of making choices of perspective and of which costs and outcomes to include, explicit, transparent and comprehensive.^135^ Another important initial consideration is what economic evaluation framework should be adopted. Again, the NICE guidance^134^ advocates broad cost–consequence analysis (CCA) or cost–benefit analysis (CBA) approaches because narrower approaches, such as cost-effectiveness analysis (CEA) or cost–utility analysis (CUA), are unlikely to reflect the full range of costs and consequences. These approaches and the choice between them are discussed more fully in *Economic considerations*.

Central to all forms of economic evaluation is the identification, measurement and valuation of the resources and outcomes of both the intervention being evaluated and the appropriate comparators. What to include may not be obvious to evaluators given the variety of resources required to deliver a complex intervention and the range of intended and unintended consequences for those directly and indirectly affected.^136^ Engaging stakeholders (organisations and individuals involved in commissioning and delivering the intervention or expected to experience additional costs, savings, benefits or negative impacts) in the identification process is essential. Economic considerations are often very important to decision-makers because many of their decisions concern the optimal allocation of scarce resources. A decision-modelling framework may be useful to guide and synthesise complex intervention research.

This understanding of resources (costs) and outcomes, together with evidence from existing studies, such as economic evaluations of comparable interventions, should be incorporated in a programme theory. Programme theories can be used to identify the cost and outcome variables that should be taken forward into the economic evaluation. Interactions between an intervention and the context in which the intervention is delivered may lead to variation in costs and outcomes, underlining the importance of unpicking how causal pathways might work differently for different groups and incorporating this understanding in an explicit programme theory.^137^ Use of programme theories in economic evaluation is considered further in *Developing an intervention*.

*A* further consideration in designing economic evaluations of complex interventions is the trade-off between equity and efficiency; this is important where differential impacts are likely. The costs and the benefits of complex interventions may vary across groups in the population, such that the most efficient interventions in terms of population health gain may not be the most equitable in terms of reducing (or at least not increasing) health inequalities. Considering who the beneficiaries might be and how costs and impacts might differ between them is a crucial part of planning an economic evaluation of a complex intervention, and methods have been developed for conducting economic evaluation in ways that take account of trade-offs between equity and efficiency.^138,139^

Economic considerations that should be taken into account at particular phases in the process of developing and evaluating a complex intervention are addressed in the relevant sections below. Ways in which economic evaluation of a complex intervention might differ from standard approaches are identified and new methods highlighted.

**Key Points 7 Economic considerations**

- Include economists in project teams from the early stages of complex intervention research to ensure that economic considerations are fully incorporated in all important decisions about intervention and evaluation design.
- Inclusion of economic considerations in the programme theory will help with understanding of the problem and shape the design of future feasibility and evaluation studies.
- Depending on the research perspective, it will often be appropriate in complex intervention research to adopt a broad (e.g. societal) rather than a narrow (e.g. health service) perspective to the scope of economic considerations included.
- Equity–efficiency trade-offs should be considered when the intervention is likely to have different costs and benefits for different groups, or where the case for intervention is driven by health equity concerns.

**Signposts to Further Reading 7 Economic considerations**

- Guidance on the development of programme theories and conceptual modelling for economic aspects of complex intervention development.^140^
- An introduction to the principles of health economics and economic evaluation relevant to the specific challenges of public health and its aims of improving population health.^141^
- Guidance for conducting economic evaluations alongside natural experiments.^142^
- Textbook on *Methods for the Economic Evaluation of Health Care Programmes*.^132^
- NICE methods guidance and reference case for undertaking economic evaluations.^134^
- *Exploring uncertainty in cost-effectiveness analysis*.^121^
- *Using Realist Approaches to Explain the Costs and Cost-effectiveness of Programmes*.^137^
- *Conceptualizing a model: a report of the ISPOR-SMDM modelling good research practices Task Force*-2.^140^

## Phases of research

Parts of this text have been reproduced with permission from Skivington *et al.^26^* This is an Open Access article distributed in accordance with the terms of the Creative Commons Attribution (CC BY 4.0) license, which permits others to distribute, remix, adapt and build upon this work, for commercial use, provided the original work is properly cited. See: https://creativecommons.org/licenses/by/4.0/. The text below includes minor additions and formatting changes to the original text.

The following section outlines four phases of research: (1) developing or identifying the intervention, (2) feasibility testing, (3) evaluation and (4) implementation. As illustrated in *Overview*, the phases may overlap and a research programme may begin at any phase, depending on the key uncertainties about the intervention in question. Each phase is discussed in detail below. After each phase, we present a table that addresses the core elements (see *Core elements*) and relevant issues to consider for that phase, followed by a ‘key points’ box and a final box that provides signposts to further guidance and resources.

### Developing or identifying the intervention

Intervention development: The term ‘development’ is used here for the whole process of designing and planning an intervention from initial conception through to feasibility, pilot or evaluation study.

This phase of the research process was referred to as intervention development in the previous version of this framework.^1^ However, this did not address the evaluation of interventions where researchers are not involved in their development and do not have experimental control of implementation, which is often the case in policy- or practice-led interventions. Intervention identification and intervention development represent two distinct pathways of evidence generation to inform policy or practice, characterised by the two pathways shown in Figure 4.^143^ In both cases, a key requirement in this phase is to develop a programme theory for the intervention, identify key uncertainties and formulate research questions to decide which research phase to move onto next and what further research to prioritise.

#### Developing an intervention

The MRC-/NIHR-funded INDEX study has developed comprehensive guidance on intervention development.^11^ Rather than replicate the INDEX guidance here, we provide an overview of the key steps and encourage readers to access the main INDEX document (see *Signposts to further reading 8:developing or identifying an intervention*).

The INDEX guidance recommends that a robust process of intervention development should involve the 11 steps outlined in Table 3.

In practice, intervention development approaches will need to be tailored to the capacity of the team, context and resources. Some of the steps in Table 3 may overlap or need to be repeated. It may not be possible or necessary to address all of them, but each should be considered for relevance and importance. Teams involved in developing interventions are encouraged to publish their development work so that learning can be shared.

Four additional sets of issues that may need to be addressed are (1) the adaptation of existing interventions to a new context; (2) wider system factors that may need to be taken into account; (3) the consideration of economic factors; and (4) mathematical modelling or simulation of the intervention.

##### Adapting an existing intervention

Although the INDEX guidance focuses on the development of de novo interventions, it may often be the case that the need for a new intervention is responded to through the adaptation of one or more existing interventions. The term adaptation is distinct from that of refinement, in that it refers to one or more existing interventions being adapted to a new population, setting or context.^144^,^145^ Similarly, an intervention found to be effective in improving one particular outcome can be adapted to target other outcomes, for example the A Stop Smoking in Schools Trial (ASSIST) adolescent smoking prevention intervention being adapted to tackle substance misuse and sexual health.^146–148^ A well-developed programme theory can help identify what features of the antecedent intervention(s) need to be changed for the intervention to work in its new application.^144^

**Figure 4:** Two pathways of evidence generation. Reproduced with permission from Ogilvie *et al*.^143^ This is an Open Access article distributed in accordance with the terms of the Creative Commons Attribution (CC BY 4.0) license, which permits others to distribute, remix, adapt and build upon this work, for commercial use, provided the original work is properly cited. See: http://creativecommons.org/licenses/by/4.0/. The figure includes minor additions and formatting changes to the original figure.

**Table 3:** Framework of actions for intervention development

Action	Consider the relevance and importance of the following:
1. Plan the development process	Identify the problem to be targeted and refine the understanding of it throughout the process Assess whether or not the problem is a priority Consider which aspects of the problem are amenable to change Ask whether or not a new intervention is really needed and if the potential benefit of the new intervention justifies the cost of development (and consider the likely short- and long-term cost-effectiveness) Determine the time needed to undertake intervention development Obtain sufficient resources/funding for the intervention development study Draw on one or more of the many published intervention development approaches, recognising that there is no evidence about which approach is best, and apply flexibly depending on the problem and context Involve stakeholders during the planning process (see next table row) Produce a protocol detailing the processes to be undertaken to develop the intervention
2. Involve stakeholders, including those who will deliver, use and benefit from the intervention	Work closely with relevant stakeholders throughout the development process: patients, the public, the target population, service providers, those who pay for health and social services or interventions, policy-makers and intervention design specialists Develop a plan at the start of the process to integrate public and patient involvement into the intervention development process Identify the best ways of working with each type of stakeholder from consultation to co-production, acknowledging that different ways may be relevant for different stakeholders at different times Use creative activities within team meetings to work with stakeholders to understand the problem and generate ideas for the intervention
3. Bring together a team and establish decision-making processes	Include within the development team individuals with relevant expertise: in the problem to be addressed by the intervention include those with personal experience of the problem, in behaviour change when the intervention aims to change behaviour, in maximising engagement of stakeholders and with a strong track record in designing complex interventions It may be hard to make final decisions about the content, format and delivery of the intervention, so only some team members may do this. There is no consensus about the size or constituency of the team that makes these final decisions, but it is important early on to agree a process for making decisions within the team
4. Review published research evidence	Review published research evidence before starting to develop the intervention and throughout the development process (e.g. to identify existing interventions and to understand the evidence base for each proposed substantive intervention component) Look for, and take into account, evidence that the proposed intervention may not work in the way intended
5. Draw on existing theories	Identify an existing theory or framework of theories to inform the intervention at the start of the process (e.g. behaviour change or implementation theory) Where relevant, draw on more than one existing theory or framework of theories (e.g. both psychological and organisational theories)
6. Articulate programme theory	Develop a programme theory. The programme theory may draw on existing theories. Aspects of the programme theory can be represented by a logic model or set of models. Programme theory can also help the development of economic research questions developed in parallel with the intervention Test and refine the programme theory throughout the development process
7. Undertake primary data collection	Use a wide range of research methods throughout (e.g. qualitative research to understand the context in which the intervention will operate and quantitative methods to measure change in intermediate outcomes)
8. Understand context	Understand the context in which the intervention will be implemented. Context may include population and individuals; physical location or geographical setting; social, economic, cultural and political influences; and factors affecting implementation (e.g. organisation, funding and policy)
9. Pay attention to future implementation of the intervention in the real world	From the start, understand facilitators of and barriers to reaching the relevant population, future use of the intervention, and ‘scale up’ and sustainability in real-world contexts
10. Design and refine the intervention	Generate ideas about content, format and delivery with stakeholders Once an early version or prototype of the intervention is available, refine or optimise it using a series of iterations. Each iteration includes an assessment of how acceptable, feasible and engaging the intervention is, including potential harms and unintended consequences, resulting in refinements to the intervention. Repeat the process until uncertainties are resolved Check that the proposed mechanisms of action are supported by early testing
11. End the development phase	There are no established criteria for stopping the intensive development phase and moving on to the feasibility/pilot or evaluation phases. The concepts of data saturation and information power may be useful when assessment of later iterations of the intervention produces few changes Describe the intervention to facilitate transferability of an intervention outside the original team and location in which it was developed Write up the intervention development process so that judgements can be made about the quality of the process, links can be made in the future between intervention development processes and the subsequent success of interventions, and others can learn how it can be done

Reproduced with permission from O’Cathain *et al*.^11^ This is an open access article distributed in accordance with the Creative Commons Attribution 4.0 (CC BY 4.0) license, which permits others to copy, redistribute, remix, transform and build upon this work for any purpose, provided the original work is properly cited, a link to the licence is given, and indication of whether changes were made. See: https://creativecommons.org/licenses/by/4.0/. The table includes minor additions and formatting changes to the original table.

##### Consider the wider system

Acknowledging that an intervention takes place within a wider system guides the intervention development team to consider the ways in which the intervention and system influence one another, for example via feedback loops and adaptivity (see Table 2). Mapping the system or parts of the system can help facilitate this process. See *Case study 11* for an example of how system mapping has been used to support intervention development.

##### Economic considerations

Consideration of economic aspects at the development phase can help frame the research questions that will underpin the design of an economic evaluation. The review undertaken to inform intervention development (see Table 3) should include economic evidence to help decide what economic issues need to be addressed and how economic ideas, such as insights from behavioural economics,^149^ can contribute to the programme theory.

##### Modelling

The development and justification for an intervention, the potential for an existing intervention to be adapted to a different context and/or the identification of uncertainties to be addressed in its evaluation can often be usefully supported by a mathematical model or simulation of the intervention in silico. This may be particularly useful for upstream interventions and policies, or where a systems perspective is being taken to the research. A range of methods can be used (discussed further in *Types of evaluation design and methods*), including deterministic models, microsimulation models (e.g. to assess tobacco control policy^150^) or agent-based models (e.g. to assess the impact of a potential educational campaign on fruit and vegetable consumption^151^). Assessing and/or further refining these models as data accumulate through all research phases may be an important focus of evaluation activities.

For the economic evaluation, the programme theory needs to be translated into a credible conceptual and mathematical structure^152,153^ so that an early-stage decision-analytic model can be formulated that compares the intervention with one or more relevant comparators. This will help to establish the potential cost-effectiveness of the planned intervention and highlight areas of uncertainty that should be explored. VOI analyses can then be undertaken to estimate a monetary value on further research activities, based on the expected gain from reducing uncertainty surrounding the current cost-effectiveness decision.^154,155^ Expected value of perfect parameter information can also be usefully undertaken to identify parameters that drive the uncertainty in the economic model. The evaluation can then focus on those parameters with the most uncertainty.

#### Identifying an intervention

Often, interventions are policy-driven and/or designed in clinical practice. In such cases, researchers are not usually involved in intervention development, but evidence that they have generated may have informed this process. In some instances, implementation of policy- or practice-led intervention may have taken place without a prior evaluation, in which case researchers may need to plan a retrospective evaluation.

Even if researchers are not involved in the design phase, they should still consider the six core elements (Table 4). In particular, they should develop a programme theory and use this to identify key uncertainties. Some policy interventions are based on explicit theory, but many are not. In these cases, identifying the implicit theoretical basis of an intervention is essential to working out how it might be evaluated. This is an important step, even if the programme theory is developed after the intervention has been rolled out. This helps identify mechanisms, important contextual factors and relevant outcome measures.

**Table 4:** Core elements for developing or identifying the intervention

Core elements	Issues to consider	Risk of not considering them
Context	What are the immediate contextual influences on the intervention? What is the environment in which the evaluation will take place? Where is the system boundary?	Lack of understanding of context may result in an intervention that works in one setting, but may be ineffective, or even harmful, when delivered elsewhere
Programme theory	What is the underlying theory or combination of theories? What are the proposed mechanisms of change? What are the proposed short-, medium- and long-term outcomes? What are the wider impacts of the intervention on the system? Explore the current evidence base, in particular, for information to suggest that the proposed intervention may not work as intended Consider context (see row above) The programme theory process is an important step in identifying priority research questions and helping choose an evaluation perspective	An intervention that has little chance of working even in idealised settings An intervention that is unlikely to be sensitive to different contexts ‘in the real world’ Failure to measure impacts of the intervention that might be important Unable to advance theory
Stakeholders	Include relevant stakeholders in the development process, in particular for developing theory of the problem; considering system and context; developing programme theory; providing feedback to help refine and improve the intervention; and identifying potential issues with future implementation Choose different approaches to engage different stakeholders (e.g. evaluability assessment, interviews/focus groups and soft system methodologies)	Resulting intervention that fails to achieve its proposed effect Effective intervention that fails to be adopted and implemented Resulting evaluation that fails to generate useful data on the processes, mechanisms and/or outcomes of the intervention Reduced chance of impact on policy and practice
Uncertainty	Where are the key areas of uncertainty to be addressed in any evaluation of this intervention? What are the priority research questions and optimal research perspective to address them? What is the proposed research design to address these questions and what is the next stage of research? Is the intervention, the programme theory and the proposed evaluation design sufficiently developed for the next stage of the research to be undertaken?	Failure to agree and optimise the research perspective and design Embark prematurely on underspecified research that fails to address the key questions of interest
Intervention refinement	What aspects of the intervention could be improved prior to delivery?	Delivery of a suboptimal intervention and/or evaluation Failure to effectively gather meaningful data on potential mechanisms and outcomes of the intervention Waste of resources
Economic considerations	What is the existing economic evidence? What is the appropriate economic question: taking account of the system and programme theory/economic conceptual model developed? What perspective(s) is/are appropriate? What type of economic evaluation is most appropriate to address the research question? Should spill-over impacts be included?	Remaining uncertainty not identified and/or studies duplicate existing evidence Inappropriate question with limited focus Economic results that underestimate/ overestimate potential cost-effectiveness Study does not answer questions of interest Study does not capture the full range of costs and outcomes

**Key Points 8 Developing or identifying an intervention**

- Specific considerations for intervention development:
  ∘ plan the development process
  ∘ involve stakeholders, including PPI
  ∘ bring together a team and establish decision-making processes
  ∘ review published research evidence
  ∘ draw on existing theories
  ∘ articulate programme theory
  ∘ undertake primary data collection
  ∘ understand context
  ∘ pay attention to future implementation of the intervention in the real world
  ∘ design and refine the intervention
  ∘ consider the wider system within which the intervention operates
  ∘ consider the future evaluation design (that may affect some of the things that you do in the intervention development phase).
- Specific considerations for intervention identification, in addition to the relevant key points above:
  ∘ identify your starting point within the research process most applicable to the current status of the intervention (i.e. evaluating an existing intervention)
  ∘ consider which natural experimental methods could be used to evaluate the intervention
  ∘ develop and refine programme theory if one does not exist.

**Signposts to Further Reading 8 Developing or identifying an intervention**

- The INDEX study: guidance on developing complex interventions to improve health and health care^156^ and the associated reporting guidance GUIDED.^157^
- *Six steps in quality intervention development (6SQuID*).^17^
- Intervention mapping is a theory-led approach to developing interventions and their evaluation.^84^
- *Normalisation process theory: a framework for developing, evaluating and implementing complex interventions*.^130^
- Evaluating the public health impact of health promotion interventions: the RE-AIM framework.^158^
- *Taking Account of Context in Population Health Intervention Research: Guidance for Producers, Users and Funders of Research*.^10^
- *Natural experiments: an overview of methods, approaches, and contributions to public health intervention research*.^159^
- Using natural experimental studies to guide public health action.^143^
- Guidance on conducting economic evaluations alongside natural experiments.^142^
- Economic evaluation of public health interventions.^141^
- Reporting of behaviour change techniques.^160^
- A checklist has been produced for intervention description and replication (TIDieR)^161^ and has been extended to population health and policy interventions (TIDieR Population Health and Policy).^162^

TIDieR, Template for Intervention Description and Replication.

### Feasibility

We follow the Consolidated Standards of Reporting Trials (CONSORT) extension for pilot and feasibility trials,^163^ and use the term feasibility to cover both the feasibility of the intervention and the feasibility of the evaluation design. A feasibility study asks whether or not something can be undertaken, should we proceed with it and, if so, how:

Feasibility studies: Pieces of research done before a main study in order to answer the question ‘Can this study be done?’

*Eldridge* et al.^163^

#### Why is a feasibility phase needed?

The feasibility phase explores the feasibility of the intervention and/or the evaluation design to underpin decisions about whether or not and how to progress to evaluation. Although feasibility studies are most commonly undertaken prior to effectiveness evaluation, they can also be necessary prior to an efficacy, a theory-based or a systems perspective evaluation study. This vital preparatory work is sometimes overlooked or rushed,^163^ which leads to evaluation studies that are undermined by problems related to either the intervention or the evaluation design that could have been identified and overcome through appropriate feasibility research. On the other hand, feasibility studies can sometimes be overspecified and take more time and resources than are necessary to address the key uncertainties with the planned intervention or evaluation design.

A feasibility study is designed to explore the uncertainties that have been identified at the development phase (see *Case study 12*). The aim is then to address these uncertainties to ascertain whether or not it is appropriate to move onto the evaluation phase and optimise study design.

#### Progression criteria

A feasibility study should be designed to assess progression criteria that relate to the feasibility and acceptability of the intervention and/or the evaluation design. Progression criteria should ideally be developed with input from an independent steering committee and relevant stakeholders, and be agreed and signed off prior to the collection of data.

Progression criteria should be used to guide the decision on whether to proceed to the next stage of evaluation or to undertake further feasibility work, or to return to the development phase and/or terminate the research. Assessment of progression criteria may require both qualitative and quantitative data. Examples include (1) qualitative data, such as the acceptability of the intervention content and mode of delivery for participants and providers, and (2) quantitative data, such as recruitment and retention rates. See the HelpMeDoIt! study for an example of progression criteria.^164^

Progression criteria should be worded in a way that provides scope for considered discussion among the study team and stakeholders, ideally with an independent steering committee making the final recommendation. The use of a traffic light system for each progression criterion is a useful approach (green = go, where there are no issues identified; red = stop, where issues identified cannot be resolved; and amber = amend, where issues identified can potentially be resolved).

In many cases, the key uncertainties to be addressed prior to the evaluation phase relate solely to aspects of evaluation design. This may be where the development phase has involved substantial empirical assessment of the intervention, where the intervention is not in the control of the researcher and so cannot be changed, or where the intervention has already been tested for efficacy. In these cases, questions of feasibility may relate to evaluation design only, given that there may be no reason to expect that further changes will need to be made to the intervention to successfully conduct the evaluation. If this is the case, feasibility work may take the form of a pilot study: a small-scale version of some or all of the future evaluation study. To expedite the research process, it may be advantageous to commission a full evaluation in which an initial pilot stage is included, rather than a separate feasibility phase incorporating a pilot study. All being well, the pilot stage could then be continued and expanded into a full evaluation, eliminating a substantial delay between the pilot and the evaluation. If there were problems that needed to be addressed in the pilot, such as problems with recruitment, retention or measurement of study participants, the pilot would be stopped and the research design or methods reviewed.

#### Feasibility of the evaluation design

In terms of evaluation design, a feasibility study should assess key aspects of research design and conduct, which typically include:

- recruitment – time frame, willingness of participants to be randomised, capacity of those involved (e.g. clinicians) to recruit participants and effective strategies
- retention – follow-up rates and reasons for attrition
- sample size – capacity to achieve an appropriate sample size
- outcomes – choice of outcome measures, data collection tools and processes (e.g. missing data on questionnaires or accessibility of routine clinical data), and duration of follow-ups
- analysis – capacity to collect and analyse data within a time frame, and floor or ceiling effects
- unintended outcomes – for example, potential harms or the impact of the intervention on inequalities.

#### Feasibility of the intervention design

Feasibility studies provide an important opportunity to implement the intervention on a small scale and assess any uncertainties regarding the intervention prior to embarking on a full-scale evaluation. Where prior stages of research have not tested the implementation of the intervention in the same kind of circumstances that will exist in the evaluation study, the feasibility phase is the first and an important opportunity to address questions related to optimal intervention content and mode of delivery; the acceptability of the intervention to participants and providers; adherence; the likelihood of cost-effectiveness; and the capacity of providers to deliver the intervention and fit within the setting. Where the intervention programme theory suggests that contextual or implementation factors may influence the effectiveness of the intervention, these questions should be addressed at the feasibility phase, even if the intervention has already been tested for efficacy.

Many intervention-related questions that might be assessed in a feasibility study relate to process evaluation, which aims to understand the functioning of an intervention by examining implementation, mechanisms of impact and contextual factors.^5,165^

Depending on the results of the feasibility study, further work may be required to progressively refine the intervention before embarking on a full-scale evaluation. As discussed in *Intervention refinement*, any refinement should have agreed limits and be reported transparently. Ideally, incremental refinements should each be tested separately; however, in practice refinements can be made simultaneously if sufficiently rich data are collected to enable judgements about which refinements are helpful and which are not.

The programme theory should be refined in an ongoing way regularly throughout the feasibility study and indeed all phases of the research process.

#### Efficacy signals

To justify the expense of a full-scale evaluation from an effectiveness, theory of change or systems perspective, as well as evidence of feasibility it is also helpful to have convincing empirical or theoretical evidence to suggest that the intervention has the potential to be effective. Such evidence may come from prior research conducted from an efficacy perspective that has demonstrated that the intervention can work in ideal, controlled conditions. Such ‘efficacy signals’ or evidence of potential effectiveness may also be drawn from evidence of the effectiveness of similar interventions for which the programme theory suggests that the effects may be transferable to different populations, settings or outcomes. Feasibility studies should generally not aim to identify efficacy signals or estimate effect sizes for use in sample size calculations, given that these will be very imprecise. However, in some cases, a feasibility study may provide an opportunity to collect data to support the case for potential effectiveness, for example evidence of short-term impacts on proximal, sensitive and intermediate outcomes.

#### Economic considerations for the feasibility phase

The aim of an economic evaluation conducted within a feasibility study is to explore the likelihood that the intervention will be worthwhile, identify key areas of uncertainty and understand whether or not the data required for a full economic evaluation can be captured. Economic considerations that should be addressed during the feasibility phase include refinement of the programme theory that will underpin the full economic evaluation, development and testing of primary data collection tools, and identification of relevant sources of routinely collected resource use and outcome data. Economic modelling can be undertaken to assess the likelihood that the expected benefits of the intervention justify the costs and to help decision-makers decide whether or not it is worthwhile to proceed to a full-scale evaluation. A VOI study (see *Developing an intervention*) should be considered if one was not included in the intervention development phase. If a VOI study was included then it should be updated at this stage. It should be noted that such analyses may increase rather than reduce uncertainty by identifying new sources of variation in costs and outcomes. This can be helpful when a policy-maker believes that an intervention is cost-effective, despite a lack of evidence.

*Case study 10* demonstrates how economic considerations at the design and feasibility phases were used to build the case for a full-scale trial of a complex intervention.

#### Evaluability assessment

An alternative or complementary approach to feasibility is evaluability assessment, a systematic, collaborative approach that is often used with policy-driven or practice-driven (identified) interventions that can equally be used to plan the development and evaluation of a researcher-developed intervention. It aims to determine whether or not an intervention can usefully be evaluated and, if so, which methods are the most appropriate. Its purpose is to ensure that evaluation resources are used efficiently and to minimise research waste on evaluations of poorly designed and/or implemented interventions, or those for which the available data or the method of implementation do not permit outcome evaluation. Evaluability assessment involves collaboration with stakeholders to reach agreement on what are the expected outcomes of the intervention, what data could be collected to assess processes and outcomes and what are the options for designing the evaluation.^75^ The end result is a recommendation on whether or not an evaluation is feasible, should be undertaken and can be carried out at a reasonable cost, and by which methods this can be undertaken.^166^ Five questions to assess evaluability have been suggested by Ogilvie *et al*.:^166^

1. Where is a particular intervention situated in the evolutionary flow chart of an overall intervention programme?
2. How will an evaluative study of this intervention affect policy decisions?
3. What are the plausible sizes and distribution of the intervention’s hypothesised impacts?
4. How will the findings of an evaluative study add value to the existing scientific evidence?
5. Is it practicable to evaluate the intervention in the time available?

*Case study 13* provides an example of using evaluability assessment of existing community interventions to identify practice-based evidence.

**Table 5:** Core elements for the feasibility phase

Core elements	Issues to consider	Risk of not considering them
Context	Determine the contextual dependencies for the intervention (e.g. factors affecting delivery and receipt) and evaluation (e.g. factors affecting collection of outcome data) Consider how these contextual dependencies may change for a future evaluation or implementation within different settings Incorporate aspects of process evaluation	An intervention and/or evaluation that does not demonstrate feasibility and/or acceptability, with no understanding of why An intervention and/or evaluation that fails to meet progression criteria
Programme theory	Begin with a proposed programme theory Use programme theory to identify measurable outcomes Refine the programme theory as data are gathered to inform how the intervention operates Incorporate aspects of process evaluation to explore potential mechanisms of impact Produce a revised programme theory by the end of the phase	Key contextual dependencies are neglected resulting in an intervention that fails to operate as designed
Stakeholders	Input is critical to ensure that relevant data are collected to inform future delivery, evaluation, implementation and impact Input is critical to programme theory and key uncertainties	Lack of useful and convincing data to inform progression to the next phase of evaluation and/or implementation Planning and progression to the next phase will not meet stakeholders’needs or benefit from their critical insights Risk of proceeding with a full-scale definitive trial for an intervention that is highly unlikely to be implemented in practice
Uncertainty	What are the key questions that need to be addressed to progress to an evaluation study? Translate these into progression criteria and use these progression criteria as the principal questions to be addressed in the feasibility study Prior to evaluation, the feasibility study may assess the acceptability of intervention and evaluation to participants, providers and the research setting Consider undertaking an evaluability assessment to determine whether or not and how an evaluation should be undertaken	Progression to a large-scale, potentially expensive evaluation study that then fails because the evaluation design and methods and/or the intervention design and implementation have not been sufficiently tested for feasibility for acceptability Contextual and implementation factors undermine the intervention in ways that were not experienced or anticipated in prior development or efficacy research
Intervention refinement	Agree boundaries and limits on how much the intervention or evaluation design can be refined Report all refinement transparently	Risk of proceeding to the next phase of evaluation with a suboptimal intervention and/or evaluation design
Economic considerations	Identify and measure data on resource use across a range of cost categories consistent with the perspective of the study Identify relevant unit cost sources for the valuation of resource use Consider logistical issues with collecting resource use data (e.g. practical and logical) Develop economic data collection tools and systems. Test the acceptability of data collection tools Consider whether or not qualitative and process evaluation can feed valuable information into the economic evaluation alongside the feasibility study or economic model Identify and measure suitable economic outcomes (preferably preference based) Consider which economic evaluation frameworks are suited to the research question (possibly consider using more than one framework) Consider the use of VOI: potentially useful for identifying areas of particular uncertainty and further research, and identifying a monetary value limit for the costs of any further research activities	Underestimation of time and resources required to collect resource use and outcome data: could ultimately lead to ‘failure’of the trial or ‘ineffective intervention’owing to poor data collection Data collection tools or systems that are unacceptable to patients/clients or those delivering the intervention Inappropriately designed economic evaluation for a definitive trial (e.g. full range of resources and outcomes not correctly identified and measured) Limited economic evaluation frameworks considered Expense of proceeding with a full-scale definitive trial for an intervention that is highly unlikely to be cost-effective or implementable in practice

**Key Points 9 Feasibility**

- Assess the feasibility of the intervention design and/or the evaluation design.
- Use qualitative and quantitative methods to explore and address uncertainties.
- Involve stakeholders to ensure that relevant data are collected for future decision-making.
- If needed, refine the intervention and/or evaluation design (within agreed boundaries) and report changes transparently.
- Use predefined progression criteria to guide decisions about proceeding to evaluation.
- Identify and measure key resource use and outcomes for the purposes of designing a full economic evaluation.
- Consider whether or not it is appropriate and relevant to undertake early economic modelling and VOI analyses.
- Consider conducting an evaluability assessment to determine whether or not and how a full-scale evaluation should be undertaken.

**Signposts to Further Reading 9 Feasibility**

- More detailed guidance on feasibility studies is in preparation by the MRC/NIHR GUEST project team.^167,168^
- *Taking Account of Context in Population Health Intervention Research: Guidance for Producers, Users and Funders of Research*.^10^
- *Process Evaluation of Complex Interventions: Medical Research Council Guidance*.^5^
- *Applied Methods of Cost-Benefit Analysis in Health Care*.^169^
- *Consolidated Health Economic Evaluation Reporting Standards (CHEERS) statement*.^170^
- *Good research practices for cost-effectiveness analysis alongside clinical trials: the ISPOR RCT-CEA Task Force report*.^171^
- *Evaluability Assessment: A Systematic Approach to Deciding Whether and How to Evaluate Programmes and Policies. Working Paper.^75^*
- *Assessing the evaluability of complex public health interventions: five questions for researchers, funders, and policymakers*.^166^

### Evaluation

**Evaluation**: … enables judgments to be made about the value of an intervention. It includes whether an intervention ‘works’ in the sense of achieving its intended outcome identifying what other impacts it has, theorising how it works, taking account of how it interacts with the context in which it is implemented, how it contributes to system change, and how the evidence can be used to support real-world decision-making.

In *Purpose of evaluation*, we discuss the purpose of evaluation and suggest that the primary focus should be providing useful evidence rather than minimising bias. *Research perspectives and evaluation* considers how research perspectives should inform design choices. The remaining sections consider choice of outcomes (see *Choice of evaluation outcomes*), provide information on selection of evaluation designs (see *Types of evaluation design and methods*), consider understanding processes (see *Understanding processes*), consider economic considerations (see *Economic considerations*) and consider good practice in reporting evaluations (see *Reporting evaluations*).

#### Purpose of evaluation

A key change in the field of evaluation has been a shift towards assessing the ‘usefulness’ of information for decision-making, in contrast to focusing exclusively on obtaining unbiased estimates of effectiveness.^172^ In a trial, randomisation, coupled with other elements of trial design (such as concealment of the allocation sequence and blinding of participants and researchers), can minimise bias. A statistical model of the process that determines exposure to the intervention serves the same purpose in a natural experimental study (see *Natural experimental designs*); however, this may not be as effective as randomisation at creating balanced (or ‘exchangeable’) intervention and control groups. However, to maximise the usefulness of the evidence for decision-making, other elements are required, such as the use of programme theory and consultation with stakeholders to identify research questions that matter and a process evaluation to assess elements of implementation, context and mechanisms of impact.^5^ We emphasise that here we are advocating for additions to the toolkit of methods, rather than replacements for traditional methods, when they are appropriate. A more fundamental challenge to the conventional idea that precise and unbiased estimates of effectiveness are the principal goal of evaluation is the proposal that improving theories about how to intervene should be the ambition. In this view, effect estimates are inherently context bound, so that average effects are not a useful guide to decision-makers working in a particular context. More useful may be contextualised understandings of how an intervention works and details on the most important enablers and constraints on its delivery across a range of settings.

Often, questions about the delivery, reach and maintenance of interventions are assigned to ‘implementation research’ and are explored separately from evaluation questions. Some frameworks, such as RE-AIM,^158^ try to pull them together. A key recommendation here is that implementation questions should be considered alongside evaluation questions from the outset. Moving away from a narrow ‘effectiveness’ focus should increase the relevance of the evidence produced by evaluation studies and increase the speed with which interventions that make a useful contribution can be implemented within policy and practice. This is about explicitly considering from the outset how the research will make a difference, who will use it and who could benefit from it. For example, who needs to know the answer to the research questions? One component of a research proposal should be a ‘Pathways to Impact’ statement or an implementation plan; these are often required by funders,^173^ but even if not it is a good idea to have given it due consideration.

#### Research perspectives and evaluation

Table 6 sets out the main research perspectives used in evaluation and relates them to study designs: (1) efficacy, (2) effectiveness, (3) theory based and (4) systems. Although some designs and perspectives tend to be closely associated with one another, the mapping is not fixed and designs can be combined to answer a range of research questions about any given intervention. Efficacy and effectiveness evaluations do not represent a dichotomy but a continuum,^39^ and it is ‘likely impossible to perform a pure efficacy study or pure effectiveness study’.^189^ An effectiveness evaluation can draw on a theory-based perspective to help to explore the impact of context and mechanisms on the outcomes being assessed. For example, a theory-informed process evaluation was conducted alongside an effectiveness trial of the 3D intervention^190^ (to improve the management of multimorbidity in primary care, see *Case study 14*) to understand how and why the intervention was effective or not, as well as to understand the effect of context. An effectiveness evaluation can also draw on a systems perspective to help determine which outcomes should be evaluated. For example, in determining an evaluation strategy to explore the impacts of the SDIL, researchers drew on a systems perspective and methods (see *Case study 7*).^191^ Theory-based and systems perspectives to evaluation can also be used in conjunction; for example, methods developed using systems thinking could be used to support and focus the involvement of stakeholders in a realist evaluation.^82^

**Table 6:** Research perspectives and evaluation

Perspective	What the research perspective means for evaluation	Example of study design used
Efficacy: can this work in ideal circumstances?	Answers research questions about whether or not the intervention works in experimental settings. It aims to assess whether or not targeting the hypothesised mechanisms of action of an intervention produces changes in the expected outcome, often a mediating measure (e.g. disease activity), rather than a health or health-care outcome. The aim is to obtain a precise, unbiased estimate of the average effect of the intervention in a homogeneous context. In an efficacy study, the experimental treatment is compared with a placebo or with no intervention	*Case study 15**:*** efficacy RCT for carotid endarectomy^174^
Effectiveness: what works in the real world?	Answers research questions about whether or not the intervention works in pragmatic real-world settings. The principal focus is to identify an unbiased estimate of the average effect of the intervention, in a heterogeneous context that is representative of the scenarios in which the intervention will be applied in practice. In an effectiveness study, the comparison is with ‘treatment as usual’or sometimes with an optimised version of treatment as usual	*Case study 16**:*** individual RCT to evaluate an intervention for preventing repeat falls in people with Parkinson’s disease (PDSAFE)^175^ *Case study 14**:*** cluster RCT, with embedded process evaluation, to evaluate an approach to support the management of multimorbidity^176^‘^177^ *Case study 17**:*** stepped-wedge cluster RCT to evaluate the World Health Organization surgical checklist^178^ *Case study 18**:*** synthetic controls to evaluate pay or performance on hospital mortality^179^ *Case study 19**:*** whole systems trial for prevention of obesity^180^
Theory based: what works in which circumstances and how?	This perspective emphasises the importance of understanding how intervention mechanisms may generate one or more outcomes that may vary in different contexts and for different people. This approach places less value on the average effect estimate and more value on understanding the interplay of mechanism and context, how change is being brought about and how this may vary across different contexts and recipients	*Case study 20*: realist process evaluation to evaluate robotic-assisted surgery^181,182^ *Case study 21**:*** realist process evaluation using case study to evaluate an intervention to support adherence to antiretroviral treatment and retention in care^183^ *Case study 22**:*** mixed-methods evaluation of the New Deal for Communities^184^
Systems: how do the system and intervention adapt to one another?	Treats the intervention as a change or disruption to a complex system and/or to relationships within the system. For example, it focuses on the way that a system’s constituent parts inter-relate and how systems work over time and within the context of larger systems. It recognises the dynamic nature of systems and specific properties of that dynamic interdependence, such as emergence and feedback. Examples given here include modelling studies, which are not evaluations in the traditional sense, but simulations of what might happen under a given set of circumstances	*Case study 23**:*** social network analysis (and other systems methods) to evaluate a developmental asset approach to adolescent well-being^185^ *Case study 24**:*** process evaluation, taking a systems perspective, of a local alcohol availability intervention (Reducing the Strength)^186,187^ *Case study 25*: QCA to evaluate factors associated with narrowing health inequalities^188^

QCA, qualitative comparative analysis.

Links to case studies are included in Table 6 to highlight how different study designs may be used to answer different types of questions. A short description of a selection of these methods is given in *Types of evaluation design and methods*; however, before deciding on study design, the range of outcomes that an evaluation may include should be considered, which is described in the next section.

#### Choice of evaluation outcomes

A crucial aspect of the design of an evaluation is the choice of outcome measures or evidence of change. Evaluators need to assess which outcomes are the most important for stakeholders (e.g. patients, public and decision-makers) and how to deal with multiple outcomes in the analysis.

A sharp distinction between a single primary outcome and a number of secondary outcomes is not necessarily appropriate, particularly where the programme theory identifies impacts across a range of domains. A good theoretical understanding of the intervention, derived from careful development work, is key to choosing suitable outcome measures. Rather than always choosing one health outcome and judging the effectiveness based on that only, effects on intermediate outcomes, such as health behaviour, health care or a determinant of health or a range of outcomes, should be explored and theoretically justified. These outcomes should be identified prospectively during the process of developing the programme theory and be incorporated alongside an a priori analysis plan in a published study protocol and reported comprehensively in the final evaluation. This approach allows a more broad-based judgement on effectiveness to be made.

Inconsistencies in measured outcomes makes synthesis of evidence more difficult; therefore, the use of core outcome sets is recommended where appropriate.^192^ The Core Outcome Measures in Effectiveness Trials (COMET) initiative supports the development of core outcome sets, largely for clinical trials, although includes some resources that may be more widely applicable.^193^

Outcome measures may capture changes to a system rather than changes in individuals. Examples of system-level outcomes include changes in relationships within an organisation, the introduction of policies or new ways of working and changes in social norms or normalisation of practice. They also include how changing the dynamics of one part of a system alters behaviours in other parts, such as the displacement of smoking into the home after a public smoking ban. It is possible to use traditional evaluation designs to answer questions relevant to a systems perspective (see *Case study 19*). It is unlikely that all aspects of complexity can be investigated in an evaluation;^62^ however, a broad awareness of the system should be considered at the development phase and system boundaries determined, with consideration of the usefulness of evidence that will be generated.

Pre-identified sources of variation in outcomes are important, and prespecified subgroup analyses should be carried out and reported. In the case of interventions that are expected to affect inequalities in health, analyses stratified by socioeconomic position, gender, ethnicity or geography may be appropriate. Even where such analyses are underpowered they should be included in the protocol and reported, as they may be useful as input into subsequent meta-analyses or for developing hypotheses for testing in further research.

A priori sample size calculations are made to ensure that there is enough statistical power to detect a meaningful target difference in the outcome between the intervention and the comparison groups. This calculation is often based on a single primary outcome, although different candidate outcomes can be considered in turn. The final decision for sample size will depend on practicality and usefulness of evidence, and ideally will be sufficient to detect a meaningful change in all specified candidate outcomes. The Difference ELicitation in TriAls (DELTA2) guidance^194^ provides guidance on choosing target differences in outcomes and on associated sample size calculations.

Another outcome of evaluation is refined or developed theory. Improved theory helps us to understand the possibility of transferability across settings and to produce evidence and understanding that is more applicable to informing future decisions by policy-makers. Theory is used to inform evaluations, but the purpose of evaluation should also be the gradual incremental testing and refinement of theory. Interventions are likely to require refinement or adaptation to be successful in different contexts, whereas the theory produced from the study can be generalisable. It is important that results from experimental trials are not overinterpreted; unbiased estimates may not provide evidence suited to the application of the intervention in different contexts.^172^ Decision-makers operating in a context different from that of the research may be able to learn more from the theory that is developed from the intervention than from an effect estimate with little information on context or transferability.

#### Types of evaluation design and methods

There are many study designs to choose from and different designs are each optimally suited to different research questions and different circumstances.^195^ As we have emphasised, there is no one-to-one mapping of research perspectives to evaluation designs. A purely quantitative approach using an experimental design with no additional elements, such as a process evaluation, is rarely adequate. This is particularly true in complexity-informed research using a theory-based or systems perspective; in such cases, qualitative and mixed-methods designs are essential for evaluation to answer questions beyond effectiveness.

Studies that use both qualitative and quantitative methods often analyse the data for each method separately and then consider them together in discussion of the findings. There are techniques for integrating qualitative and quantitative data and findings;^196–198^ however, this is not common practice in the context of RCTs for health interventions, and effort is ongoing to provide future guidance (see Richards *et al*.^199^ for some examples).

A design may rarely be used in a particular field, but that does not mean it cannot be used. You should make your choice on the basis of the specific needs of your evaluation, in particular the research questions that you aim to answer. Awareness of the range of experimental and non-experimental approaches should lead to more appropriate methodological choices. In the following sections we elaborate on some of the evaluation designs from the case studies referred to in Table 6 and in *Case studies*. We also include social network analysis and modelling, which are not designs as such but provide an overview of further methods that can be used to evaluate complex interventions. We focus on experimental and theory-based designs that have come to the fore since the previous version of this document^1^ and the natural experiments guidance^200^ were published. The examples that we give of systems methodologies are those most commonly discussed in relation to systems methods for evaluation of interventions within health research, not necessarily an exhaustive list of systems methodologies.

##### Stepped-wedge designs

This is a variant of the cluster RCT design. It can be used to address practical or ethics objections to experimentally evaluating an intervention for which there is some initial evidence of effectiveness or that cannot be made available to the whole population at once. It allows a trial to be conducted without delaying roll-out of the intervention. Eventually, the whole population receives the intervention, but in a phased implementation. Allocation of the intervention can be undertaken in a randomised or non-randomised manner.^201^ This design is increasingly being used and provides an alternative to regular cluster RCTs, for which there are pragmatic constraints on randomisation or allocation of the intervention to groups.^202^ Given that the roll-out of the intervention in a stepped-wedge design has a temporal element, particular consideration should be given to any changes to the context that occur concurrently. Stepped-wedge designs should be used with caution given that they introduce many complexities and there are ethics and resource implications of delivering to all study participants what the study may find to be an ineffective or even harmful intervention.

##### Adaptive trial designs

This trial design involves advanced planning of modification to the evaluative design throughout the study period based on interim data.^203^ Adaptations to evaluations could include, for example, the decision to drop inferior trial arms, reassessment of sample size requirements, reassessment of eligibility criteria or changes to analytical methods. Adaptation of the evaluation is based on careful planning, simulation of potential scenarios and resulting decision rules. Interim data are evaluated in accordance with predefined decision criteria and any changes that are made require maintaining integrity and validity of the study.^204^ The aims of adaptive evaluations are to address uncertainties in the planning process and to identify promising interventions by eliminating those with little likelihood of benefit and reduce the probability of failure early on. One example of an adaptive trial design is Sequential Multiple Assignment Randomised Trial (SMART),^205^,^206^ in which participants are re-randomised to alternative intervention conditions depending on their responsiveness to the first intervention sequence. Typically, unresponsive participants are re-allocated to alternative interventions. SMART is also being used to refine interventions to optimise the delivery, duration or frequency.^205^

##### Natural experimental designs

Natural experimental studies exploit a process or event that occurs independently of the researcher and divides a population into exposed and unexposed groups, between which outcomes can be compared to identify the effect of the intervention. There is increasing recognition of the value of using these designs and considerable methodological development.^159,207,208^ Sometimes this process is effectively random, either by design^209^ or by some imperfection in the way that the intervention is implemented; usually a statistical model is required to create comparable or ‘conditionally exchangeable’ groups. Alongside well-established approaches, such as interrupted time series, difference-in-difference and regression discontinuity studies,^210–214^ there are some newer methods, such as synthetic controls, for which experience is accumulating rapidly.^215^ The synthetic control method involves creating a counterfactual from a pool of control units such that the pre-intervention trend in the outcome is similar in the exposed unit (usually a geographical area) and the synthetic control. Mixed-methods evaluations of natural experiments, as with other effectiveness studies, can provide further insight into intervention mechanisms and processes.^216^ Qualitative evaluations of natural experiments can also provide more in-depth information on the mechanisms by which outcomes are achieved.^217^ A natural experimental approach may be appropriate when a RCT is not practical or ethical; where a significant health impact is expected but there is uncertainty about the nature and size of the effect; or where the intervention has potential for being replicated and scaled up.^159^ Other advantages of natural experimental approaches are that they can be applied retrospectively to interventions that were implemented without any prospective evaluation using routinely collected data; produce evidence relatively quickly and cheaply; generate evidence with high external validity while minimising threats to internal validity; and identify effects on long-term and non-health outcomes.^218^ Further detail is provided by the MRC’s guidance on natural experiments^200^ and in a series of papers on quasi-experimental studies in the *Journal of Clinical Epidiomiology*.^219^

##### n-of-1 trials

An n-of-1 trial is a randomised controlled crossover trial with a single unit (e.g. person, hospital and school) that receives different intervention options and, therefore, acts as its own control.^220^ The unit of randomisation is not the individual but the intervention order within the cycle allocated to the individual. This allows the evaluation of how intervention effects vary within individuals, answering research questions about individual cases.^221^ A traditional trial would provide the average effect of an intervention for the group that received it, but would not necessarily provide information on differences in effect between individuals; n-of-1 trials are particularly useful for testing interventions and theory at the individual level, potentially providing evidence that allows for personalisation of interventions. In addition, an aggregated series of n-of-1 trials can be used to determine the effect of the intervention, including heterogeneity of effects, at the population level. However, challenges include the difficulty of calculating statistical power and sample size, and the fact that statistical approaches are as-yet underdeveloped/tested for this type of study design. For examples of *n*-of-1 trial designs being used in health behaviour research see McDonald *et al*.’s^221^ systematic review, and for medical intervention see Gabler *et al*.’s^222^ systematic review.

##### Network analysis

Network analysis is an approach that can be applied within the other study designs already mentioned, rather than being a design in itself. It is a tool to understand the structures of systems by studying changing relationships within a system, for example of individuals or organisations. It is useful either where the outcome is connections within a system structure, for example an evaluation of an intervention to tackle social isolation and improve social networks, or where these connections are part of the process, for example Rosas and Knight^185^ show change in actor networks between year 1 and year 2 of a community intervention designed to address adolescent health and well-being (see *Case study 23*).^185^ This method can also provide information on the agents that are central to the network or on who may be ‘influencers’ within the network,^223^ as well as on understanding how the intervention works.

##### Modelling

This approach is distinct from traditional evaluation methods in that it is used to predict intervention effects by simulating outcomes. It can, therefore, be useful in intervention development and justification, as well as being used in the evaluation phase as data are collected to assess the model predictions and/or to further improve the model. Microsimulation modelling predicts the effects of an intervention using known relationships between exposures and outcomes to model the effect of a change in exposure. Agent-based and system dynamics modelling methods use computational simulations that draw on objectively measured and experiential data from a range of perspectives. The starting point for these methods is often other soft systems methodologies. These models can be used to evaluate different hypothetical scenarios, for example different variants of an intervention, or to explore spill-over or longer-term effects on health or economic outcomes, which may occur outside the scope or time frame of an empirical evaluation. For example, in the Evaluation of the health impacts of the UK Treasury SDIL (see *Case study 7*) outcomes are measured at 2 years, with additional modelling to estimate effects for a wider range of health outcomes (e.g. morbidity and mortality, as well as health service costs) over 5–20 years.^224^

Models may, thus, be used alone or to complement other designs and can be used throughout research phases, including development and implementation as well as evaluation. These methods are just emerging in health-related evaluation research, with few examples as yet. ABM involves virtual representation of real-world situations that models individuals and their interactions with their environment, which allows for system properties, such as feedback and emergence.^225^ See Tracy *et al*.^226^ for an overview of ABM in public health, with examples of application and discussion of future direction (see also Silverman *et al*.^122^). System dynamics modelling is an approach that aims to predict the behaviour of complex systems over time, where the starting point is the structures that trigger the behaviours of the system itself, rather than the entities within it (as in agent-based models). As yet, less work has been carried out using system dynamic modelling in evaluative health research.

Other modelling methods also use synthetic populations and simulate individual trajectories under different scenarios. For example, the effectiveness of the NHS health check programme in Liverpool was modelled from 2017 to 2040 under five scenarios, including continuation of the implementation of the programme as it is and targeting implementation to the most deprived areas, with comparison with no NHS health check.^227^ The study showed that under current implementation the intervention was unlikely to be cost-effective or equitable, but that implementation under alternative scenarios would be more positive. Additional examples that use this (IMPACT_NCD_ model^228^) and other models have explored salt policies in England; the potential of trans fats policies to reduce socioeconomic inequalities in coronary heart disease mortality in England;^229^ and the impact of pandemic influenza on the UK.^230^ The Sheffield Alcohol Policy Model was used to predict the effect of minimum unit pricing for alcohol in Scotland, including differential impacts on heavy and light drinkers and by socioeconomic group; this model was key to the development of the policy and in countering legal challenges to its implementation and will continue to be used in the evaluation of the policy as data accumulate.^231^

##### Qualitative comparative analysis

Qualitative comparative analysis (QCA) is a mixed method for establishing causation based on systematic qualitative and quantitative comparison across a number of cases, for example intervention sites and exploring how outcomes were achieved.^232–234^ Qualitative attention to each case is followed by quantitative exploration of each case by a number of attributes determined to be important. These attributes are defined through careful review of existing and new data, for which stakeholder engagement and theoretical arguments are crucial (see *Case study* 25).^188^ The QCA approach does not specify a single causal model but follows the principle of equifinality, which means that the same outcomes can be achieved by different means, that is there can be more than one causal set.

#### Understanding processes

Process evaluation is a critically important component of the evaluation of complex interventions and is an essential adjunct to the study designs presented so far (see the case study examples set out in Table 6). Process evaluations can use qualitative and/or quantitative process data to answer questions around fidelity and quality of implementation (e.g. what is implemented and how?), mechanisms of change (e.g. how does the delivered intervention produce change?) and context (e.g. how does context affect implementation and outcomes?).^165^

Within an efficacy study, a process evaluation would have relatively narrow questions. The key idea of efficacy studies is that there is a test of causal mechanism, often through mediation analysis. Such analyses are usually quantitative, but could use qualitative methods to assess how research participants understood the intervention and made use of intervention components. Because the focus of efficacy studies is to maximise internal validity, process analyses could usefully examine fidelity to assess whether or not the intervention was delivered with high fidelity to the theorised mechanism of action. Consideration of context would not be the focus of a process analysis from an efficacy perspective.

Process evaluations in an effectiveness perspective would be used to inform findings, for example to provide insight into why an intervention fails unexpectedly or has unanticipated consequences, or why a successful intervention works and how it can be optimised. This includes exploring the fidelity and quality of the intervention, clarifying causal mechanisms and identifying contextual factors associated with variation in outcomes (see *Case study 14*). Details on the type of measures to consider are provided in MRC guidance on process evaluation of complex interventions.^5^

Process evaluations are key components of theory-based and systems evaluations, to the extent that they seek to answer research questions about how the intervention produces change in a generalisable way, taking the study further than assessing solely whether or not it works in a specific setting. An emerging approach is to conduct a realist process evaluation, specifically drawing on realist evaluation.^48^ If undertaken alongside an effectiveness evaluation, a realist process evaluation can help to understand the effects of intervention components rather than only the intervention as a whole; examine mechanisms of change; explore the effect of intervention context; collect and analyse qualitative and quantitative data; and aim to build and validate intervention programme theories.^235^ There are also increasing examples of taking a systems approach to process evaluations, with a framework developed by McGill *et al*.^236^ offering some guidance.

Process evaluations should be conducted to the same high methodological standards and reported as thoroughly as evaluation of outcomes. The MRC guidance on process evaluation for complex interventions^5^ provides further information on how to conduct a process evaluation, including consideration of relevant theoretical approaches.

#### Economic considerations

As noted in *Economic considerations*, there are a range of economic evaluation frameworks (Box 7).

Each framework has different strengths and limitations. CEA measures outcomes in natural units that relate to, but do not measure directly, the health outcomes of interest; for example, reduced blood pressure is associated with reduced incidence of stroke, but it does not directly measure the incidence of stroke or capture the associated range of health improvements. This limits the usefulness of an incremental cost-effectiveness ratio when evaluating complex interventions with multiple impacts. CUA uses measures, such as quality-adjusted life-years (QALYs), to compare interventions with a range of physical and mental health outcomes in terms of their impact on length and quality of life. However, complex interventions often have outcomes that are difficult to capture fully using QALYs. Ascribing monetary values to all of the dimensions of benefit in a CBA is methodologically challenging. CCA is more straightforward and can be used to present evidence on a wide range of costs and outcomes; however, without valuation of these outcomes (e.g. preference weighting in QALYs or monetary valuation in CBA) decision-making requires judgement. Rare but costly outcomes may acquire too much influence if their frequency is not taken into account. Furthermore, if some outcomes improve more under some interventions and other outcomes fare better under others, the policy implications of a CCA may be ambiguous. The importance or ‘weight’ of differing consequences is likely to vary across stakeholders. A separate exercise, such as a discrete choice experiment (see *Case study 26*), could add additional evidence to the value of different consequences to different stakeholders.

Given that complex interventions typically have effects across a wide and diverse range of outcomes, recent guidance emphasises the use of a broad framework, such as CCA or CBA.^134^ CCA has the intuitive appeal of providing data across a range of ‘consequences’ (outcomes) with which decisionmakers are likely to be concerned when making a policy decision. This allows the analyst to adopt a broad public sector or societal perspective that is appropriate to many complex interventions. It is important to note, however, that the different frameworks are not mutually exclusive: CBA and CEA can be carried out and embedded in a wider CCA that provides data on a more comprehensive inventory of outcomes. The CCA can be useful for identifying the range of impacts likely to be of interest to researchers and policy-makers prior to deciding which outcomes will be measured and how. For this reason, it can be a helpful first step towards a CBA.

In addition, there are a number of emerging methodologies that can lend themselves to economic evaluations of complex interventions. They include Social Return on Investment^237^ and multi-criteria decision analysis (MCDA).^238^ These are not usually considered to be standard tools for economic evaluation owing to theoretical weaknesses and their handling of outcomes, but they are becoming more commonly used in the area of complex interventions. MCDA is particularly helpful following a CCA because it involves a structured, explicit approach to decisions involving multiple criteria.^238^

Decision analytic modelling^120,239^ is a useful way of combining data from an economic evaluation with data from other sources to extrapolate costs and outcomes beyond the study time frame. The use of advanced modelling techniques, such as microsimulation modelling, network analysis, discrete event simulation and ABM,^153,240,241^ is particularly useful for complex and public health interventions, although simpler model types can also be appropriate.^242,243^

#### Reporting evaluations

Evaluations should always be reported in accordance with established guidelines because this will help to ensure that the key information is available for replication studies, evidence syntheses and guideline development. The Enhancing the QUAlity and Transparency Of health Research (EQUATOR) Network maintains a comprehensive database of reporting guidelines [www.equator-network.org/ (accessed 2 March 2021)].

Relevant guidelines for reporting are as follows:

- The CONSORT statement is a well-established standard for reporting randomised trials. Relevant extensions are published for –
  ∘ social and psychological interventions (CONSORT-SPI 2018)^244^
  ∘ randomised pilot and feasibility trials^163^
  ∘ cluster RCTs^245^
  ∘ pragmatic trials^246^
  ∘ *n*-of-1 trials^247^
  ∘ cluster stepped-wedge trials^248^
  ∘ non-pharmacological interventions^249^
  ∘ patient-reported outcomes (CONSORT PRO)^250^
  ∘ adaptive designs^251^
  ∘ and the SPIRIT-PRO extension for patient-reported outcomes.^252^
- Reporting of statistical analysis plans.^253^
- Reporting process.^254^
- RAMESES II for realist evaluation.^255^
- SPIRIT 2013 statement provides evidence-based recommendations for the minimum content of a clinical trial protocol.^256^
- Standards for reporting qualitative research.^257^
- STrengthening the Reporting of OBservational studies in Epidemiology (STROBE).^258^
- Consolidated Health Economic Evaluation Reporting Standards (CHEERS).^170^

Table 7 provides a summary of the consideration of each of the core elements at the evaluation phase.

**Table 7:** Core elements for the evaluation phase

Core elements	Issues to consider	Risk of not considering them
Context	Consider the wider contextual factors that may influence the evaluation in relation to influences on either the ‘intervention’or the ‘study processes’	Questions related to external validity will not be answered
Programme theory	Has the programme theory changed from the development and/or feasibility phase? What aspects of the wider system have influenced the desired outcomes?	Lack of potential learning and understanding owing to either lack of programme theory or underdeveloped programme theory
Stakeholders	Gather appropriate input from a range of relevant stakeholders to ensure that evaluation methods and outcomes meet their requirements	Not collecting the correct data to inform changes in policy and practice could negatively impact the utility of the evaluation and future implementation of the intervention
Uncertainty	The evaluation should respond to key uncertainties identified by engaging with stakeholders, existing evidence and relevant theory	Answering research questions that do not address important areas of uncertainty, so that the utility of the evaluation will be limited
Intervention refinement	Decide beforehand what type of refinement is appropriate to allow for a robust evaluation	Not allowing for appropriate changes to the intervention or study processes may lead to outcomes that are not useful or meaningful. Validity of the evaluation may be compromised by failing to consider what kind of intervention refinement is acceptable
Economic considerations	Importance of (broad) perspective Sensitivity analysis and subgroup analyses will be crucial Identify preferred economic evaluation framework (other frameworks can be embedded) Consider both a short-term and a long-term time horizon for economic analyses	Too narrow a perspective will not reveal the full economic impact Cost-effectiveness of alternative scenarios will not be identified Incorrect frameworks may not identify all relevant costs and impacts Ignoring long-term costs and outcomes may result in an incorrect cost-effectiveness conclusion

**Key Points 10 Evaluation**

- Where appropriate, involve stakeholders in defining research questions and the choice of evaluation design and outcomes to ensure that meaningful and useful data are produced for relevant stakeholders.
- Determine the key uncertainties that your evaluation will resolve and frame appropriate research questions.
- Consider a range of ‘useful’ evaluation perspectives and outcomes rather than necessarily focusing on effectiveness.
- Choose from a range of experimental or non-experimental designs that are focused on answering the research questions.
- Include methods of evaluation to explore the change process (i.e. mechanisms, process, context and theory).
- Conduct economic evaluation and consider the range of impacts that an intervention may have.
- Clearly report the evaluation, including updated programme theory, following recognised reporting standards if available.

**Signposts to Further Reading 10 Evaluation**

- For detail on different experimental designs, see the 2006 MRC guidance.^1^
- PRagmatic Explanatory Continuum Indicator Summary (PRECIS and PRECIS-2) as a tool to help determine whether or not a trial should be/is on the pragmatic–explanatory continuum.^39,259^
- For guidance on sample size decisions see the DELTA2 guidance^194^ and a series of papers in *Trials*(2018, issue 19).^260^
- The COMET database provides a searchable set of developed core outcome sets for various health areas and target populations.^193^
- MRC guidance on natural experiments.^9^
- Synthetic controls.^215^
- Stepped-wedge cluster randomised trials.^202,248^
- Commentary and hypothetical example on how theories of change and realistic evaluation differ.^44^
- Qualitative comparative analysis for evaluation.^233^
- Discussion and examples of treating qualitative and quantitative data from a RCT interdependently.^199^

**Economic evaluation**

- Further reading and advances in the field overall.^132,141,242,243,261,262^
- NICE guidance.^134^ The NICE guidance, updated in 2017,^134^ allows wider perspectives (e.g. NHS and Personal Social Services, local government and societal where appropriate), inclusion of alternative non-health outcomes (such as the ICECAP^263^ measurement of capabilities^264^ and ‘broader’ approaches such as CCA and CBA, as appropriate).^92^
- Alongside observational studies and natural experiments,^265,266^ economic modelling^243^ and conceptual modelling frameworks,^140^ applied health economic methods, public health practice and research,^141^ and reporting guidelines.^170^

### Implementation

‘Implementation’ is a term used inconsistently within and across disciplines. Here, we use the World Health Organization (WHO) definition:

Implementation: Deliberate efforts to increase the impact of successfully tested health innovations so as to benefit more people and to foster policy and programme development on a lasting basis.

*Reproduced from WHO*^267^

Successful implementation of interventions, even after they have been shown to be effective, remains relatively rare.^268^ There are numerous reasons for this: some relate to how intervention research is conducted and others focus more on how research findings translate into policy and practice. In this section, we focus on how the conduct and reporting of intervention research can be undertaken to maximise its utility and impact. First, we briefly discuss implementation studies that focus specifically on interventions and strategies to maximise the translation of research into practice or policy.

#### Implementation science

Within health care, implementation science is an important and developing field of research that undertakes studies that develop and evaluate strategies for implementing effective interventions that address local realities, can be implemented at scale and are potentially sustainable.^269^ Implementation science has been defined as ‘the scientific study of methods to promote the systematic uptake of research findings and other evidence-based practices into routine practice, and, hence, to improve the quality and effectiveness of health services’;^270^ however, a wider definition would not be restricted to health care or to the limited range of designs and methods typically used in that context. With this wider definition, implementation science, therefore, includes interventions and strategies to increase the uptake and impact of research in policy, including knowledge mobilisation across a range of potentially relevant organisations, sectors and professions, as exemplified by the range of case studies of implementation research in a global health context.^271^

A large part of implementation science research, therefore, involves the development and evaluation of complex interventions to maximise effective implementation in practice and/or the policy of interventions that have already demonstrated efficacy or effectiveness. The whole of this framework, therefore, applies to those implementation studies just as it does to other forms of intervention. The main specific characteristic of such implementation studies is that the main outcome measures used will not be health outcomes but measures of implementation effectiveness, such as practitioner or patient behaviour, uptake of services, reach and policy impact. For example, a cluster randomised trial of strategies to improve nurses’ hand-washing behaviour used hand-hygiene compliance and adherence as outcome measures rather than infection rates, with a nested process evaluation identifying which components of the implementation strategy and context particularly supported compliance.^272^ The field of implementation science has many theories and frameworks that can importantly inform the development of implementation interventions and associated programmes (see *Signposts to furtherreading 11: implementation*). There are also specific standards for reporting implementation studies and other specific considerations for implementation-focused intervention research that complement this framework.^273^

#### Implementation in all phases

A key motivation for this update was to maximise the usefulness of complex intervention evidence for decision-makers. For the evidence to be useful, it is important that interventions are developed with implementation considerations in the foreground at all phases (see *Case study 27*). A key part of this is involving stakeholders and being mindful of the realities of the intervention context at all phases/from the beginning and throughout. Early consideration of implementation increases the potential to develop an intervention that can be widely adopted and maintained in real-world settings.^245^ For example, this may encourage the development of ‘very brief interventions’ that fit with routine practice and are effective and cost-effective when implemented at scale.^274^ Such interventions may have more impact than very ambitious researcher-led interventions that demonstrate effectiveness and costeffectiveness in a research setting, but are not ‘implementable’ for a variety of reasons, such as lack of fit to health-care practitioners’usual practice.^130^ The consequence of neglect is that issues, such as acceptability to the public or to those who deliver the intervention, may undermine implementation later on.^275^ Other benefits of including stakeholders in all phases are to gain better understanding of the time and resource constraints of real-world evidence users and to engage with, understand and influence organisational, professional and social systems that may be resistant to change.^276–279^

As well as anticipating implementation factors in intervention development and design, research questions in the feasibility and evaluation phases can help to understand and inform implementation. The intervention programme theory and process and outcome data collected in these phases can help inform dissemination of the findings and preparation of an implementation manual or plan to maximise the effective roll-out of the intervention in real-world settings. In addition to the publication of findings, sufficient details of the intervention and its underlying theory should be reported.^273^ This improves the synthesis of evidence that may be more useful to decision-makers (e.g. theoretical outputs compared with effect sizes) and improves their understanding of the potential transferability of the intervention to their specific context (see *The role of context in implementation*).

Details of the intervention and key information related to effective delivery identified in feasibility and evaluation phases need to be communicated clearly in dissemination via methods that are accessible and convincing to implementers.^280^ These may include resource requirements for prerequisites and delivery (e.g. equipment, staff training, skills and time); barriers to and facilitators of effective implementation; and potential flexibility in implementation while maintaining programme fidelity. This includes making recommendations as specific as possible.^281^

#### Implementation and evaluation phases combined

In a natural experimental study, evaluation takes place within the implementation of the intervention in a real-world context. It may be possible to ascertain both the principal health outcomes during implementation to demonstrate effectiveness and/or the more proximal or process measures available through readily available data sources collected as part of the implementation. This would allow the assessment of which implementation or contextual factors support, amplify or undermine the uptake, (cost-) effectiveness or reach of the intervention.

In the case of highly pragmatic effectiveness trials or specific hybrid effectiveness–implementation designs, effectiveness and implementation outcomes can be combined in one study, with the aim of reducing time for translation of research on effectiveness into routine practice. Different hybrid designs have been proposed based on their focus on effectiveness versus implementation: conducting an effectiveness study while exploring and gathering data on implementation; dual focus on effectiveness and implementation; and conducting a study with a primary focus on implementation, while gathering data on effectiveness.^282,283^ Green *et al*.^284^ describe three studies to show how these approaches differ in practice.

#### The role of context in implementation

Understanding interdependence with the context of a given intervention is critical to the success or failure of implementation. Developing an understanding of context dependence throughout the development, feasibility and evaluation phases can, therefore, importantly inform what prerequisites need to be in place for the effects identified in the evaluation phase to be successfully reproduced in implementation.

Understanding context is also central to the transferability of an intervention into different contexts: an important aspect of long-term implementation. Developing and refining programme theory and considering wide-ranging contextual factors can help to identify issues related to transferability, for example that the intervention is unlikely to be effective when transferred to a given context.^285^ Stakeholder involvement is then a helpful process to identify potential solutions to overcome identified issues.

The ‘Context and Implementation in Complex Interventions’ framework that integrates context, implementation and setting can be used to guide discussions between researchers and stakeholders on the transferability of interventions.^70^

#### Economic considerations of implementation

Economic issues at the implementation phase have not traditionally formed part of the guidance on economic evaluation, but they can and should be considered (1) at the early stages of intervention and study development and (2) in the reporting of economic results.

How the results of economic analyses are reported and presented to decision-makers can affect whether or not and how they act on the results. A key issue is avoiding and anticipating misconceptions in reporting the results of economic analyses. Interventions described as ‘cost saving’ may well be potentially cost saving, but savings may be difficult to realise in financial terms during implementation if there is an element of fixed costs or if the reduced demand for a service withdrawn on economic grounds is simply replaced with demand for services to meet previously unmet needs. The ‘cost saving’ may refer not to a ‘financial’ saving but to a release of resources for other potential uses, such as a freeing up of beds or the shifting of resource use from the NHS to another sector, such as social services (e.g. palliative care).^286^

Another important issue that is relevant for implementation is how to deal explicitly with multisectoral issues. The payers of costs and the receivers of benefits of a multisector intervention may not be the same organisation, which can reduce the incentive to implement an intervention even if it is shown to be beneficial relative to its cost. Early identification and engagement with appropriate stakeholders and decision-makers to identify such cross-sectoral impacts will help frame appropriate research questions and pre-empt any implementation challenges that might arise. Recent work in this area has suggested methods, such as co-financing between and across sectors,^261^ to deal with these issues.

Studies to assess the cost of implementation can also be undertaken post evaluation to help aid the implementation process. This could be particularly important where an intervention is proven to be effective and cost-effective, yet implementation requires additional investment.

#### Other considerations specific to the implementation phase

At the implementation phase, observational or experimental studies are often useful even though there may not be any specific implementation strategies being assessed, as would be the case in implementation science studies (see *Implementation science*) or hybrid implementation/effectiveness designs (see *Implementation and evaluation phases combined*). Questions that can be assessed at the implementation stage include assessment of intervention receipt, ‘dose’ received, programme completion and the characteristics of those receiving the intervention and any inequalities in reach or completion. Similarly, barriers to and facilitators of effective implementation, including contextual, system or resource constraints, can be investigated. A key issue in understanding implementation is the extent to which the intervention has been delivered with fidelity,^287^ so that problems with implementation can be distinguished from a lack of effectiveness. Where an intervention is delivered with low fidelity, it is difficult to determine whether or not a lack of impact in implementation reflects an intervention that is genuinely ineffective, ineffective in that context or potentially effective but not observable in this instance owing to low-fidelity implementation. On the other hand, some flexibility in intervention implementation may support intervention effectiveness across contexts, provided that the key functions of the programme are maintained.^40^ Where adaptations have been made to an intervention in a particular context, it is important to understand exactly what has been adapted.^145^ Further detail on implementation related to fidelity can be found in the MRC’s guidance on process evaluation of complex interventions.^5^

Few evaluation studies are powered to detect rare events, while unanticipated or adverse outcomes and potentially important longer-term system effects of interventions, such as spill over or diffusion of effects through networks, may also not be feasible to measure in an evaluation study within a specific sample and time frame. These can potentially be measured within the implementation phase, as they may have differential reach or effectiveness across population subgroups.^288^ Effects are likely to be smaller and more variable once the intervention becomes implemented more widely, and unintended consequences may begin to emerge.^76,136^ Long-term follow-up may be required to determine whether or not short-term changes persist, to determine whether or not benefits demonstrated from the original study do in fact occur and to capture any likely cost impacts (e.g. cost savings). Although long-term follow-up of complex interventions is uncommon, such studies can be highly informative. It is worth thinking about how to measure rare or long-term impacts, for example through routine data sources and record linkage or by recontacting study participants. Plans for the collection of appropriate outcome data and obtaining appropriate consents should be built into the study design at the outset. Implementation phase studies may involve randomised or non-randomised control groups or can be undertaken solely among those who receive the intervention.

Table 8 provides a summary of the considerations of the core elements at the implementation phase.

**Table 8:** Core elements of the implementation phase

Core elements	Issues to consider	Risk of not considering them
Context	Consider the contextual factors that could influence the intervention in this and other settings (e.g. adoption, reach and maintenance of the intervention) What are the key factors that may determine whether or not evaluation findings are replicated in implementation?	Failure to understand how the intervention interacts and/or interrupts the system in which it is located Interventions implemented in contexts in which they are unlikely to work
Programme theory	What are the key functions and components of the intervention that need to be preserved in implementation to maximise the likelihood of achieving effects found in evaluation? Continue to update programme theory throughout any implementation phases What potential harms, spill-over effects or longer-term effects can be measured in implementation?	Implementation failure Failure to learn in the implementation phase about where fidelity, flexibility and refinement can be balanced to maintain intervention effects Failure to identify harms, side effects or potential wider, long-term effects that were not assessed in the evaluation
Stakeholders	Careful communication of research findings to stakeholders to support decision-making as to where and how interventions may be transferred and key aspects of programme implementation relevant to their context Identifying potential ‘champions’of the intervention to support its implementation	Intervention implemented in contexts in which the evaluation evidence is unlikely to generalise Implementation failure
Uncertainty	What are the areas of uncertainty in our implementation plan and theory? Are there key barriers to and facilitators of implementation in real-world contexts that have not been identified or theorised in other phases of research? What are the critical intervention components and functions that need to be maintained in implementation and where can there be flexibility in intervention form while preserving key functions? Are their effects not anticipated or measured in the previous stages that can occur and be assessed in implementation?	Evidence of effectiveness may not translate as the intervention is implemented outside research conditions Failure to continue to learn about how the intervention can best be implemented to maximise effects Failure to optimise implementation across contexts Failure to capture important unanticipated, rare or long-term system effects that were not included in the evaluation design
Intervention refinement	Refine the intervention, while retaining its core elements and programme theory, in response to implementation and contextual factors	Intervention failure owing to unnecessarily rigid adherence to intervention as evaluated
Economic considerations	Highlight and work towards multisectoral evaluation from the outset Establish who payers are and who receives benefits. Co-payment options may be relevant Cost of implementation analyses: overcome logistical barriers Consider requirements for additional sensitivity analysis to explore applicability of economic evaluation evidence to the differing context of large-scale implementation	Misconceptions of reported economic results Decision-makers misunderstanding economic results and what that means for their practice/budgets Cost-effective interventions not implemented in practice Cost-effectiveness of large-scale implementation underestimated or overestimated

**Key Points 11 Implementation**

- Determine key uncertainties about intervention implementation that need to be resolved to ensure impact, and develop appropriate research questions related to implementation.
- Consider implementation throughout all research phases.
- Involve stakeholders from the early development phase and throughout.
- Incorporate theories, models or frameworks of implementation into intervention design.
- Learn from where others have identified a ‘failure of implementation’.
- Incorporate economic considerations alongside implementation issues from outset. These should be considered in the programme theory developed at the development phase.
- Publish and actively disseminate full details of the intervention and the updated programme theory, including a clear indication of the critical functions of the intervention and key context dependencies.

**Signposts to Further Reading 11 Implementation**

- Implementation fidelity: the MRC guidance on process evaluation of complex interventions.^5^
- The Context and Implementation of Complex Interventions (CICI) Framework.^70^
- *Development of a framework and coding system for modifications and adaptations of evidencebased interventions*.^145^
- *Unintended outcomes evaluation approach: a plausible way to evaluate unintended outcomes of social development programmes*.^136^
- *Standards for reporting implementation studies (StaRI) statement*.^273^
- The Implementation Research (‘ImpRes’) tool.^289^
- An introduction to implementation science.^290^
- *Making sense of implementation theories, models and frameworks*.^291^
- *Normalisation process theory: a framework for developing, evaluating and implementing complex interventions*.^130^
- *Implementation research: new imperatives and opportunities in global health*.^271^
- *Diffusion of innovations in service organizations: systematic review and recommendations*.^292^
- *The quality implementation framework: a synthesis of critical steps in the implementation process*.^293^

## Case studies

This section includes 27 case studies that have been chosen to highlight key points throughout (Table 9).

**Table 9:** Case studies referred to in the report

Case study	Description
***Core elements***	
*Case study 1*	Travel through the main phases of complex intervention research
*Case study 2*	Considering context
*Case study 3*	Developing, refining and testing programme theory
*Case study 4*	Complex logic model
*Case study 5*	Dark logic model
*Case study 6*	Realist matrix
*Case study 7*	System map to develop programme theory
*Case study 8*	Engaging stakeholders (service users)
*Case study 9*	Refining the intervention
*Case study 10*	Economic considerations
***Phases of the research process***	
*Case study 11*	Intervention development and example systems map
*Case study 12*	Feasibility trial
*Case study 13*	Feasibility: evaluability assessment
*Case study 14*	Evaluation: effectiveness perspective - cluster RCT with theory-informed process evaluation
*Case study 15*	Evaluation: efficacy perspective
*Case study 16*	Evaluation: effectiveness perspective with individual RCT
*Case study 17*	Evaluation: effectiveness perspective with stepped-wedged cluster randomised design
*Case study 18*	Evaluation: effectiveness perspective with synthetic control design
*Case study 19*	Development and evaluation: effectiveness perspective drawing on a systems perspective for intervention development and evaluation
*Case study 20*	Evaluation: theory-based perspective alongside effectiveness
*Case study 21*	Evaluation: theory-based perspective with realist approach
*Case study 22*	Evaluation: theory-based perspective
*Case study 23*	Evaluation: systems perspective
*Case study 24*	Evaluation: systems perspective with process evaluation
*Case study 25*	Evaluation: systems perspective with qualitative comparative analysis
*Case study 26*	Intervention development and discrete choice experiments
*Case study 27*	Implementation in all phases

### Case study 1: travel through the main phases of complex intervention research

Study title: Football Fans In Training (FFIT).^294–298^

Summary: many men lack basic information about how to maintain a healthy weight by eating a balanced diet and staying physically active. They often feel uncomfortable about joining NHS or commercial weight management programmes, which they see as being mainly for women. FFIT aimed to help men lose at least 5–10% of their weight and keep it off over the long term. The programme was developed to appeal to Scottish football fans and to help them improve their eating and activity habits.

A RCT that was undertaken in professional football clubs established the effectiveness and cost-effectiveness of the FFIT programme; the mean difference between the intervention and the control groups at 12-month follow-up was 4.94 kg in favour of the intervention group. In addition to weight loss during the 12-week programme, almost 40% of participants maintained ≥ 5% weight loss at 12-month follow-up.This level of maintained weight loss is associated with various health benefits, including lower risk of stroke, heart disease, cancer, diabetes mellitus and other health issues. A CEA demonstrated that FFIT was inexpensive to deliver, making it appeal to decision-makers for local and national health provision.

The journey of the research project through the phases of complex intervention research is illustrated in Figure 5.

Key points:

- The study team considered all six core elements during each of the four phases of research.
- Implementation was considered from the outset and the study team engaged with key stakeholders in the development phase to explore how the intervention could be implemented in practice, if proven to be effective.

### Case study 2: considering context

Study title: *Why do interventions work in some places and not others: a breastfeeding support group trial*.^299^

Summary: the Breastfeeding In Groups intervention involved the provision of breastfeeding groups for pregnant and breastfeeding individuals and aimed to increase breastfeeding and maternal satisfaction. Findings from the effectiveness study (pragmatic cluster RCT) showed that breastfeeding rates increased in three intervention localities, declined in three and remained unchanged in one.^300^ This could not be explained by the quantity of the intervention delivered (number of group meetings or attendances). Qualitative and quantitative data were collected to develop seven case studies to explore differences in context in the different areas. The study found that the history and context of the locality, including structural and organisational factors, professional relationships, leadership, multidisciplinary partnership working and readiness to change, all influenced successful policy implementation.

Key points:

- This study was informed by a realist approach and offered insights into the influence of context on the impact of the intervention.
- The study chose methods in a pragmatic way and used ethnographic in-depth interviews, focus groups, observations and survey data to build case studies.
- Data collection and analysis were conducted before the findings of the outcome evaluation were known to minimise bias in interpretation. Retroductive logic was then applied to explain the outcomes from the effectiveness trial.
- Systems thinking was used to inform the analysis; for example, the localities were interpreted as systems composed of numerous interacting elements.
- Overall, the explanatory model that was constructed provided insights into the necessary attributes for successful policy initiation and sustainability. As well as providing a plausible explanation for the trial results, the model may be useful for other trials of complex interventions.

**Figure 5:** Case study demonstrating how the FFIT programme moved through the research process. SPFL, Scottish Premier Football League.

### Case study 3: developing, refining and testing programme theory

Study title: *Improving care at scale: process evaluation of a multi-component quality improvement intervention to reduce mortality after emergency abdominal surgery (EPOCH trial)*.^301^

Summary: this stepped-wedge cluster randomised trial tested a large-scale quality improvement programme that aimed to improve 90-day survival rates for patients undergoing emergency abdominal surgery.^302^ The programme theory was developed prior to evaluating the quality improvement programme. Current evidence and learning from other quality improvement programmes were used to justify inclusion of elements of the programme theory. Contextual factors were considered so that delivery of the programme theory could be tailored to local contexts; those leading the implementation in each hospital were supported to focus on the aspects of the intervention that would work in their local context, for example one consideration of context was the available human and financial resources. Desired outcomes were identified, along with the strategies, individual activities and resources intended to achieve those outcomes. Importantly, data collection was then aligned with elements of the programme theory to ensure that the mixed-methods process evaluation gathered relevant data to allow interpretation of the programme theory. Findings from the process evaluation, therefore, informed reflections on the programme theory. The overall findings suggested that some, but not all, aspects of the EPOCH programme theory were implementable in the context of hospital resources. The programme theory potentially underestimated the influence of social influences (e.g. the time-consuming and challenging nature of building and maintaining social relationships) and system influences (e.g. a major system reorganisation in the NHS) on the quality improvement programme. This helped to identify barriers to improvement, and feeds into the development of future quality improvement programmes.

Key points:

- The programme theory was well articulated throughout the paper.
- Contextual factors were considered allowing tailored programmes to be delivered in different hospitals.
- Data collection methods were aligned with elements of the programme theory to support interpretation of the findings.
- Overall, the development, implementation and post-study reflection of the programme theory resulted in suggested improvements for future implementation of the quality improvement programme.

### Case study 4: example of a complex logic model

Study title: *How to and how not to develop a theory of change to evaluate a complex intervention: reflections on an experience in the Democratic Republic of Congo*.^303^

Summary: the Democratic Republic of Congo faces many health system challenges, one of which is the flawed wage system for health workers. This has a number of impacts, including reduced motivation and inadequate delivery of health care. The authors outline the process of developing a theory of change to inform the development and evaluation of an intervention that sought to improve government payments to health workers. They developed a logic model in collaboration with key stakeholders that was reviewed before the intervention was implemented and again afterwards. The logic model (Figure 6) attempts to demonstrate the complexity of the intervention. Key findings from this work include the importance of ensuring that all relevant stakeholders are included, the challenge of managing power dynamics among stakeholders and the importance of exploring the impact of the intervention on the wider system.

**Figure 6:** Logic model of a complex intervention to improve payment of salaries to health workers. Reproduced with permission from Maini *et al*.^303^ This is an Open Access article distributed in accordance with the Creative Commons Attribution Non-Commercial (CC BY-NC 4.0) license, which permits others to distribute, remix, adapt, build upon this work non-commercially, and license their derivative works on different terms, provided the original work is properly cited and the use is non-commercial. See: http://creativecommons.org/licenses/by-nc/4.0/. The figure includes minor additions and formatting changes to the original figure. ASSP, Accès Aux Soins de Santé Primaires; CODESA, community health committee; DFID, Department for International Development; FP, family planning; HR, human resources; HRH, human resources for health; iHRIS, IntraHealth International (Chapel Hill, NC, USA); IMA, IMA World Health (Washington, DC, USA); MoH, Ministry of Health; SNIs, single-number indicators; WISN, Workload Indicator of Staffing Need. Sources included as per original.^304–306^

Key points:

- The logic model shows the resources and activities as well as the outcomes and impacts that you might expect to see in a more traditional logic model; however, it also includes assumptions, interventions, indicators and rationale.
- Importantly, the hypothesised mechanisms of change, including the pathways of each element, are considered.
- Contextual factors and their influence on the intervention processes are also included.

### Case study 5: example of a dark logic model

Study title: *Communities in charge of alcohol (CICA): a protocol for a stepped-wedge randomised controlled trial of an alcohol health champions programme*.^307^

Summary: this study evaluated an asset-based community development approach to reducing alcohol harm. Volunteers were trained to become ‘Alcohol Health Champions’, to provide advice to individuals and to mobilise community-level activity focused on alcohol availability. As part of the development work for the evaluation, the authors considered the possible unintended harms of the intervention (dark logic). They developed a ‘dark’ logic model that demonstrates how potential negative consequences could result from the intervention (Figure 7).

Key points:

- The development of a dark logic model in advance of the evaluation helps evaluators to include appropriate methods to measure possible negative consequences.
- The dark logic model could inform changes to the intervention and reduce the risk of future harm.

### Case study 6: example of a realist matrix-presenting programme theory

Study title: *Dealing with context in logic model development: reflections from a realist evaluation of a community health worker programme in Nigeria*.^308^

Summary: in this paper, the authors describe the development of a logic model within the context of an ‘ongoing realist evaluation which assesses the extent to which and under what circumstances a community health workers programme promotes access to maternity services in Nigeria’.^308^ The development of a logic model is seen as key to identifying relevant hypotheses regarding context, mechanisms and outcomes, and how interventions lead to change. As part of the process of developing the logic model and to help elucidate the contextual factors influencing the community health workers programme, the authors ran stakeholder workshops and developed matrices of candidate theories as in Table 10, which outlines the tentative ‘context, mechanism and outcomes’ for the supply component of the community health worker programme. This paper helpfully outlines eight lessons learned by the authors of this realist evaluation.

Key points:

- The realist matrix enabled blending of context into the initial working programme theory, which in turn helped to map the relations between programme elements and identify evaluation measures to understand how, why, for whom and under what circumstances the intervention achieved its outcomes.
- Creating a programme theory is not the end – it is important to view programme theory as a flexible and iterative process to understand an intervention.
- Stakeholder engagement is crucial to ensure the development of a ‘shared language’ that is meaningful to a wider audience, not only researchers.

**Figure 7:** Dark logic model for Communities in Charge of Alcohol. Light blue arrows, potential links between harms; orange arrows, links between logic model inputs and outputs; black arrows, dark logic model mechanism; dark blue arrows, main logic model mechanism. AHC, Alcohol Health Champion. Reproduced with permission from Cook *et al*.^307^ This article is distributed under the terms of the Creative Commons Attribution 4.0 International License (http://creativecommons.org/licenses/by/4.0/), which permits unrestricted use, distribution, and reproduction in any medium, provided you give appropriate credit to the original author(s) and the source, provide a link to the Creative Commons license, and indicate if changes were made. The Creative Commons Public Domain Dedication waiver (http://creativecommons.org/publicdomain/zero/1.0/) applies to the data made available in this article, unless otherwise stated. The figure includes minor additions and formatting changes to the original figure.

**Table 10:** Matrix of context, mechanism and outcomes for the supply component of the community health worker programme

Levels of context	Levels of mechanisms	Levels of outcomes
Individual context:	Individual mechanism:	Individual outcome:
C1 – Non-experienced staff experience C2 – Demoralised staff C3 – Status and skill mix of MCH staff (CHWs, CHEWs and midwives)	M1 – Availability of health workers and skill mix of MCH staff ensured	O1 – Altruism and increased social responsibility O2 – Increased staff motivation O3 – Increased satisfaction O4 – Improved staff performance
Institutional context:	Institutional mechanism:	Institutional outcome:
C3 – Irregular salaries C4 – Poorly functioning facilities C5 – Strained working relationships between CHEWs and nurses following policy change in PHC facility management	M2 – Continuous training of staff M3 – Supportive supervision of staff M4 – Collegial working environment M5 – Regular payments are instituted M6 – Availability of equipment supplies and infrastructure	O5 – Increased staff retention O6 – Improved quality of care delivered by facility O7 – Increased utilisation of ANC by women O8 – Increased skilled birth attendance
Macro context:	Macro mechanism:	Macro outcome:
C6 – New government policy on social protection of vulnerable populations implemented as a pilot	M7 – Availability of SURE-P regulatory oversight	O9 – Reduced maternal mortality rate O10 – Reduced infant mortality rate

ANC, antenatal care/clinic; CHEW, community health extension worker; CHW, community health worker; MCH, maternal and child health; PHC, public health care; SURE-P, Subsidy Reinvention and Empowerment Programme. **Notes** From the above, we can start formulating hypotheses such as C1 + M1, M2, M4, M5 = O5, O6. Candidate theory 1: if different incentives (e.g. regular payments, training and improved working environment) are available in a timely manner, this will lead to improved and sustained health worker motivation, job satisfaction, performance and improved retention of staff in the context of Anambra State that is characterised by irregular salaries and poorly functioning facilities. Reproduced with permission from Ebenso *et al.*^308^ This is an Open Access article distributed in accordance with the terms of the Creative Commons Attribution (CC BY 4.0) license, which permits others to distribute, remix, adapt and build upon this work, for commercial use, provided the original work is properly cited. See: http://creativecommons.org/licenses/by/4.0/. The table includes minor additions and formatting changes to the original table.

### Case study 7: system map to develop programme theory

Study title: *Evaluation of the Health Impacts of the UK Treasury Soft Drinks Industry Levy (SDIL)*. *Project Protocol*.^224^

Summary: White *et al.^224^* took a systems approach to evaluate the UK’s SDIL: a graduated levy on the producers of sugar-sweetened drinks. The levy is intended to encourage the reformulation of drinks, with the aim of reducing sugar consumption and its associated health consequences. The researchers theorised the levy as an ‘event in a complex system’^224^ that might precipitate reactions among a wide array of stakeholders and affect a range of dietary and health outcomes. Anticipating direct and indirect effects across a range of interacting food and health systems, the researchers developed a bounded conceptual system map to articulate their understanding of how the intervention was expected to work, guide evaluation design and help identify data sources to support evaluation (Figure 8). The evaluation design includes multiple methods, for example qualitative process evaluation, natural experiment study, economic evaluation and simulation modelling of long-term (5–20 years) health and economic outcomes, with synthesis and triangulation of findings across methods planned.

**Figure 8:** Conceptual system map for the UK SDIL. Context: modifying factors (e.g. age, gender, socioeconomic position and household composition) and macro-level factors (e.g. Brexit, economic climate, agricultural policy, global governance and trade). Adapted with permission from White *et al*.^224^

Key points:

- A review of published evidence enabled the development of a preliminary conceptual map of the system and a theory for the SDIL.
- An interactive expert workshop refined the system map and verified hypothesised entities and their relationships, as well as the underlying programme theory.
- An online Delphi study built consensus among a wide range of stakeholders from all sectors and groups identified on the map, and enabled refinement of the map and programme theory.
- The final map allowed the generation of specific hypotheses concerning causal pathways central to the programme theory, the identification of potential data sources to measure outcomes associated with each key pathway and the design of a range of qualitative and quantitative studies to assess change in relation to these pathways.
- Synthesis of the findings from these studies will be used to revisit and refine the system map and programme theory, and derive generalisable causal inference.

### Case study 8: engaging stakeholders (service users)

Study title: *Reducing relapse and suicide in bipolar disorder: practical clinical approaches to identifying risk, reducing harm and engaging service users in planning and delivery of care* – *the PARADES* (*Psychoeducation*, *Anxiety*, *Relapse*, *Advance Directive Evaluation and Suicidality*) *programme*.^309^

Summary: this study evaluated approaches to reduce harm and improve outcomes for people with bipolar disorder. Stakeholder engagement, mainly in the form of service user involvement, was central throughout the duration of the Psychoeducation, Anxiety, Relapse, Advance Directive Evaluation and Suicidality (PARADES) programme, from the initial grant application to study close and dissemination. Service users were individuals with lived experience of bipolar disorder. ‘Involvement principles’ were agreed between service users and researchers at the outset of the programme and guided stakeholder engagement. Service users contributed to the PARADES programme in numerous ways, including delivering the intervention; offering insights on the duration and frequency of intervention sessions; practical issues, such as venue choices and accessibility; translating materials into culturally sensitive resources for participants; and helping to develop recruitment strategies. They also contributed to the interpretation and writing up of findings and the dissemination strategy. At the end of the study, they developed a user-friendly guide to the Mental Capacity Act^310^ for people with bipolar disorder.

Key points:

- PPI activities in this study were wide-ranging and central throughout the project.
- Involving service users with lived experiences of bipolar disorder had many benefits for the research. It enhanced the intervention but also improved the evaluation and dissemination methods.
- The benefits were two-way because there were positive outcomes for service users involved in the study, including more settled employment, progression to postgraduate study, fellowship funding and co-authorship of several papers.

### Case study 9: refining the intervention

Study title: *The person-based approach to enhancing the acceptability and feasibility of interventions*.^311^

Summary: this paper describes using mixed-methods approaches to develop an in-depth understanding of the needs and views of people who will use the intervention. This understanding ‘enables intervention developers to design or modify the intervention to make it more relevant, persuasive, accessible and engaging’.^311^ Three illustrative examples are used to demonstrate the iterative process:

- Illustrative example 1 involved developing guiding principles that informed the design of a digital intervention for asthma self-management. Guiding principles were developed based on existing evidence and relevant issues that the intervention would need to address. The process involved developing key intervention design objectives to address identified issues, and intervention features were developed to address these design objectives.
- Illustrative example 2 used think-aloud interviews, alongside PPI input, to improve the intervention booklet. This involved changing the images and wording of the booklet several times, leading to a more acceptable resource for participants. This refinement process is likely to lead to higher engagement with the booklet.
- Illustrative example 3 also used think-aloud interviews to improve the usability of a physical activity planner for people with diabetes mellitus. In this example, a physical activity planner was found to be difficult to use, resulting in participants overestimating their level of physical activity. Several iterations of the planner were developed based on interviews and observations and this iterative process led to the planner being much more feasible and accurate.

Key points:

- The feasibility and acceptability of these interventions was improved by involving potential users to inform intervention refinements.
- This process of iterative intervention refinement increases the chances that the interventions will be acceptable to participants and feasible to deliver.
- It is also likely that this refinement process will increase the chances of the interventions being found to be effective in a future evaluation.

### Case study 10: economic considerations

Study title: Adaptation of the New Orleans Intervention Model (NIM) for infants entering foster care – the Best Services Trial (BeST?) in Scotland, UK.^265,312–318^

Summary: the New Orleans Intervention Model (NIM) for infants entering foster care seeks to improve the quality of placement decisions while also improving the mental health outcomes for these infants. The intervention is set within a complex context, spanning social care and the NHS, in which the comparator (service as usual) varies in service delivery across the UK. The development and testing of the intervention within a UK context has been conducted in an iterative manner,^312^ with the inclusion of economic considerations from the outset.^313^

An early-stage exploratory economic model was developed comparing the proposed NIM intervention with the existing local foster care service. A full economic evaluation was deemed inappropriate at this stage, as the existing foster care service was not clearly defined and there was no direct clinical evidence on health outcomes in this or the proposed new system. Instead, a CCA framework was used to map and measure existing local services and compare them with a hypothetical model of the NIM intervention. This allowed decision-makers to compare the potential costs and consequences of these two approaches and form their own view of the importance of the different outcomes.^141^

Evidence from the early-stage model helped to build a case for a Phase II exploratory trial in Glasgow,^314–317^ including development and feasibility testing of economic resource use data systems. The feasibility study led to a definitive Phase III trial^318^ that included short- and long-term economic analysis from multiple perspectives;^265^ included NHS and Personal Social Services, public sector and wider societal perspectives; and used a range of frameworks, including CUA and CCA to capture changes in the intersectoral costs and outcomes associated with child maltreatment.

Key points:

- An iterative approach was taken for the design and conduct of the economic evaluation.
- The evaluation took into account outcomes spanning a range of sectors; short-term, intermediate and long-term time horizons; and evidence from a range of sources.
- The use of multiple economic evaluation frameworks will provide decision-makers with a comprehensive, multiperspective guide to the cost-effectiveness of the NIM.

### Case study 11: intervention development: example systems map

Study title: *Tackling ‘wicked’ health promotion problems: a New Zealand case study*.^319^

Summary: this study describes a complex environmental approach to addressing ‘wicked’ health promotion problems. The starting point for this research was the problem – obesity, poor nutrition and inadequate levels of physical activity among Māori, Pacific and low-income people in New Zealand – rather than a particular intervention. The project involved literature reviews, focus groups, stakeholder workshops and key informant interviews to identify important areas for intervention and provide recommendations for action. The system was mapped (Figure 9) and analysed, identifying ‘control parameters’, ‘elements of a system that have a controlling influence on other system elements, acting to set the boundaries within which the system can operate’,^319–322^ that are open to manipulation. Stakeholder workshops explored the options for interventions to affect change on the control parameters, and intervention areas were then prioritised.

Key points:

- The research took a systems perspective that aimed to inform system-level change.
- Mapping of the relevant systems facilitated the identification of a suite of interventions that were likely to be effective, taking into account feasibility, acceptability, sustainability, equity, side effects and costs.
- A collaborative strategy for intervention development was used, with effort taken to include relevant multidisciplinary stakeholders, including community members and policy-makers.
- A mixed-methods approach was taken utilising data from multiple sources.
- Cost–benefit in terms of intervention options was considered from the outset to ensure value for money.

### Case study 12: feasibility trial

Study title: *Randomized feasibility trial of directly observed versus unobserved hepatitis C treatment with ledipasvir-sofosbuvir among people who inject drugs*.^323^

Summary: this study explored the feasibility of an intervention to reduce secondary transmission of the hepatitis C virus in people who inject drugs. The uncertainties identified at the start of the study included the feasibility of treating people at high risk for secondary transmission and the optimal approach for delivering treatment. The study also explored the feasibility of recruitment, adherence to the intervention, retention in the study and potential harms. The findings demonstrated that both modes of delivery were feasible and acceptable to participants. It was also feasible to recruit people who inject drugs for this type of treatment, retain them over a 12-week follow-up period and achieve positive viral responses to treatment.

Key points:

- Uncertainties were identified before the study commenced, which guided the choice of intervention and evaluation methods.
- The feasibility and acceptability of the intervention and evaluation methods were tested.
- Useful data were gathered to optimise the design of a future evaluation.
- The findings addressed the initial uncertainties, thereby strengthening the support for future funding and/or evaluation.

### Case study 13: feasibility, evaluability assessment

Study title: *Practice to evidence: using evaluability assessment to generate practice-based evidence in rural South Georgia*.^324^

**Figure 9:** Physical activity system map with interventions. Reproduced from Signal LN, Walton MD, Mhurchu CN, *et al*. Tackling ‘wicked’ health promotion problems: a New Zealand case study, *Health Promotion International* 2013;**28**(1):84–94,^319^ by permission of Oxford University Press.

Summary: the Emory Prevention Research Centre used evaluability assessment methods as part of an initiative to gather practice-based evidence on cancer prevention from community interventions in south-west Georgia, USA. Of nine interventions that were identified and screened for eligibility, two were selected for a full evaluability assessment. Each evaluability assessment comprised a review of documents related to the intervention, site visits, meetings with stakeholders and reviews of published literature. The aim was to assess the feasibility and usefulness of an evaluation based on the programme theory underpinning the intervention, the availability of data, stakeholder interest and its potential contribution to the evidence base. From the two evaluability assessments, one intervention was identified as eligible for a quasi-experimental outcome evaluation. Data limitations precluded an outcome evaluation of the second intervention and a recommendation was made instead for either a process evaluation of one component of the programme or a descriptive case study.

Key points:

- In the context of scarce evaluation resources, partnerships between research and practice can helpfully identify interventions that can generate useful findings and inform future implementation.
- Interventions were screened from current practice and evaluability assessments used to assess those with the potential for formal evaluation and to recommend an evaluation method.
- Findings from the initiative described were included in national resources for practitioners looking at evidence-based practices and helped the community organisations expand and strengthen their programmes.

### Case study 14: evaluation (effectiveness perspective: cluster randomised controlled trial with theory-informed process evaluation)

Study title: *Management of multimorbidity using a patient-centred care model: a pragmatic cluster-randomised trial of the 3D approach*.^177^

Summary: this pragmatic cluster RCT is an effectiveness study of the 3D intervention, a primary care approach for patients with multimorbidity, which focuses on quality of life and self-management to achieve goals agreed between the patient and the health-care provider. The intervention aimed to improve health-related quality of life. Process and cost-effectiveness evaluations were also completed. There was no difference in quality of life between intervention and control groups.

Key points:

- Cluster RCTs are useful when an intervention is aimed at communities rather than individuals, and also where there is potential for treatment contamination, for example where control participants may gain access to intervention materials. However, they are more complex to design and analyse and increase the sample size requirements.
- The trial process evaluation sought to understand how and why the intervention was effective (or not) and to identify strategies for implementation that considered the influence of context, as well as practical issues related to adoption, delivery maintenance and reach. Examples of research questions were ‘to what extent was the intended intervention delivered to patients?’^177^ and ‘how did patients perceive the intervention and its delivery?’.^177^
- The interpretation of findings was enhanced by the nested qualitative process evaluation that followed a prespecified protocol.^190,325^
- An external pilot of the study was conducted before proceeding to this trial.
- A study protocol was written, published and followed.^326^ This included a prespecified analysis plan for primary and secondary outcomes.
- Economic evaluation of cost consequences and cost-effectiveness was conducted alongside the trial.^327^

### Case study 15: evaluation (efficacy)

Study title: *Beneficial effect of carotid endarterectomy in symptomatic patients with high-grade carotid stenosis*.^174^

Summary: this RCT in 50 centres tested, among 659 patients with a high risk of stroke (70–99% stenosis of a carotid artery), whether or not the addition of carotid endarterectomy to optimal medical therapy (compared with optimal medical therapy alone) reduces the risk of stroke. The trial results indicated that carotid endarterectomy was beneficial.

#### Key points

This efficacy trial can be differentiated from an effectiveness trial based on the following characteristics^259^–

- The inclusion criteria for the trial were narrow. The main group of interest were those who were at high risk of stroke and those who were most likely to respond to the intervention. The exclusion criteria were wide-ranging; therefore, the intervention was tested on a very narrow group of patients.
- The intervention delivery was tightly constrained with little flexibility, and those delivering the intervention were highly trained. The surgeons taking part had to be approved by an expert panel and had to have completed at least 50 carotid endarterectomies in the last 24 months.
- The control intervention was not treatment as usual but included antiplatelet therapy and the same co-interventions applied to the intervention arm were also given to control patients.
- Follow-up was very intensive at multiple time points.
- Practitioner adherence to the study protocol was monitored.
- In real-world practice, much of the above would not happen; therefore, the impact of the intervention is likely to be different in a more pragmatic setting.

### Case study 16: evaluation (effectiveness perspective: individual randomised controlled trial)

Study title: *Exercise- and strategy-based physiotherapy-delivered intervention for preventing repeat falls in people with Parkinson’s: the PDSAFE RCT*.^175^

Summary: people with Parkinson’s disease are at an increased risk of falls. Although there is some evidence that exercise might reduce fall risk, the findings are mixed. This study examined the clinical effectiveness and cost-effectiveness of PDSAFE, an exercise- and strategy-based intervention for fall reduction for people with Parkinson’s disease. The study was a multicentre, individual RCT with an embedded economic evaluation and qualitative process evaluation. A published protocol included prespecified subgroup analyses. This was useful because, although the intervention was not effective overall in terms of reducing fall risk, these analyses indicated that it may be of benefit for those with ‘moderate’ Parkinson’s disease, but it may increase falls in those with more severe Parkinson’s disease. The authors concluded, therefore, that a different treatment strategy could be used for those with more severe Parkinson’s disease and that the benefits indicated for those with ‘moderate’ Parkinson’s disease would need to be further studied in an effectiveness trial for this group before implementation.

Key points:

- This trial tested the intervention in those patients who are representative of those likely to receive the intervention in routine practice.
- The qualitative process evaluation provided information on barriers to and potential facilitators of participation (e.g. existing social support). These aspects could be incorporated to further enhance this intervention for more targeted groups, for whom the intervention is more likely to be effective.
- An economic evaluation was conducted as part of the trial and included subgroup analyses.
- Engagement with stakeholders, including professionals and patients, supported the development of some of the study materials.
- In line with good reporting practice, CONSORT and Template for Intervention Description and Replication (TIDIER) descriptions were provided.

### Case study 17: evaluation (effectiveness perspective: stepped-wedged cluster randomised design)

Study title: *Effect of the World Health Organization (WHO) Checklist on Patient Outcomes: a stepped wedge cluster randomized controlled trial*.^178^

Summary: this stepped-wedge cluster RCT examined the effects of implementing WHO’s surgical safety checklist on in-hospital complications, length of stay and mortality. The stepped-wedge design was used because the surgical safety checklist intervention was thought more likely to do good than harm; therefore, it would be unethical to not deliver or to withdraw the intervention. The surgical safety checklist was randomly rolled out in sequence until five clusters had received the intervention. The different clusters (surgical specialties) switched treatment from control (no checklist) to intervention (surgical safety checklist) at different time points. The clusters crossed over in one direction, that is the checklist was introduced and not withdrawn after implementation. The surgical specialty cohorts acted as their own controls providing data before and after the checklist intervention. The analyses compared all data in the steps before (controls) with the steps after (intervention) the intervention. The results indicated that use of the surgical safety checklist led to important reductions in morbidity and length of hospital stay.

Key points:

- The stepped-wedge cluster randomised design is especially useful where (1) it would be unethical to not deliver or to withdraw the intervention; (2) randomising individuals or procedures would not be possible; (3) contamination is likely to be a major problem; (4) there are practical or financial constraints that make it impossible to deliver the intervention to all participants at the same time.
- Issues with the stepped-wedge design are that it can require a large number of data. In this example, routinely collected data were used to address this issue. It can also be difficult to implement the interventions within the planned time frame because of the practicalities of working within complex health-care organisations.

### Case study 18: evaluation (effectiveness perspective: synthetic control)

Study title: *Examination of the synthetic control method for evaluating health policies with multiple treated units*.^179^

Summary: The Advancing Quality scheme (a hospital pay-for-performance scheme) was re-evaluated using the synthetic control method and the findings were contrasted to the original differences-in-differences analysis (in which the main assumption is parallel trends). The original differences-in-differences study compared intervention hospitals with hospitals that did not introduce the Advanced Quality scheme, and found that risk-adjusted hospital mortality was reduced for the three incentivised conditions.^328^ Concerns were raised about whether or not the parallel-trends assumption was upheld given an inspection of risk-adjusted mortality in the periods before the introduction of the Advanced Quality scheme. The data were, therefore, re-evaluated using the synthetic control method, which allows for estimation of intervention effects by constructing a weighted combination of control units. This represents an estimate of what the intervention group would have experienced in the absence of the intervention; see the full paper for the methods.^179^ Results did not align with those from the original differences-in-differences analysis, but rather found that the scheme did not reduce mortality for incentivised conditions and that mortality was significantly increased in non-incentivised conditions. Sensitivity analysis upheld findings from the synthetic control method.

Key points:

- Many public health interventions cannot be evaluated using RCTs and rely on the assessment of observational data.^215^ Building synthetic controls, using a weighted combination of potential control units, is one way of overcoming any difficulties in selecting available, suitable controls.
- Where the validity of the parallel-trends assumption is doubtful, the synthetic control method may be useful. This method allows for the effects of unobserved confounders to change over time by weighting the control group so that it has similar pre-intervention characteristics to the intervention group.^329^
- This study adds to concerns that the parallel-trends assumption underlying the differences-in-differences approach may not be plausible in settings where the differences between the comparison groups in pre-intervention outcomes are relatively large, showing the appropriate use of a synthetic control approach.

### Case study 19: development and evaluation – effectiveness perspective (drawing on a systems perspective for intervention development and evaluation)

Study title: *Whole of Systems Trial of Prevention Strategies for childhood obesity: WHO STOPS childhood obesity*.^180^

Summary: this paper presents the protocol for a stepped-wedge cluster RCT evaluating WHO STOPS, a complex intervention targeting childhood obesity that was developed using a systems perspective. The intervention engages and facilitates community members to agree a system map of causes of childhood obesity for the community; ‘identifies intervention opportunities through leveraging the dynamic aspects of the system’;^180^ and uses these to co-produce systems-oriented action plans that are implemented in a way that allows flexible and iterative development of the intervention over time. Pilot work undertaken with communities using group model building to build causal loop diagrams indicated that participants were able ‘to identify multiple systems that impact childhood obesity’^180^ and ‘design interventions that considered non-linearity, feedback, and complexity for intervention development’;^180^ see Allender *et al*.^330^ for a worked example of a causal loop diagram. The evaluation will test the impact of the intervention on childhood obesity prevalence. Ten communities will be randomised: five to receive the intervention at year 1 and five to receive the intervention at year 3. The evaluation also draws on a systems perspective and includes numerous systems methods to explore the impact of the intervention at a systems level.

Key points:

- The study involves stakeholder engagement and considers implementation and context from the outset.
- Systems boundaries will be predefined and agreed by a range of community stakeholders.
- Systems change will be assessed alongside the primary outcome (childhood obesity) using a variety of methods, for example using social network analysis.
- A dynamic logic model will be created and updated throughout.
- Economic evaluation is included in the trial, using approaches that are suitable to capture systems change as well as equity impacts.
- Qualitative methods, largely drawing on soft systems methodology (e.g. group model-building workshops),^331^ and quantitative methods will be employed throughout, and the process evaluation will assess the response of the community to the systems intervention.

### Case study 20: evaluation (theory-based perspective, alongside effectiveness)

Study title: *A realist process evaluation of robot-assisted surgery: integration into routine practice and impacts on communication, collaboration and decision-making*.^182^

Summary: robot-assisted surgery has been shown to have potential benefits for patients; however, despite the technology being available, this has not yet fully been implemented into routine practice in the NHS. This realist evaluation aimed to explore ‘how and under what circumstances robot-assisted surgery is effectively introduced into routine practice, and how and under what circumstances robot-assisted surgery impacts communication, teamwork and decision-making’.^182^ This was done alongside an effectiveness trial of robot-assisted surgery.^332,333^

Phase 1 comprised a literature review that explored stakeholder theories of how robot-assisted surgery can become embedded into routine practice, and particularly how it affects communication, decision-making and teamwork. The theories were further developed using data from stakeholder interviews. In phase 2, four theories were selected from the initial theories developed in phase 1 for testing in a multisite case study across four NHS trusts. This involved multiple methods, such as video recordings of operations, ethnographic observation, post-operation interviews and a questionnaire. In phase 3, interviews with surgical staff from other specialties were completed to explore whether or not the results of phase 2 were generalisable and to refine the theories to include the experience of a wider group of surgical specialties.

Key points:

- A theory-based evaluation can usefully be carried out alongside an effectiveness trial to understand the variations in implementation of the intervention and the influence of context on outcomes.
- Multiple methods can be used to investigate how and under what circumstances an intervention affects processes related to the implementation of an intervention.
- Data collection and analysis took an iterative approach meaning that refinement of theories was ongoing, and further data could be gathered as necessary.
- Theory-based evaluation can provide standalone findings, as well as complement an effectiveness evaluation.
- The study resulted in several practical recommendations to support the implementation of robot-assisted surgery into routine surgical practice. For example, engaging staff at different levels, including surgeons who will not be using robot-assisted surgery, whole-team training and a suitably sized operating theatre.

### Case study 21: evaluation (theory-based perspective with realist approach)

Study title: *‘Patients are not following the [adherence] club rules anymore’: a realist case study of the antiretroviral treatment adherence club, South Africa*.^183^

Summary: effectiveness studies have shown positive results of the adherence club intervention model, an antiretroviral treatment delivery model, in terms of improved retention in care and adherence to antiretroviral treatment for people living with human immunodeficiency virus (HIV). However, understanding how, why and under what health system contexts the intervention could be most effective were not clear. The aim of this realist evaluation was to test the initial programme theory and to ‘determine how, why, for whom, and under what health system context the adherence club intervention works (or not) in real-life implementation’.^183^ The study identified certain mitigating circumstances that could inform implementation of the intervention.

Key points:

- The evaluation was transparent, in that a prespecified plan for the research was set out in a published protocol paper.^334^
- Mixed methods were used within a case study approach. Qualitative methods were used ‘to explore implementation features related to the context and mechanisms, and quantitative methods to describe and classify the outcomes’.^183^
- Data from various participants (health-care providers and patients) were triangulated to verify what was obtained.
- An explanatory tool [Intervention-Context-Actor-Mechanism-Outcome (ICAMO; intervention–context–actor–mechanism–outcome)] was used to construct theories, building on realist context – mechanism – outcome configurations, to explore how the ‘outcome (O) is generated by a mechanism (M), being triggered in context (C), through an actor (A), when the intervention is implemented (I)’.^183^
- Links between each element of ICAMO were conceptualised and then compared and contrasted with the initial programme theory. A transparent and systematic process was followed.
- The initial programme theory was modified to reflect findings, providing an updated explanation of how, why, for whom and under what health system conditions the adherence club intervention works (or not).
- Guidelines for reporting realist evaluation studies were followed.^255^

### Case study 22: evaluation (theory-based approach)

Study title: *The impact on health inequalities of approaches to community engagement in the New Deal for Communities regeneration initiative: a mixed-methods evaluation*.^184^

Summary: this study drew on a theory of change approach to evaluate the New Deal for Communities, thus exploring how and why the intervention ‘works’. It explored the social and health impacts of different community engagement approaches that were undertaken as part of the New Deal for Communities regeneration initiative in England. There were 39 local New Deal for Communities programmes that were each given a budget to engage residents in planning and implementing initiatives to reduce crime and worklessness, and improve community cohesion, housing, the physical environment, education and health. The study used primary and secondary data, underpinned by theories of engagement and empowerment, to categorise the community engagement approach of each programme into four types and explore community empowerment as a mechanism of change within these approaches. Analysis explored how the four community engagement types related to different health and social outcomes, ultimately looking at whether or not the different community engagement approaches explained any of the differential outcomes of the New Deal for Communities programmes (that were previously identified). Economic analysis was also undertaken to explore the cost-effectiveness of different community engagement approaches. Statistical results were mixed and most were not statistically significant, and evidence of effectiveness was inconclusive because of limited accurate cost data. However, patterns of change were consistent with theories about the relationship between empowerment and health and social outcomes. The theoretical interpretation highlighted which of the different community engagement approaches could be problematic and may lead to unintended negative health and social outcomes, and which could more plausibly have positive impacts and, thus, how community engagement approaches could be made to work better. The findings highlight the need for prudent application of theory to make sense of effectiveness studies, in this case where results are inconclusive.

Key points:

- The typology of community engagement had strong theoretical underpinnings and was sense checked with the New Deal for Communities residents and practitioners, who were also advisors in the study.
- Theory of change was explored by testing plausible programme theories in practice.
- The value of this study lies in providing an understanding of the mechanisms of change and relationship with context, rather than the effect estimate.

### Case study 23: evaluation (systems perspective)

Study title: *Evaluating a complex health promotion intervention: case application of three systems methods*.^185^

Summary: the Delaware Young Health Program IM40 was an initiative to address the health and well-being of young people in Delaware through a developmental assets approach. It was funded by AstraZeneca plc (Cambridge, UK), a pharmaceutical company. By drawing on systems thinking and complexity science, the study aimed to identify and examine underlying patterns and structures in the network of individuals and their relationships that influenced system-wide behaviours, for example a culture shift in communities to better value young people. IM40 was a complex intervention in the sense that it sought to influence several levels simultaneously (individual, professional, procedural and policy) and it was flexible and adaptive. Participating local communities were expected to follow a set of principles rather than adopt particular strategies. Each community had a different starting point in terms of relationships between organisations and existing strategies. Three systems science methods were used: group model building and viable systems model assessment to identify underlying patterns and structures, and social network analysis to evaluate change in relationships over time.

Key points:

- IM40 was expected to change underlying system dynamics, structures and conditions; therefore, the evaluation identified systems-oriented research questions and methods.
- The systems-oriented research questions were checked with stakeholders to ensure that they were relevant and useful. The study used mixed methods to gather data.
- A developmental evaluation focus was taken, which was appropriate to the complexity involved and the early stage of the work.
- Group model building was used to collaboratively create a causal model of the intervention with key stakeholders. The viable systems model method was used to model and assess the emergent organisational structure of the intervention. Social network analysis was conducted to explore shifts in the relationships between actors in the system. Strengths and limitations of each method used are reported in the paper.

### Case study 24: evaluation (systems perspective with process evaluation)

Study title: *Consequences of removing cheap, super-strength beer and cider: a qualitative study of a UK local alcohol availability intervention*.^186,187^

Summary: ‘Reducing the Strength’ is an intervention in which local authorities ask retailers to stop selling ‘super-strength’ beer and cider. Street and homeless drinkers are the target population for the intervention. The mixed-methods process evaluation took a systems perspective to explore the pathways through which the intervention may lead to intended and unintended consequences. The authors interviewed relevant stakeholders including compliers and non-compliers (shop keepers), the target public (homeless people) and stakeholders from a wider array of relevant services (homeless service providers relevant to alcohol and accommodation provision, and community safety officers). This was carried out to determine whether or not the intervention was developed to fit coherently within the wider system and to look at the consequences of different forms of adaptation and feedback, for example finding that homeless people adapt by changing their method of intoxication or by switching to shops that do not comply with the intervention. The authors conclude that owing to the above issues and the small-scale implementation and limited range of products included in Reducing the Strength, the intervention can make only a modest impact on alcohol harms. However, the intervention may lead to wider system changes as it impacts on services not just individual drinkers.

Key points:

- Reducing the Strength was conceptualised as an event in a complex system, and the way in which multiple people or agents within the system responded to it was explored.
- Multiple perspectives were sought.
- The study drew on complexity theory and developed multilevel theories of change to explain the impacts of the intervention.
- The qualitative work identified how the Reducing the Strength intervention affected components of the system and teased out the mechanisms by which the intervention triggered system changes.
- Qualitative methods may help address barriers to evaluating local health policy innovation, ‘which can include small delivery scales, rapid delivery timescales, and a demand from local decision makers for evidence that is sufficiently contextually rich to be recognisable to them as local’.^186^

### Case study 25: evaluation (systems perspective with qualitative comparative analysis)

Study title: *A qualitative comparative analysis of factors associated with trends in narrowing health inequalities in England*.^188^

Summary: targets for reducing health inequalities in England were adopted by the UK Government in 2001. Seventy ‘spearhead’ local authority areas were identified (those in the bottom fifth for five factors related to mortality, morbidity and the Index of Multiple Deprivation: 28% of the population in England). An example target was that the difference in life expectancy between the English and the spearhead areas average should reduce by 10%. The study aimed to explore variation between areas on their progress in achieving the targets, particularly looking at cardiovascular disease and cancer outcomes. QCA was used to explain why particular ‘cases’ have particular outcomes. Each spearhead area was a case in the analysis and each case was thought of as a local system, with different organisational attributes and contextual features. Data were gathered about the local conditions of each case (at present time and 3 years previous) using questionnaires completed by relevant professionals in each area. Secondary data were also used, for example hospital admissions and crime rates. The fsQCA software (version 3.0; Ragin and Davey, Irvine, CA, USA) was used to explore the effects of the attributes on the outcome indicators in combination with each other rather than individually. This showed the different configurations of attributes, for example the combination of ‘individual commitment and champions’ and ‘higher spend per head on cancer programmes’, that were associated with narrowing/widening inequalities between spearhead areas and English averages. Necessary and sufficient conditions were then identified for each morbidity and mortality outcome; for example, for narrowing the cancer gap, ‘championing’ was present in all configurations and was absent where the cancer gap was not narrowing. Results of the study were presented at a series of local stakeholder workshops, and to understand more about the sufficient conditions identified, such as individual commitment and championing, the authors planned to have discussions with local stakeholders.

Key points:

- QCA focuses on inter-relations between the system’s constituent parts rather than on the individual components.
- Substantive and theoretical reasoning, rather than experimental design, is used to justify causal pathways.
- Anomalous cases are used as triggers for further investigation.
- Stakeholder involvement is key to the QCA process, both for defining and measuring important attributes and for interpreting the results.

### Case study 26: intervention development – discrete choice experiments

Study title: *Using discrete choice experiments to inform the design of complex interventions*.^262^

Summary: discrete choice experiments can be used to quantify user preferences for different aspects of an intervention and to explore heterogeneity in preferences among users. This study used a discrete choice experiment in combination with a qualitative study to develop a voluntary medical male circumcision intervention for HIV infection prevention in the United Republic of Tanzania. Qualitative data from interviews and participatory group discussions with women, young men (< 20 years) and older men (≥ 20 years) were used to identify barriers to and facilitators of voluntary medical male circumcision, such as the role of female partners, service providers’ attitudes and social stigma. A discrete choice experiment (n = 325) was used to measure men’s preferences for or against characteristics of voluntary medical male circumcision services, such as availability of partner counselling, age-separated waiting areas and female health-care staff. The findings were used to develop an intervention that included community information booths, separate waiting areas and training for providers on client friendliness.

Key points:

- Qualitative and quantitative data were combined to develop an intervention to inform the development of a complex intervention.
- Participant preferences are an important determinant of uptake and adherence to an intervention, such as voluntary medical male circumcision, given the stigma associated with HIV.
- The use of a discrete choice experiment, informed by qualitative information about barriers to and facilitators of, enabled the researchers to design an intervention aligned with user preferences.

### Case study 27: implementation in all phases

Study title: An *informal school-based peer-led intervention for smoking prevention in adolescence (ASSIST): a cluster randomised trial*.^146^

Summary: the ASSIST programme identified the most influential students in schools from the 12- to 13-year age group, who were then trained to act as peer supporters during informal interactions outside the classroom to encourage their peers not to smoke. The intervention was based on diffusion of innovation theory and adapted an effective adult-focused sexual health programme. It was developed in collaboration with health and education professionals, and the intervention and evaluation methods were assessed in a feasibility study. A cluster randomised effectiveness trial in 59 schools with nested process and economic evaluations found the intervention to be cost-effective. Key contextual factors, intervention mechanisms and variations in implementation were identified.^335–337^

Key points:

- The intervention was designed to have minimal dependence on school resources, and to avoid poor implementation, often identified as a key problem of smoking education in real-world classrooms.
- A feasibility study identified a number of changes to the prototype programme to improve its acceptability to students and schools and to improve the reach of the programme to male students.
- In the evaluation phase cluster randomised trial, the intervention was delivered as closely as possible to a real-world implementation. Process evaluation data and the programme theory were used to identify the critical components of the intervention and key barriers to and facilitators of delivery.
- Programme implementation involved an intervention manual that identified both critical components and adaptable components, allowing for flexible implementation while retaining the key mechanisms of change. A training manual for the trainers and ongoing quality assurance were built into longer-term roll-out, based on the programme theory and process evaluation findings.

## Supplementary Material

Appendix

## Data Availability

Owing to this project being methodological in nature there is no data that can be shared. For more information please contact the corresponding author.

## List of abbreviations

ABM: agent-based modelling
ASSIST: A Stop Smoking in Schools Trial
CBA: cost-benefit analysis
CCA: cost-consequence analysis
CEA: cost-effectiveness analysis
CEEBIT: Continuous Evaluation of Evolving Behavioural Intervention Technologies
COMET: Core Outcome Measures in Effectiveness Trials
CONSORT: Consolidated Standards of Reporting Trials
CUA: cost-utility analysis
DELTA2: Difference ELicitation in TriAls
FFIT: Football Fans in Training
GP: general practitioner
HIV: human immunodeficiency virus
ICAMO: intervention–context–actor–mechanism–outcome
MCDA: multi-criteria decision analysis
MRC: Medical Research Council
MRP: Methodology Research Programme
NICE: National Institute for Health and Care Excellence
NIHR: National Institute for Health Research
NIM: New Orleans Intervention Model
PARADES: Psychoeducation, Anxiety, Relapse, Advance Directive Evaluation and Suicidality
PHSG: Population Health Sciences Group
PPI: patient and public involvement
PRECIS: PRagmatic Explanatory Continuum Indicator Summary
QALY: quality-adjusted life-year
QCA: qualitative comparative analysis
RCT: randomised controlled trial
SAG: Scientific Advisory Group
SDIL: Soft Drinks Industry Levy
SMART: Sequential Multiple Assignment Randomised Trial
VOI: value of information
WHO: World Health Organization

## References

[1] Craig P, Dieppe P, Macintyre S, Michie S, Nazareth I, Petticrew M (2006). Developing and Evaluating Complex Interventions.

[2] Medical Research Council (MRC) (2000). A Framework for Development and Evaluation of RCTs for Complex Interventions to Improve Health.

[3] Campbell M, Fitzpatrick R, Haines A, Kinmonth AL, Sandercock P, Spiegelhalter D, Tyrer P (2000). Framework for design and evaluation of complex interventions to improve health. BMJ.

[4] Craig P, Dieppe P, Macintyre S, Michie S, Nazareth I, Petticrew M, Medical Research Council Guidance (2008). Developing and evaluating complex interventions: the new Medical Research Council guidance. BMJ.

[5] Moore G, Audrey S, Barker M, Bond L, Bonell C, Hardeman W (2014). Process Evaluation of Complex Interventions Medical Research Council Guidance.

[6] Raine R, Fitzpatrick R, Barratt H, Bevan G, Black N, Boaden R (2016). Challenges, solutions and future directions in the evaluation of service innovations in health care and public health. Health Serv Deliv Res.

[7] Rutter H, Savona N, Glonti K, Bibby J, Cummins S, Finegood DT (2017). The need for a complex systems model of evidence for public health. Lancet.

[8] Greenhalgh T, Papoutsi C (2018). Studying complexity in health services research: desperately seeking an overdue paradigm shift. BMC Med.

[9] Craig P, Cooper C, Gunnell D, Haw S, Lawson K, Macintyre S (2012). Using Natural Experiments to Evaluate Population Health Interventions: Guidance for Producers and Users of Evidence.

[10] Craig P, Di Ruggiero E, Frolich KL, Mykhalovskiy E, White M, on behalf of the Canadian Institutes of Health Research (CIHR)–National Institute for Health Research (NIHR) Context Guidance Authors Group (2018). Taking Account of Context in Population Health Intervention Research: Guidance for Producers, Users and Funders of Research.

[11] O’Cathain A, Croot L, Duncan E, Rousseau N, Sworn K, Turner KM (2019). Guidance on how to develop complex interventions to improve health and healthcare. BMJ Open.

[12] University of Glasgow Guidance of Explanatory Studies of Complex Public Health Interventions (The GUEST Study).

[13] Egan M, McGill E, Anderson de Cuevas R, Er V, Lock K, Popay J (2019). NIHR SPHR Guidance on Systems Approaches to Local Public Health Evaluation Part 1: Introducing Systems Thinking.

[14] Egan M, McGill E, Penney T, Anderson de Cuevas R, Er V, Orton L (2019). NIHR SPHR Guidance on Systems Approaches to Local Public Health Evaluation Part 2: What to Consider When Planning a Systems Evaluation.

[15] De Silva MJ, Breuer E, Lee L, Asher L, Chowdhary N, Lund C, Patel V (2014). Theory of change: a theory-driven approach to enhance the Medical Research Council’s framework for complex interventions. Trials.

[16] French SD, Green SE, O’Connor DA, McKenzie JE, Francis JJ, Michie S (2012). Developing theory-informed behaviour change interventions to implement evidence into practice: a systematic approach using the Theoretical Domains Framework. Implement Sci.

[17] Wight D, Wimbush E, Jepson R, Doi L (2016). Six steps in quality intervention development (6SQuID). J Epidemiol Community Health.

[18] Hawkins J, Madden K, Fletcher A, Midgley L, Grant A, Cox G (2017). Development of a framework for the co-production and prototyping of public health interventions. BMC Public Health.

[19] Collins LM, Murphy SA, Strecher V (2007). The multiphase optimization strategy (MOST) and the sequential multiple assignment randomized trial (SMART): new methods for more potent eHealth interventions. Am J Prev Med.

[20] Effective Practice and Organisation of Care (2015). Effective Practice and Organisation of Care (EPOC) Taxonomy.

[21] Campbell M, Moore G, Evans RE, Khodyakov D, Craig P, on behalf of the ADAPT Study team (2020). ADAPT study: adaptation of evidence-informed complex population health interventions for implementation and/or re-evaluation in new contexts: protocol for a Delphi consensus exercise to develop guidance. BMJ Open.

[22] Chalmers I, Bracken MB, Djulbegovic B, Garattini S, Grant J, Gülmezoglu AM (2014). How to increase value and reduce waste when research priorities are set. Lancet.

[23] HM Treasury (2018). The Green Book Central Government Guidance on Appraisal and Evaluation.

[24] Hawe P, Shiell A, Riley T (2009). Theorising interventions as events in systems. Am J Community Psychol.

[25] Petticrew M (2011). When are complex interventions ‘complex’? When are simple interventions ‘simple’?. Europ J Public Health.

[26] Skivington K, Matthews L, Simpson SA, Craig P, Baird J, Blazeby J (2021). A new framework for developing and evaluating complex interventions: update of Medical Research Council guidance. BMJ.

[27] Mercer S, Wyke S, Fitzpatrick B, McConnachie A, O’Donnell K, Mackenzie M (2017). *Evaluation of the Glasgow* ‘Deep End’ *Links Worker Programme*.

[28] Charles CV, Dewey CE, Hall A, Hak C, Channary S, Summerlee A (2015). A randomized control trial using a fish-shaped iron ingot for the amelioration of iron deficiency anemia in rural Cambodian women. Trop Med Surg.

[29] Charles CV, Dewey CE, Daniell WE, Summerlee AJ (2011). Iron-deficiency anaemia in rural Cambodia: community trial of a novel iron supplementation technique. Eur J Public Health.

[30] Press Release: Iron Fish Update on Clinical Trial in Preah Vihear, Cambodia.

[31] Meadows DH, Wright D (2008). Thinking in Systems: A Primer.

[32] The Health Foundation (2010). Evidence Scan: Complex Adaptive Systems.

[33] Wiggins M, Bonell C, Sawtell M, Austerberry H, Burchett H, Allen E, Strange V (2009). Health outcomes of youth development programme in England: prospective matched comparison study. BMJ.

[34] Robinson M, Geue C, Lewsey J, Mackay D, McCartney G, Curnock E, Beeston C (2014). Evaluating the impact of the alcohol act on off-trade alcohol sales: a natural experiment in Scotland. Addiction.

[35] Plsek PE, Greenhalgh T (2001). Complexity science: the challenge of complexity in health care. BMJ.

[36] HM Treasury (2020). Magenta Book 2020 Supplementary Guide: Handling Complexity in Policy Evaluation.

[37] Centre for Complexity Across the Nexus (CECAN) (2019). Complexity Evaluation Framework Recognising Complexity & Key Considerations for Complexity-Appropriate Evaluation in the Department for Environment, Food and Rural Affairs (DEFRA).

[38] Zwarenstein M (2017). ‘Pragmatic’ and ‘explanatory’ attitudes to randomised trials. J R Soc Med.

[39] Loudon K, Treweek S, Sullivan F, Donnan P, Thorpe KE, Zwarenstein M (2015). The PRECIS-2 tool: designing trials that are fit for purpose. BMJ.

[40] Hawe P, Shiell A, Riley T (2004). Complex interventions: how ‘out of control’ can a randomised controlled trial be?. BMJ.

[41] Bonell C, Allen E, Warren E, McGowan J, Bevilacqua L, Jamal F (2018). Effects of the Learning Together intervention on bullying and aggression in English secondary schools (INCLUSIVE): a cluster randomised controlled trial. Lancet.

[42] Astbury B, Leeuw FL (2010). Unpacking black boxes: mechanisms and theory building in evaluation. Am J Eval.

[43] Borek AJ, Abraham C, Greaves CJ, Gillison F, Tarrant M, Morgan-Trimmer S (2019). Identifying change processes in group-based health behaviour-change interventions: development of the mechanisms of action in group-based interventions (MAGI) framework. Health Psychol Rev.

[44] Blamey A, Mackenzie M (2007). Theories of change and realistic evaluation: peas in a pod or apples and oranges?. Evaluation.

[45] Guba EG, Lincoln YS (1989). Fourth Generation Evaluation.

[46] Connell J, Kubisch A, Fulbright-Anderson K, Kubish AC, Connell JP (1998). New Approaches to Evaluating Community Initiatives, Vol. 2, Theory, Measurement, and Analysis.

[47] Connell JP, Kubisch AC, Schorr LB, Weiss CH (1995). New Approaches to Evaluating Community Initiatives.

[48] Pawson R, Tilley N (1997). Realistic Evaluation.

[49] Gallagher R, Appenzeller T (1999). Beyond reductionism. Science.

[50] Williams B (2015). Prosaic or profound? The adoption of systems ideas by impact evaluation. IDS Bulletin.

[51] Sutherland G, Stapleton JA, Russell MA, Jarvis MJ, Hajek P, Belcher M, Feyerabend C (1992). Randomised controlled trial of nasal nicotine spray in smoking cessation. Lancet.

[52] Nutbeam D, Macaskill P, Smith C, Simpson JM, Catford J (1993). Evaluation of two school smoking education programmes under normal classroom conditions. BMJ.

[53] Douglas FC, Gray DA, van Teijlingen ER (2010). Using a realist approach to evaluate smoking cessation interventions targeting pregnant women and young people. BMC Health Serv Res.

[54] Graham H (1976). Smoking in pregnancy: the attitudes of expectant mothers. Soc Sci Med.

[55] Haw SJ, Gruer L, Amos A, Currie C, Fischbacher C, Fong GT (2006). Legislation on smoking in enclosed public places in Scotland: how will we evaluate the impact?. J Public Health.

[56] Haw SJ, Gruer L (2007). Changes in exposure of adult non-smokers to secondhand smoke after implementation of smoke-free legislation in Scotland: national cross sectional survey. BMJ.

[57] Akhtar PC, Currie DB, Currie CE, Haw SJ (2007). Changes in child exposure to environmental tobacco smoke (CHETS) study after implementation of smoke-free legislation in Scotland: national cross sectional survey. BMJ.

[58] Pell JP, Haw S, Cobbe S, Newby DE, Pell AC, Fischbacher C (2008). Smoke-free legislation and hospitalizations for acute coronary syndrome. N Engl J Med.

[59] Mackay DF, Haw S, Pell JP (2011). Impact of Scottish smoke-free legislation on smoking quit attempts and prevalence. PLOS ONE.

[60] Eadie DR, MacAskill SG, Heim D, Hastings GB (2010). Responding to change: how did bar workers adapt to the smoke-free legislation in Scotland?. Int J Environ Health Res.

[61] Adda J, Berlinski S, Machin S (2007). Short-run economic effects of the Scottish smoking ban. Int J Epidemiol.

[62] Moore GF, Evans RE, Hawkins J, Littlecott H, Melendez-Torres GJ, Bonell C, Murphy S (2019). From complex social interventions to interventions in complex social systems: future directions and unresolved questions for intervention development and evaluation. Evaluation.

[63] Torjesen I (2015). Drug development: the journey of a medicine from lab to shelf. Pharma J.

[64] Pfadenhauer LM, Mozygemba K, Gerhardus A, Hofmann B, Booth A, Lysdahl KB (2015). Context and implementation: a concept analysis towards conceptual maturity. Z Evid Fortbild Qual Gesundhwes.

[65] Olds DL, Kitzman HJ, Cole RE, Hanks CA, Arcoleo KJ, Anson EA (2010). Enduring effects of prenatal and infancy home visiting by nurses on maternal life course and government spending: follow-up of a randomized trial among children at age 12 years. Arch Pediatr Adolesc Med.

[66] Eckenrode J, Campa M, Luckey DW, Henderson CR, Cole R, Kitzman H (2010). Long-term effects of prenatal and infancy nurse home visitation on the life course of youths: 19-year follow-up of a randomized trial. Arch Pediatr Adolesc Med.

[67] Mejdoubi J, van den Heijkant SC, van Leerdam FJ, Heymans MW, Crijnen A, Hirasing RA (2015). The effect of VoorZorg, the Dutch nurse-family partnership, on child maltreatment and development: a randomized controlled trial. PLOS ONE.

[68] Robling M, Bekkers MJ, Bell K, Butler CC, Cannings-John R, Channon S (2016). Effectiveness of a nurse-led intensive home-visitation programme for first-time teenage mothers (Building Blocks): a pragmatic randomised controlled trial. Lancet.

[69] Olds D (2016). Building evidence to improve maternal and child health. Lancet.

[70] Pfadenhauer LM, Gerhardus A, Mozygemba K, Lysdahl KB, Booth A, Hofmann B (2017). Making sense of complexity in context and implementation: the Context and Implementation of Complex Interventions (CICI) framework. Implement Sci.

[71] Greenhalgh T, Pawson R, Wong G, Westhorp G, Greenhalgh J, Manzano A (2017). What Realists Mean by Context; or Why Nothing Works Everywhere or for Everyone.

[72] Funnell SC, Rogers PJ (2011). Purposeful Program Theory Effective Use of Theories of Change and Logic Models.

[73] Pluye P, Potvin L, Denis JL, Pelletier J, Mannoni C (2005). Program sustainability begins with the first events. Eval Progr Plan.

[74] Lawless A, Baum F, Delany-Crowe T, MacDougall C, Williams C, McDermott D, Eyk HV (2018). Developing a framework for a program theory-based approach to evaluating policy processes and outcomes: health in all policies in South Australia. Int J Health Policy Manag.

[75] Craig P, Campbell M (2015). Evaluability Assessment: A Systematic Approach to Deciding Whether and How to Evaluate Programmes and Policies Working Paper.

[76] Bonell C, Jamal F, Melendez-Torres GJ, Cummins S (2015). ‘Dark logic’: theorising the harmful consequences of public health interventions. J Epidemiol Community Health.

[77] Shearn K, Allmark P, Piercy H, Hirst J (2017). Building realist program theory for large complex and messy interventions. Int J Qual Methods.

[78] Andersen DF, Vennix JAM, Richardson GP, Rouwette EAJA (2007). Group model building: problem structuring, policy simulation and decision support. J Operation Res Soc.

[79] Checkland P (2000). Soft systems methodology: a thirty year retrospective. Syst Res Behav Sci.

[80] Siokou C, Morgan R, Shiell A (2014). Group model building: a participatory approach to understanding and acting on systems. Public Health Res Pract.

[81] McGlashan J, Johnstone M, Creighton D, de la Haye K, Allender S (2016). Quantifying a systems map: network analysis of a childhood obesity causal loop diagram. PLOS ONE.

[82] Dalkin S, Lhussier M, Williams L, Burton CR, Rycroft-Malone J (2018). Exploring the use of Soft Systems Methodology with realist approaches: a novel way to map programme complexity and develop and refine programme theory. Evaluation.

[83] Davidoff F, Dixon-Woods M, Leviton L, Michie S (2015). Demystifying theory and its use in improvement. BMJ Qual Saf.

[84] Bartholomew Eldredge L, Markham C, Ruiter R, Fernandez M, Kok G, Parcel G (2016). Planning Health Promotion Programs: An Intervention Mapping Approach.

[85] Mills T, Lawton R, Sheard L (2019). Advancing complexity science in healthcare research: the logic of logic models. BMC Med Res Methodol.

[86] Moore GF, Evans RE (2017). What theory, for whom and in which context? Reflections on the application of theory in the development and evaluation of complex population health interventions. SSM Popul Health.

[87] Wong G, Greenhalgh T, Westhorp G, Buckingham J, Pawson R (2013). RAMESES publication standards: realist syntheses. J Adv Nurs.

[88] Blazeby JM, Byrne J, Welbourn R (2014). What is the most effective operation for adults with severe and complex obesity?. BMJ.

[89] Petticrew M, Knai C, Thomas J, Rehfuess EA, Noyes J, Gerhardus A (2019). Implications of a complexity perspective for systematic reviews and guideline development in health decision making. BMJ Global Health.

[90] Gottfredson DC, Cook TD, Gardner FE, Gorman-Smith D, Howe GW, Sandler IN, Zafft KM (2015). Standards of evidence for efficacy, effectiveness, and scale-up research in prevention science: next generation. Prev Sci.

[91] Lexchin J, Bero LA, Djulbegovic B, Clark O (2003). Pharmaceutical industry sponsorship and research outcome and quality: systematic review. BMJ.

[92] Moodie R, Stuckler D, Monteiro C, Sheron N, Neal B, Thamarangsi T (2013). Profits and pandemics: prevention of harmful effects of tobacco, alcohol, and ultra-processed food and drink industries. Lancet.

[93] Petticrew M, Katikireddi SV, Knai C, Cassidy R, Maani Hessari N, Thomas J, Weishaar H (2017). ‘Nothing can be done until everything is done’: the use of complexity arguments by food, beverage, alcohol and gambling industries. J Epidemiol Community Health.

[94] Greenhalgh T, Ozbilgin MF, Prainsack B, Shaw S (2019). Moral entrepreneurship, the powerknowledge nexus, and the Cochrane ‘crisis’. J Eval Clin Pract.

[95] INVOLVE (2015). What is Public Involvement in Research?.

[96] Crocker JC, Ricci-Cabello I, Parker A, Hirst JA, Chant A, Petit-Zeman S (2018). Impact of patient and public involvement on enrolment and retention in clinical trials: systematic review and meta-analysis. BMJ.

[97] Karazivan P, Dumez V, Flora L, Pomey MP, Del Grande C, Ghadiri DP (2015). The patient-as-partner approach in health care: a conceptual framework for a necessary transition. Acad Med.

[98] Wicks P, Richards T, Denegri S, Godlee F (2018). Patients’ roles and rights in research. BMJ.

[99] Meyrick J, Gray D (2018). Evidence-based patient/public voice: a patient and public involvement audit in the field of sexual health. BMJ Sex Reprod Health.

[100] Boivin A, Richards T, Forsythe L, Grégoire A, L’Espérance A, Abelson J, Carman KL (2018). Evaluating patient and public involvement in research. BMJ.

[101] Liabo K, Boddy K, Bortoli S, Irvine J, Boult H, Fredlund M (2020). Public involvement in health research: what does ‘good’ look like in practice?. Res Involv Engagem.

[102] INVOLVE (2015). Public Involvement in Research: Values and Principles Framework.

[103] National Institute for Health Research (NIHR) (2019). UK Standards for Public Involvement.

[104] National Institute for Health Research (NIHR) (2014). Patient and Public Involvement in Health and Social Care Research: A Handbook for Researchers.

[105] Greenhalgh T, Hinton L, Finlay T, Macfarlane A, Fahy N, Clyde B (2019). Frameworks for supporting patient and public involvement in research: systematic review and co-design pilot. Health Expect.

[106] Guise JM, O’Haire C, McPheeters M, Most C, Labrant L, Lee K (2013). A practice-based tool for engaging stakeholders in future research: a synthesis of current practices. J Clin Epidemiol.

[107] Staniszewska S, Brett J, Simera I, Seers K, Mockford C, Goodlad S (2017). GRIPP2 reporting checklists: tools to improve reporting of patient and public involvement in research. BMJ.

[108] Pollock A, Campbell P, Struthers C, Synnot A, Nunn J, Hill S (2019). Development of the ACTIVE framework to describe stakeholder involvement in systematic reviews. J Health Serv Res Policy.

[109] Medical Research Council (2017). Industry Charter.

[110] National Institute for Health Research (2011). Model Industry Collaborative Research Agreement (mICRA Guidance).

[111] Medical Research Council (2015). MRC Industry Collaboration Agreement (MICA).

[112] UK Prevention Research Partnership (UKPRP) (2018). Researchers Collaborating with Industry – Guidance on Policy and Procedures (Version 1.0).

[113] Intellectual Property Office (2016). University and Business Collaboration Agreements: Lambert Toolkit.

[114] Williams B, Hummelbrunner R (2010). Systems Concepts in Action: A Practitioner’s Toolkit.

[115] Checkland P, Scholes J (1999). Soft Systems Methodology in Action.

[116] Natural Sciences Methods and Tools for Co-Producing Knowledge.

[117] Network for Transdisciplinary Research Methods for Coproducing Knowledge.

[118] Claxton K, Cohen JT, Neumann PJ (2005). When is evidence sufficient?. Health Affairs.

[119] Kessler R, Glasgow RE (2011). A proposal to speed translation of healthcare research into practice: dramatic change is needed. Am J Prev Med.

[120] Briggs A, Sculpher M, Claxton K (2006). Decision Modelling for Health Economic Evaluation.

[121] Claxton K (2008). Exploring uncertainty in cost-effectiveness analysis. PharmacoEconomics.

[122] Silverman E, Gostoli U, Picascia S, Almagor J, McCann M, Shaw R (2020). Situating Agent-based Modelling in Population Health Research. airXiv.

[123] Petticrew M, McKee M, Lock K, Green J, Phillips G (2013). In search of social equipoise. BMJ.

[124] Fischer AJ, Threlfall A, Meah S, Cookson R, Rutter H, Kelly MP (2013). The appraisal of public health interventions: an overview. J Public Health.

[125] Rutter H, Wolpert M, Greenhalgh T (2020). Managing uncertainty in the covid-19 era. BMJ.

[126] Martin GP, Hanna E, McCartney M, Dingwall R (2020). Science, society, and policy in the face of uncertainty: reflections on the debate around face coverings for the public during COVID-19. Critical Public Health.

[127] Michie S, Yardley L, West R, Patrick K, Greaves F (2017). Developing and evaluating digital interventions to promote behavior change in health and health care: recommendations resulting from an international workshop. J Med Internet Res.

[128] Murray E, Hekler EB, Andersson G, Collins LM, Doherty A, Hollis C (2016). Evaluating digital health interventions: key questions and approaches. Am J Prev Med.

[129] Mohr DC, Cheung K, Schueller SM, Hendricks Brown C, Duan N (2013). Continuous evaluation of evolving behavioral intervention technologies. Am J Prev Med.

[130] Murray E, Treweek S, Pope C, MacFarlane A, Ballini L, Dowrick C (2010). Normalisation process theory: a framework for developing, evaluating and implementing complex interventions. BMC Med.

[131] Collins LM, Murphy SA, Bierman KL (2004). A conceptual framework for adaptive preventive interventions. Prev Sci.

[132] Drummond MF, Sculpher MJ, Claxton K, Stoddart GL, Torrance GW (2015). Methods for the Economic Evaluation of Health Care Programmes.

[133] Barnett ML, Dopp AR, Klein C, Ettner SL, Powell BJ, Saldana L (2020). Collaborating with health economists to advance implementation science: a qualitative study. Implement Sci Commun.

[134] National Institute for Health and Care Excellence (NICE) (2014). Developing NICE Guidelines: The Manual Chapter 7: Incorporating Economic Evaluation.

[135] Sanders GD, Neumann PJ, Basu A, Brock DW, Feeny D, Krahn M (2016). Recommendations for conduct, methodological practices, and reporting of cost-effectiveness analyses: second panel on cost-effectiveness in health and medicine. JAMA.

[136] Jabeen S (2018). Unintended outcomes evaluation approach: a plausible way to evaluate unintended outcomes of social development programmes. Eval Program Plan.

[137] Anderson R, Hardwick R, Pearson M, Byng R, Emme N, Greenhalgh J, Manzano A, Monaghan M, Dalkin S (2018). Doing Realist Research.

[138] Cookson R, Mirelman AJ, Griffin S, Asaria M, Dawkins B, Norheim OF (2017). Using costeffectiveness analysis to address health equity concerns. Value Health.

[139] Cookson R, Drummond M, Weatherly H (2009). Explicit incorporation of equity considerations into economic evaluation of public health interventions. Health Econ, Policy Law.

[140] Roberts M, Russell LB, Paltiel AD, Chambers M, McEwan P, Krahn M (2012). Conceptualizing a model: a report of the ISPOR-SMDM modeling good research practices Task Force-2. Med Decis.

[141] Tudor Edwards R, McIntosh E (2019). Applied Health Economics for Public Health Practice and Research.

[142] Deidda M, Geue C, Kreif N, Dundas R, McIntosh E (2019). A framework for conducting economic evaluations alongside natural experiments. Soc Sci Med.

[143] Ogilvie D, Adams J, Bauman A, Gregg EW, Panter J, Siegel KR (2020). Using natural experimental studies to guide public health action: turning the evidence-based medicine paradigm on its head. J Epidemiol Community Health.

[144] Escoffery C, Lebow-Skelley E, Haardoerfer R, Boing E, Udelson H, Wood R (2018). A systematic review of adaptations of evidence-based public health interventions globally. Implement Sci.

[145] Stirman SW, Miller CJ, Toder K, Calloway A (2013). Development of a framework and coding system for modifications and adaptations of evidence-based interventions. Implement Sci.

[146] Campbell R, Starkey F, Holliday J, Audrey S, Bloor M, Parry-Langdon N (2008). An informal school-based peer-led intervention for smoking prevention in adolescence (ASSIST): a cluster randomised trial. Lancet.

[147] Forsyth R, Purcell C, Barry S, Simpson S, Hunter R, McDaid L (2018). Peer-led intervention to prevent and reduce STI transmission and improve sexual health in secondary schools (STASH): protocol for a feasibility study. Pilot Feasibility Stud.

[148] White J, Hawkins J, Madden K, Grant A, Er V, Angel L (2017). Adapting the ASSIST model of informal peer-led intervention delivery to the Talk to FRANK drug prevention programme in UK secondary schools (ASSIST+ FRANK): intervention development, refinement and a pilot cluster randomised controlled trial. Public Health Res.

[149] Roberto CA, Kawachi I (2016). Behavioural Economics and Public Health.

[150] Levy DT, Yuan Z, Li Y, St Claire AW, Schillo BA (2019). The Minnesota SimSmoke tobacco control policy model of smokeless tobacco and cigarette use. Am J Prev Med.

[151] Li Y, Zhang D, Pagán JA (2016). Social norms and the consumption of fruits and vegetables across New York City neighborhoods. J Urban Health.

[152] Tappenden P (2012). Conceptual Modelling For Health Economic Model Development Health Economics and Decision Science Discussion Paper 1205.

[153] Squires H, Chilcott J, Akehurst R, Burr J, Kelly MP (2016). A framework for developing the structure of public health economic models. Value Health.

[154] Fenwick E, Palmer S, Claxton K, Sculpher M, Abrams K, Sutton A (2006). An iterative Bayesian approach to health technology assessment: application to a policy of preoperative optimization for patients undergoing major elective surgery. Med Decis Making.

[155] Johnson R, Woodcock J, de Nazelle A, de Sa T, Goel R, Tainio M (2019). A Guide to Value of Information Methods for Prioritising Research in Health Impact Modelling.

[156] O’Cathain A, Croot L, Sworn K, Duncan E, Rousseau N, Turner K (2019). Taxonomy of approaches to developing interventions to improve health: a systematic methods overview. Pilot Feasibility Stud.

[157] Duncan E, O’Cathain A, Rousseau N, Croot L, Sworn K, Turner KM (2020). Guidance for reporting intervention development studies in health research (GUIDED): an evidence-based consensus study. BMJ Open.

[158] Glasgow RE, Harden SM, Gaglio B, Rabin B, Smith ML, Porter GC (2019). RE-AIM planning and evaluation framework: adapting to new science and practice with a 20-year review. Front Public Health.

[159] Craig P, Katikireddi SV, Leyland A, Popham F (2017). Natural experiments: an overview of methods, approaches, and contributions to public health intervention research. Annu Rev Public Health.

[160] Michie S, Wood CE, Johnston M, Abraham C, Francis JJ, Hardeman W (2015). Behaviour change techniques: the development and evaluation of a taxonomic method for reporting and describing behaviour change interventions (a suite of five studies involving consensus methods, randomised controlled trials and analysis of qualitative data). Health Technol Assess.

[161] Hoffmann TC, Glasziou PP, Boutron I, Milne R, Perera R, Moher D (2014). Better reporting of interventions: template for intervention description and replication (TIDieR) checklist and guide. BMJ.

[162] Campbell M, Katikireddi SV, Hoffmann T, Armstrong R, Waters E, Craig P (2018). TIDieR-PHP: a reporting guideline for population health and policy interventions. BMJ.

[163] Eldridge SM, Chan CL, Campbell MJ, Bond CM, Hopewell S, Thabane L, Lancaster GA, PAFS consensus group (2016). CONSORT 2010 statement: extension to randomised pilot and feasibility trials. BMJ.

[164] Simpson SA, Matthews L, Pugmire J, McConnachie A, McIntosh E, Coulman E (2020). An app-, web- and social support-based weight loss intervention for adults with obesity: the HelpMeDoIt! feasibility RCT. Public Health Res.

[165] Moore GF, Audrey S, Barker M, Bond L, Bonell C, Hardeman W (2015). Process evaluation of complex interventions: Medical Research Council guidance. BMJ.

[166] Ogilvie D, Cummins S, Petticrew M, White M, Jones A, Wheeler K (2011). Assessing the evaluability of complex public health interventions: five questions for researchers, funders, and policymakers. Milbank Q.

[167] Hallingberg B, Turley R, Segrott J, Wight D, Craig P, Moore L (2018). Exploratory studies to decide whether and how to proceed with full-scale evaluations of public health interventions: a systematic review of guidance. Pilot Feasibility Stud.

[168] Moore L, Hallingberg B, Wight D, Turley R, Segrott J, Craig P (2018). Exploratory studies to inform full-scale evaluations of complex public health interventions: the need for guidance. J Epidemiol Community Health.

[169] McIntosh E, Clarke P, Frew E, Louviere JJ, Gray A, Briggs A (2010). Handbooks in Health Economic Evaluation.

[170] Husereau D, Drummond M, Petrou S, Carswell C, Moher D, Greenberg D (2013). Consolidated Health Economic Evaluation Reporting Standards (CHEERS) statement. BMJ.

[171] Ramsey S, Willke R, Briggs A, Brown R, Buxton M, Chawla A (2005). Good research practices for cost-effectiveness analysis alongside clinical trials: the ISPOR RCT-CEA Task Force report. Value Health.

[172] Deaton A, Cartwright N (2018). Understanding and misunderstanding randomized controlled trials. Soc Sci Med.

[173] UK Research and Innovation (UKRI) Pathways to Impact.

[174] Barnett HJM, Taylor DW, Haynes RB, Sackett DL, Peerless SJ, Ferguson GG (1991). Beneficial effect of carotid endarterectomy in symptomatic patients with high-grade carotid stenosis. N Engl J Med.

[175] Ashburn A, Pickering R, McIntosh E, Hulbert S, Rochester L, Roberts HC (2019). Exercise- and strategy-based physiotherapy-delivered intervention for preventing repeat falls in people with Parkinson’s: the PDSAFE RCT. Health Technol Assess.

[176] Mann CJ (2003). Observational research methods. Research design II: cohort, cross sectional, and case-control studies. Emerg Med J.

[177] Salisbury C, Man MS, Bower P, Guthrie B, Chaplin K, Gaunt DM (2018). Management of multimorbidity using a patient-centred care model: a pragmatic cluster-randomised trial of the 3D approach. Lancet.

[178] Haugen AS, Søfteland E, Almeland SK, Sevdalis N, Vonen B, Eide GE (2015). Effect of the World Health Organization checklist on patient outcomes: a stepped wedge cluster randomized controlled trial. Ann Surg.

[179] Kreif N, Grieve R, Hangartner D, Turner AJ, Nikolova S, Sutton M (2016). Examination of the synthetic control method for evaluating health policies with multiple treated units. Health Econ.

[180] Allender S, Millar L, Hovmand P, Bell C, Moodie M, Carter R (2016). Whole of Systems Trial of Prevention Strategies for childhood obesity: WHO STOPS childhood obesity. Int J Environ Res Public Health.

[181] Randell R, Greenhalgh J, Hindmarsh J, Dowding D, Jayne D, Pearman A (2014). Integration of robotic surgery into routine practice and impacts on communication, collaboration, and decision making: a realist process evaluation protocol. Implement Sci.

[182] Randell R, Honey S, Hindmarsh J, Alvarado N, Greenhalgh J, Pearman A (2017). A realist process evaluation of robot-assisted surgery: integration into routine practice and impacts on communication, collaboration and decision-making. Health Serv Deliv Res.

[183] Mukumbang FC, Marchal B, Van Belle S, van Wyk B (2018). ‘Patients are not following the [adherence] club rules anymore’: a realist case study of the antiretroviral treatment adherence club, South Africa. Qual Health Res.

[184] Popay J, Whitehead M, Carr-Hill R, Dibben C, Dixon P, Halliday E (2015). The impact on health inequalities of approaches to community engagement in the New Deal for Communities regeneration initiative: a mixed-methods evaluation. Public Health Res.

[185] Rosas S, Knight E (2019). Evaluating a complex health promotion intervention: case application of three systems methods. Crit Public Health.

[186] McGill E, Marks D, Sumpter C, Egan M (2016). Consequences of removing cheap, super-strength beer and cider: a qualitative study of a UK local alcohol availability intervention. BMJ Open.

[187] Sumpter C, McGill E, Dickie E, Champo E, Romeri E, Egan M (2016). Reducing the Strength: a mixed methods evaluation of alcohol retailers’ willingness to voluntarily reduce the availability of low cost, high strength beers and ciders in two UK local authorities. BMC Public Health.

[188] Blackman T, Wistow J, Byrne D (2011). A qualitative comparative analysis of factors associated with trends in narrowing health inequalities in England. Soc Sci Med.

[189] Singal AG, Higgins PD, Waljee AK (2014). A primer on effectiveness and efficacy trials. Clin Transl Gastroenterol.

[190] Mann C, Shaw ARG, Guthrie B, Wye L, Man MS, Chaplin K, Salisbury C (2019). Can implementation failure or intervention failure explain the result of the 3D multimorbidity trial in general practice: mixed-methods process evaluation. BMJ Open.

[191] Penney T, Adams J, Briggs A, Cummins S, Harrington R, Monsivais P (2018). Evaluation of the Impacts on Health of the Proposed UK Industry Levy on Sugar Sweetened Beverages: Developing a Systems Map and Data Platform, and Collection of Baseline and Early Impact Data.

[192] Williamson PR, Altman DG, Blazeby JM, Clarke M, Devane D, Gargon E, Tugwell P (2012). Developing core outcome sets for clinical trials: issues to consider. Trials.

[193] The COMET Initiative Core Outcome Measures in Effectiveness Trials.

[194] Cook JA, Julious SA, Sones W, Hampson LV, Hewitt C, Berlin JA (2018). DELTA2 guidance on choosing the target difference and undertaking and reporting the sample size calculation for a randomised controlled trial. BMJ.

[195] McKee M, Britton A, Black N, McPherson K, Sanderson C, Bain C (1999). Methods in health services research. Interpreting the evidence: choosing between randomised and non-randomised studies. BMJ.

[196] Creswell JW, Clark VLP (2017). Designing and Conducting Mixed Methods Research.

[197] O’Cathain A, Murphy E, Nicholl J (2010). Three techniques for integrating data in mixed methods studies. BMJ.

[198] Guetterman TC, Fetters MD, Creswell JW (2015). Integrating quantitative and qualitative results in health science mixed methods research through joint displays. Ann Fam Med.

[199] Richards DA, Bazeley P, Borglin G, Craig P, Emsley R, Frost J (2019). Integrating quantitative and qualitative data and findings when undertaking randomised controlled trials. BMJ Open.

[200] Craig P, Cooper C, Gunnell D, Haw S, Lawson K, Macintyre S (2012). Using natural experiments to evaluate population health interventions: new Medical Research Council guidance. J Epidemiol Community Health.

[201] Brand SL, Musgrove A, Jeffcoate WJ, Lincoln NB (2016). Evaluation of the effect of nurse education on patient-reported foot checks and foot care behaviour of people with diabetes receiving haemodialysis. Diabet Med.

[202] Hemming K, Haines TP, Chilton PJ, Girling AJ, Lilford RJ (2015). The stepped wedge cluster randomised trial: rationale, design, analysis, and reporting. BMJ.

[203] Thorlund K, Haggstrom J, Park JJ, Mills EJ (2018). Key design considerations for adaptive clinical trials: a primer for clinicians. BMJ.

[204] Kairalla JA, Coffey CS, Thomann MA, Muller KE (2012). Adaptive trial designs: a review of barriers and opportunities. Trials.

[205] Almirall D, Nahum-Shani I, Sherwood NE, Murphy SA (2014). Introduction to SMART designs for the development of adaptive interventions: with application to weight loss research. Transl Behav Med.

[206] Lei H, Nahum-Shani I, Lynch K, Oslin D, Murphy SA (2012). A ‘SMART’ design for building individualized treatment sequences. Annu Rev Clin Psychol.

[207] Bärnighausen T, Røttingen JA, Rockers P, Shemilt I, Tugwell P (2017). Quasi-experimental study designs series - paper 1: introduction: two historical lineages. J Clin Epidemiol.

[208] Basu S, Meghani A, Siddiqi A (2017). Evaluating the health impact of large-scale public policy changes: classical and novel approaches. Annu Rev Public Health.

[209] Chattopadhyay R, Duflo E (2004). Women as policy makers: evidence from a randomized policy experiment in India. Econometrica.

[210] Bernal JL, Cummins S, Gasparrini A (2017). Interrupted time series regression for the evaluation of public health interventions: a tutorial. Int J Epidemiol.

[211] Bor J, Moscoe E, Mutevedzi P, Newell ML, Bärnighausen T (2014). Regression discontinuity designs in epidemiology: causal inference without randomized trials. Epidemiology.

[212] Wing C, Simon K, Bello-Gomez RA (2018). Designing difference in difference studies: best practices for public health policy research. Annu Rev Public Health.

[213] Miller G, Pinto DM, Vera-Hernández M (2009). Risk Protection, Service Use, and Health Outcomes Under Colombia’s Health Insurance Program for the Poor No. 0898-2937.

[214] Hilton Boon M, Craig P, Thomson H, Campbell M, Moore L (2021). Regression discontinuity designs in health: a systematic review. Epidemiology.

[215] Bouttell J, Craig P, Lewsey J, Robinson M, Popham F (2018). Synthetic control methodology as a tool for evaluating population-level health interventions. J Epidemiol Community Health.

[216] Burges Watson D, Adams J, Azevedo LB, Haighton C (2016). Promoting physical activity with a school-based dance mat exergaming intervention: qualitative findings from a natural experiment. BMC Public Health.

[217] McCoy C (2009). Do drug courts work? For what, compared to what? Qualitative results from a natural experiment. Vict Offender.

[218] Bärnighausen T, Tugwell P, Røttingen JA, Shemilt I, Rockers P, Geldsetzer P (2017). Quasi-experimental study designs series – paper 4: uses and value. J Clin Epidemiol.

[219] Tugwell P, Knottnerus JA, McGowan J, Tricco A (2017). Big-5 quasi-experimental designs. J Clin Epidemiol.

[220] Sedgwick P (2014). What is an ‘n-of-1’ trial?. BMJ.

[221] McDonald S, Quinn F, Vieira R, O’Brien N, White M, Johnston DW, Sniehotta FF (2017). The state of the art and future opportunities for using longitudinal n-of-1 methods in health behaviour research: a systematic literature overview. Health Psychol Rev.

[222] Gabler NB, Duan N, Vohra S, Kravitz RL (2011). N-of-1 trials in the medical literature: a systematic review. Med Care.

[223] Starkey F, Audrey S, Holliday J, Moore L, Campbell R (2009). Identifying influential young people to undertake effective peer-led health promotion: the example of A Stop Smoking In Schools Trial (ASSIST). Health Educ Res.

[224] White M, Cummins S, Rayner M, Smith R, Rutter H, Adams J (2018). Evaluation of the Health Impacts of the UK Treasury Soft Drinks Industry Levy (SDIL) Project Protocol.

[225] Bruch E, Atwell J (2013). Agent-based models in empirical social research. Sociol Methods Res.

[226] Tracy M, Cerdá M, Keyes KM (2018). Agent-based modeling in public health: current applications and future directions. Annu Rev Public Health.

[227] Kypridemos C, Collins B, McHale P, Bromley H, Parvulescu P, Capewell S, O’Flaherty M (2018). Future cost-effectiveness and equity of the NHS health check cardiovascular disease prevention programme: microsimulation modelling using data from Liverpool, UK. PLOS Med.

[228] Kypridemos C, Guzman-Castillo M, Hyseni L, Hickey GL, Bandosz P, Buchan I (2017). Estimated reductions in cardiovascular and gastric cancer disease burden through salt policies in England: an IMPACTNCD microsimulation study. BMJ Open.

[229] Allen K, Pearson-Stuttard J, Hooton W, Diggle P, Capewell S, O’Flaherty M (2015). Potential of trans fats policies to reduce socioeconomic inequalities in mortality from coronary heart disease in England: cost effectiveness modelling study. BMJ.

[230] Smith RD, Keogh-Brown MR, Barnett T, Tait J (2009). The economy-wide impact of pandemic influenza on the UK: a computable general equilibrium modelling experiment. BMJ.

[231] Holmes J, Meng Y, Meier PS, Brennan A, Angus C, Campbell-Burton A (2014). Effects of minimum unit pricing for alcohol on different income and socioeconomic groups: a modelling study. Lancet.

[232] Blackman T, Wistow J, Byrne D (2013). Using qualitative comparative analysis to understand complex policy problems. Evaluation.

[233] Byrne D (2016). Qualitative Comparative Analysis: A Pragmatic Method for Evaluating Intervention A CECAN Evaluation and Policy Practice Note for Policy Analysts and Evaluators.

[234] Rihoux B, Ragin CC (2008). Configurational Comparative Methods: Qualitative Comparative Analysis (QCA) and Related Techniques.

[235] Bonell C, Fletcher A, Morton M, Lorenc T, Moore L (2012). Realist randomised controlled trials: a new approach to evaluating complex public health interventions. Soc Sci Med.

[236] McGill E, Marks D, Er V, Penney T, Petticrew M, Egan M (2020). Qualitative process evaluation from a complex systems perspective: a systematic review and framework for public health evaluators. PLOS Med.

[237] Charles J, Jones A, Lloyd-Williams H, Edwards R, McIntosh E (2019). An Introduction to the Principles of Health Economics and Economic Evaluation Relevant to the Specific Challenges of Public Health and its Aims of Improving Population Health.

[238] Marsh K, IJzerman M, Thokala P, Baltussen R, Boysen M, Kaló Z (2016). Multiple criteria decision analysis for health care decision making – emerging good practices: report 2 of the ISPOR MCDA Emerging Good Practices Task Force. Value Health.

[239] Philips Z, Ginnelly L, Sculpher M, Claxton K, Golder S, Riemsma R (2004). Review of guidelines for good practice in decision-analytic modelling in health technology assessment. Health Technol Assess.

[240] Karnon J, Stahl J, Brennan A, Caro JJ, Mar J, Möller J, ISPOR-SMDM Modeling Good Research Practices Task Force (2012). Modeling using discrete event simulation: a report of the ISPOR-SMDM Modeling Good Research Practices Task Force - 4. Value Health.

[241] Mathes T, Antoine SL, Prengel P, Bühn S, Polus S, Pieper D (2017). Health technology assessment of public health interventions: a synthesis of methodological guidance. Int J Technol Assess Health Care.

[242] Caro JJ, Briggs AH, Siebert U, Kuntz KM, ISPOR-SMDM Modeling Good Research Practices Task Force (2012). Modeling good research practices – overview: a report of the ISPOR-SMDM Modeling Good Research Practices Task Force-1. Med Decis Making.

[243] Squires H, Boyd KA, McIntosh E (2019). An Introduction to the Principles of Health Economics and Economic Evaluation Relevant to the Specific Challenges of Public Health and its Aims of Improving Population Health.

[244] Montgomery P, Grant S, Mayo-Wilson E, Macdonald G, Michie S, Hopewell S, Moher D, CONSORT-SPI Group (2018). Reporting randomised trials of social and psychological interventions: the CONSORT-SPI 2018 extension. Trials.

[245] Campbell MK, Piaggio G, Elbourne DR, Altman DG, CONSORT Group (2012). Consort 2010 statement: extension to cluster randomised trials. BMJ.

[246] Zwarenstein M, Treweek S, Gagnier JJ, Altman DG, Tunis S, Haynes B (2008). Improving the reporting of pragmatic trials: an extension of the CONSORT statement. BMJ.

[247] Vohra S, Shamseer L, Sampson M, Bukutu C, Schmid CH, Tate R (2015). CONSORT extension for reporting N-of-1 trials (CENT) 2015 statement. BMJ.

[248] Hemming K, Taljaard M, McKenzie JE, Hooper R, Copas A, Thompson JA (2018). Reporting of stepped wedge cluster randomised trials: extension of the CONSORT 2010 statement with explanation and elaboration. BMJ.

[249] Boutron I, Altman DG, Moher D, Schulz KF, Ravaud P, CONSORT NPT Group (2017). CONSORT statement for randomized trials of nonpharmacologic treatments: a 2017 update and a CONSORT extension for nonpharmacologic trial abstracts. Ann Intern Med.

[250] Calvert M, Blazeby J, Altman DG, Revicki DA, Moher D, Brundage MD, CONSORT PRO Group (2013). Reporting of patient-reported outcomes in randomized trials: the CONSORT PRO extension. JAMA.

[251] Dimairo M, Pallmann P, Wason J, Todd S, Jaki T, Julious SA (2020). The Adaptive designs CONSORT Extension (ACE) statement: a checklist with explanation and elaboration guideline for reporting randomised trials that use an adaptive design. BMJ.

[252] Calvert M, Kyte D, Mercieca-Bebber R, Slade A, Chan AW, King MT (2018). Guidelines for inclusion of patient-reported outcomes in clinical trial protocols: the SPIRIT-PRO extension. JAMA.

[253] Gamble C, Krishan A, Stocken D, Lewis S, Juszczak E, Doré C (2017). Guidelines for the content of statistical analysis plans in clinical trials. JAMA.

[254] Grant A, Treweek S, Dreischulte T, Foy R, Guthrie B (2013). Process evaluations for cluster-randomised trials of complex interventions: a proposed framework for design and reporting. Trials.

[255] Wong G, Westhorp G, Manzano A, Greenhalgh J, Jagosh J, Greenhalgh T (2016). RAMESES II reporting standards for realist evaluations. BMC Med.

[256] Chan AW, Tetzlaff JM, Altman DG, Laupacis A, Gøtzsche PC, Krleža-Jerić K (2013). SPIRIT 2013 statement: defining standard protocol items for clinical trials. Ann Intern Med.

[257] O’Brien BC, Harris IB, Beckman TJ, Reed DA, Cook DA (2014). Standards for reporting qualitative research: a synthesis of recommendations. Acad Med.

[258] von Elm E, Altman DG, Egger M, Pocock SJ, Gøtzsche PC, Vandenbroucke JP, STROBE Initiative (2007). The Strengthening the Reporting of Observational Studies in Epidemiology (STROBE) statement: guidelines for reporting observational studies. Ann Intern Med.

[259] Thorpe KE, Zwarenstein M, Oxman AD, Treweek S, Furberg CD, Altman DG (2009). A pragmatic-explanatory continuum indicator summary (PRECIS): a tool to help trial designers. J Clin Epidemiol.

[260] BMC (2018). Sample Size Calculations and Specifying the Target Difference for Randomized Trials An Article Collection Published in Trials.

[261] Remme M, Martinez-Alvarez M, Vassall A (2017). Cost-effectiveness thresholds in global health: taking a multisectoral perspective. Value Health.

[262] Terris-Prestholt F, Neke N, Grund JM, Plotkin M, Kuringe E, Osaki H (2019). Using discrete choice experiments to inform the design of complex interventions. Trials.

[263] University of Birmingham ICECAP Capability Measures.

[264] Al-Janabi H, Flynn TN, Coast J (2012). Development of a self-report measure of capability wellbeing for adults: the ICECAP-A. Qual Life Res.

[265] Deidda M, Boyd KA, Minnis H, Donaldson J, Brown K, Boyer NRS, McIntosh E, BeST study team (2018). Protocol for the economic evaluation of a complex intervention to improve the mental health of maltreated infants and children in foster care in the UK (the BeST? services trial). BMJ Open.

[266] Kreif N, Grieve R, Sadique MZ (2013). Statistical methods for cost-effectiveness analyses that use observational data: a critical appraisal tool and review of current practice. Health Econ.

[267] World Health Organization (2016). Scaling Up Projects and Initiatives for Better Health: From Concepts to Practice.

[268] Grimshaw JM, Thomas RE, MacLennan G, Fraser C, Ramsay CR, Vale L (2004). Effectiveness and efficiency of guideline dissemination and implementation strategies. Health Technol Assess.

[269] Damschroder LJ, Aron DC, Keith RE, Kirsh SR, Alexander JA, Lowery JC (2009). Fostering implementation of health services research findings into practice: a consolidated framework for advancing implementation science. Implement Sci.

[270] Eccles MP, Mittman BS (2006). Welcome to implementation science. Implement Sci.

[271] Theobald S, Brandes N, Gyapong M, El-Saharty S, Proctor E, Diaz T (2018). Implementation research: new imperatives and opportunities in global health. Lancet.

[272] Huis A, Holleman G, van Achterberg T, Grol R, Schoonhoven L, Hulscher M (2013). Explaining the effects of two different strategies for promoting hand hygiene in hospital nurses: a process evaluation alongside a cluster randomised controlled trial. Implement Sci.

[273] Pinnock H, Barwick M, Carpenter CR, Eldridge S, Grandes G, Griffiths CJ (2017). Standards for Reporting Implementation studies (StaRI) statement. BMJ.

[274] Aveyard P, Lewis A, Tearne S, Hood K, Christian-Brown A, Adab P (2016). Screening and brief intervention for obesity in primary care: a parallel, two-arm, randomised trial. Lancet.

[275] Diepeveen S, Ling T, Suhrcke M, Roland M, Marteau TM (2013). Public acceptability of government intervention to change health-related behaviours: a systematic review and narrative synthesis. BMC Public Health.

[276] Presseau J, Sniehotta FF, Francis JJ, Campbell NC (2009). Multiple goals and time constraints: perceived impact on physicians’ performance of evidence-based behaviours. Implement Sci.

[277] Petticrew M, Whitehead M, Macintyre SJ, Graham H, Egan M (2004). Evidence for public health policy on inequalities: 1: the reality according to policymakers. J Epidemiol Community Health.

[278] May C (2006). Mobilising modern facts: health technology assessment and the politics of evidence. Sociol Health Illn.

[279] Pick W (2008). Lack of evidence hampers human-resources policy making. Lancet.

[280] Kelly MP, Speller V, Meyrick J (2004). Getting Evidence into Practice in Public Health.

[281] Michie S, Johnston M (2004). Changing clinical behaviour by making guidelines specific. BMJ.

[282] Curran GM, Bauer M, Mittman B, Pyne JM, Stetler C (2012). Effectiveness-implementation hybrid designs: combining elements of clinical effectiveness and implementation research to enhance public health impact. Med Care.

[283] Landes SJ, McBain SA, Curran GM (2019). An introduction to effectiveness-implementation hybrid designs. Psychiatry Res.

[284] Green BB, Coronado GD, Schwartz M, Coury J, Baldwin LM (2019). Using a continuum of hybrid effectiveness-implementation studies to put research-tested colorectal screening interventions into practice. Implement Sci.

[285] Villeval M, Bidault E, Shoveller J, Alias F, Basson JC, Frasse C (2016). Enabling the transferability of complex interventions: exploring the combination of an intervention’s key functions and implementation. Int J Public Health.

[286] Imison C, Curry N, Holder H, Castle-Clarke S, Nimmons D, Appleby J (2017). Shifting the Balance of Care: Great Expectations.

[287] Bellg AJ, Borrelli B, Resnick B, Hecht J, Minicucci DS, Ory M (2004). Enhancing treatment fidelity in health behavior change studies: best practices and recommendations from the NIH Behavior Change Consortium. Health Psychol.

[288] Fagg J, Cole TJ, Cummins S, Goldstein H, Morris S, Radley D (2015). After the RCT: who comes to a family-based intervention for childhood overweight or obesity when it is implemented at scale in the community?. J Epidemiol Community Health.

[289] Hull L, Goulding L, Khadjesari Z, Davis R, Healey A, Bakolis I, Sevdalis N (2019). Designing high-quality implementation research: development, application, feasibility and preliminary evaluation of the implementation science research development (ImpRes) tool and guide. Implement Sci.

[290] Bauer MS, Damschroder L, Hagedorn H, Smith J, Kilbourne AM (2015). An introduction to implementation science for the non-specialist. BMC Psychol.

[291] Nilsen P (2015). Making sense of implementation theories, models and frameworks. Implement Sci.

[292] Greenhalgh T, Robert G, Macfarlane F, Bate P, Kyriakidou O (2004). Diffusion of innovations in service organizations: systematic review and recommendations. Milbank Q.

[293] Meyers DC, Durlak JA, Wandersman A (2012). The quality implementation framework: a synthesis of critical steps in the implementation process. Am J Community Psychol.

[294] Gray CM, Hunt K, Mutrie N, Anderson AS, Leishman J, Dalgarno L, Wyke S (2013). Football Fans in Training: the development and optimization of an intervention delivered through professional sports clubs to help men lose weight, become more active and adopt healthier eating habits. BMC Public Health.

[295] Gray CM, Hunt K, Mutrie N, Anderson AS, Treweek S, Wyke S (2013). Weight management for overweight and obese men delivered through professional football clubs: a pilot randomized trial. Int J Behav Nutr Phys Act.

[296] Hunt K, Gray CM, Maclean A, Smillie S, Bunn C, Wyke S (2014). Do weight management programmes delivered at professional football clubs attract and engage high risk men? A mixed-methods study. BMC Public Health.

[297] Hunt K, Wyke S, Gray CM, Anderson AS, Brady A, Bunn C (2014). A gender-sensitised weight loss and healthy living programme for overweight and obese men delivered by Scottish Premier League football clubs (FFIT): a pragmatic randomised controlled trial. Lancet.

[298] Hunt K, Wyke S, Bunn C, Donnachie C, Reid N, Gray CM (2020). Scale-up and scale-out of a gender-sensitized weight management and healthy living program delivered to overweight men via professional sports clubs: the wider implementation of Football Fans in Training (FFIT). Int J Environ Res Public Health.

[299] Hoddinott P, Britten J, Pill R (2010). Why do interventions work in some places and not others: a breastfeeding support group trial. Soc Sci Med.

[300] Hoddinott P, Britten J, Prescott GJ, Tappin D, Ludbrook A, Godden DJ (2009). Effectiveness of policy to provide breastfeeding groups (BIG) for pregnant and breastfeeding mothers in primary care: cluster randomised controlled trial. BMJ.

[301] Stephens TJ, Peden CJ, Pearse RM, Shaw SE, Abbott TEF, Jones EL (2018). Improving care at scale: process evaluation of a multi-component quality improvement intervention to reduce mortality after emergency abdominal surgery (EPOCH trial). Implement Sci.

[302] Peden CJ, Stephens T, Martin G, Kahan BC, Thomson A, Rivett K (2019). Effectiveness of a national quality improvement programme to improve survival after emergency abdominal surgery (EPOCH): a stepped-wedge cluster-randomised trial. Lancet.

[303] Maini R, Mounier-Jack S, Borghi J (2018). How to and how not to develop a theory of change to evaluate a complex intervention: reflections on an experience in the Democratic Republic of Congo. BMJ Global Health.

[304] Spero JC, McQuide PA, Matte R (2011). Tracking and monitoring the health workforce: a new human resources information system (HRIS) in Uganda. Hum Resour Health.

[305] Franco LM, Bennett S, Kanfer R (2002). Health sector reform and public sector health worker motivation: a conceptual framework. Soc Sci Med.

[306] World Health Organization (2006). Working Together for Health - The World Health Report 2006.

[307] Cook PA, Hargreaves SC, Burns EJ, de Vocht F, Parrott S, Coffey M (2018). Communities in charge of alcohol (CICA): a protocol for a stepped-wedge randomised control trial of an alcohol health champions programme. BMC Public Health.

[308] Ebenso B, Manzano A, Uzochukwu B, Etiaba E, Huss R, Ensor T (2019). Dealing with context in logic model development: reflections from a realist evaluation of a community health worker programme in Nigeria. Eval Program Plann.

[309] Jones S, Riste L, Barrowclough C, Bartlett P, Clements C, Davies L (2018). Reducing relapse and suicide in bipolar disorder: practical clinical approaches to identifying risk, reducing harm and engaging service users in planning and delivery of care – the PARADES (Psychoeducation, Anxiety, Relapse, Advance Directive Evaluation and Suicidality) programme. Programme Grants Appl Res.

[310] Great Britain (2005). Mental Capacity Act 2005.

[311] Yardley L, Ainsworth B, Arden-Close E, Muller I (2015). The person-based approach to enhancing the acceptability and feasibility of interventions. Pilot Feasibility Stud.

[312] Minnis H, Bryce G, Phin L, Wilson P (2010). The ‘Spirit of New Orleans’: translating a model of intervention with maltreated children and their families for the Glasgow context. Clin Child Psychol Psychiatry.

[313] Boyd KA, Balogun MO, Minnis H (2016). Development of a radical foster care intervention in Glasgow, Scotland. Health Promot Int.

[314] Pritchett R, Fitzpatrick B, Watson N, Cotmore R, Wilson P, Bryce G (2013). A feasibility randomised controlled trial of the New Orleans intervention for infant mental health: a study protocol. Sci World J.

[315] Turner-Halliday F, Kainth G, Young-Southward G, Cotmore R, Watson N, McMahon L, Minnis H (2017). Clout or doubt? Perspectives on an infant mental health service for young children placed in foster care due to abuse and neglect. Child Abuse Negl.

[316] Turner-Halliday F, Watson N, Minnis H (2016). Process Evaluation of the New Orleans Intervention Model for Infant Mental Health in Glasgow.

[317] Minnis H, Wilson P, Boyd K, Messow C, Bryce G, Donaldson J (2016). The BeST? Services Trial: Evaluation of the New Orleans Intervention Model for Infants Mental Health in Glasgow.

[318] Minnis H, Boyd KA, Fitzpatrick B (2016). Protocol 15PRT/6090: The Best Services Trial (BeST?): Effectiveness and Cost-effectiveness of the New Orleans Intervention Model for Infant Mental Health - NCT02653716.

[319] Signal LN, Walton MD, Ni Mhurchu C, Maddison R, Bowers SG, Carter KN (2013). Tackling ‘wicked’ health promotion problems: a New Zealand case study. Health Promot Int.

[320] Blackman T (2006). Placing Health: Neighbourhood Renewal, Health Improvement and Complexity.

[321] Byrne D (2001). What is complexity science? Thinking as a realist about measurement and cities and arguing for natural history. Emergence.

[322] Byrne DS (1998). Complexity Theory and the Social Sciences: An Introduction.

[323] Coffin PO, Santos GM, Behar E, Hern J, Walker J, Matheson T (2019). Randomized feasibility trial of directly observed versus unobserved hepatitis C treatment with ledipasvir-sofosbuvir among people who inject drugs. PLOS ONE.

[324] Honeycutt S, Hermstad A, Carvalho ML, Arriola KRJ, Ballard D, Escoffery C (2017). Practice to evidence: using evaluability assessment to generate practice-based evidence in rural South Georgia. Health Educ Behav.

[325] Mann C, Shaw A, Guthrie B, Wye L, Man MS, Hollinghurst S (2016). Protocol for a process evaluation of a cluster randomised controlled trial to improve management of multimorbidity in general practice: the 3D study. BMJ Open.

[326] Man MS, Chaplin K, Mann C, Bower P, Brookes S, Fitzpatrick B (2016). Improving the management of multimorbidity in general practice: protocol of a cluster randomised controlled trial (the 3D study). BMJ Open.

[327] Thorn J, Man MS, Chaplin K, Bower P, Brookes S, Gaunt D (2020). Cost-effectiveness of a patient-centred approach to managing multimorbidity in primary care: a pragmatic cluster randomised controlled trial. BMJ Open.

[328] Sutton M, Nikolova S, Boaden R, Lester H, McDonald R, Roland M (2012). Reduced mortality with hospital pay for performance in England. N Engl J Med.

[329] Abadie A, Diamond A, Hainmueller J (2010). Synthetic control methods for comparative case studies: estimating the effect of California’s tobacco control program. J Am Stat Assoc.

[330] Allender S, Owen B, Kuhlberg J, Lowe J, Nagorcka-Smith P, Whelan J, Bell C (2015). A community based systems diagram of obesity causes. PLOS ONE.

[331] Rose J, Riolo R, Hovmand P, Cherng S, Ferrer R, Katerndahl DA, Sturmberg JP, Martin CM (2013). Handbook of Systems and Complexity in Health.

[332] Jayne D, Pigazzi A, Marshall H, Croft J, Corrigan N, Copeland J (2017). Effect of robotic-assisted vs conventional laparoscopic surgery on risk of conversion to open laparotomy among patients undergoing resection for rectal cancer: the ROLARR randomized clinical trial. JAMA.

[333] Collinson FJ, Jayne DG, Pigazzi A, Tsang C, Barrie JM, Edlin R (2012). An international, multicentre, prospective, randomised, controlled, unblinded, parallel-group trial of robotic-assisted versus standard laparoscopic surgery for the curative treatment of rectal cancer. Int J Colorectal Dis.

[334] Mukumbang FC, Van Belle S, Marchal B, Van Wyk B (2016). Realist evaluation of the antiretroviral treatment adherence club programme in selected primary healthcare facilities in the metropolitan area of Western Cape Province, South Africa: a study protocol. BMJ Open.

[335] Bloor M, Frankland J, Langdon NP, Robinson M, Allerston S, Catherine A (1999). A controlled evaluation of an intensive, peer-led, schools-based, anti-smoking programme. Health Educ J.

[336] Holliday J, Audrey S, Moore L, Parry-Langdon N, Campbell R (2009). High fidelity? How should we consider variations in the delivery of school-based health promotion interventions?. Health Educ J.

[337] Hollingworth W, Cohen D, Hawkins J, Hughes RA, Moore LA, Holliday JC (2012). Reducing smoking in adolescents: cost-effectiveness results from the cluster randomized ASSIST (A Stop Smoking In Schools Trial). Nicotine Tob Res.

[338] Butler M, Epstein RA, Totten A, Whitlock EP, Ansari MT, Damschroder LJ (2017). AHRQ series on complex intervention systematic reviews – paper 3: adapting frameworks to develop protocols. J Clin Epidemiol.

[339] Noyes J, Gough D, Lewin S, Mayhew A, Michie S, Pantoja T (2013). A research and development agenda for systematic reviews that ask complex questions about complex interventions. J Clin Epidemiol.

[340] Shepperd S, Lewin S, Straus S, Clarke M, Eccles MP, Fitzpatrick R (2009). Can we systematically review studies that evaluate complex interventions?. PLOS Med.

[341] Kelly MP, Noyes J, Kane RL, Chang C, Uhl S, Robinson KA (2017). AHRQ series on complex intervention systematic reviews – paper 2: defining complexity, formulating scope, and questions. J Clin Epidemiol.

[342] Squires JE, Valentine JC, Grimshaw JM (2013). Systematic reviews of complex interventions: framing the review question. J Clin Epidemiol.

[343] Pawson R, Greenhalgh T, Harvey G, Walshe K (2005). Realist review – a new method of systematic review designed for complex policy interventions. J Health Serv Res Policy.

[344] Higgins JPT, Thomas J, Chandler J, Cumpston M, Li T, Page MJ (2020). Cochrane Handbook for Systematic Reviews of Interventions Version 6.1.

[345] Norris SL, Rehfuess EA, Smith H, Tunçalp Ö, Grimshaw JM, Ford NP (2019). Complex health interventions in complex systems: improving the process and methods for evidence-informed health decisions. BMJ Glob Health.

[346] Booth A, Noyes J, Flemming K, Moore G, Tunçalp Ö, Shakibazadeh E (2019). Formulating questions to explore complex interventions within qualitative evidence synthesis. BMJ Glob Health.

[347] Flemming K, Booth A, Hannes K, Cargo M, Noyes J (2018). Cochrane Qualitative and Implementation Methods Group guidance series – paper 6: reporting guidelines for qualitative, implementation, and process evaluation evidence syntheses. J Clin Epidemiol.

[348] Noyes J, Booth A, Moore G, Flemming K, Tunçalp Ö, Shakibazadeh E (2019). Synthesising quantitative and qualitative evidence to inform guidelines on complex interventions: clarifying the purposes, designs and outlining some methods. BMJ Glob Health.

[349] Booth A, Moore G, Flemming K, Garside R, Rollins N, Tunçalp Ö (2019). Taking account of context in systematic reviews and guidelines considering a complexity perspective. BMJ Glob Health.

[350] Rehfuess EA, Stratil JM, Scheel IB, Portela A, Norris SL, Baltussen R (2019). The WHO-INTEGRATE evidence to decision framework version 1.0: integrating WHO norms and values and a complexity perspective. BMJ Glob Health.

[351] Tugwell P, Knottnerus JA, Idzerda L (2013). Complex interventions – how should systematic reviews of their impact differ from reviews of simple or complicated interventions?. J Clin Epidemiol.

[352] Guise JM, Chang C, Butler M, Viswanathan M, Tugwell P (2017). AHRQ series on complex intervention systematic reviews – paper 1: an introduction to a series of articles that provide guidance and tools for reviews of complex interventions. J Clin Epidemiol.

[353] Lewin S, Hendry M, Chandler J, Oxman AD, Michie S, Shepperd S (2017). Assessing the complexity of interventions within systematic reviews: development, content and use of a new tool (iCAT_SR). BMC Med Res Methodol.

[354] Moher D, Liberati A, Tetzlaff J, Altman DG, PRISMA Group (2009). Preferred reporting items for systematic reviews and meta-analyses: the PRISMA statement. Ann Intern Med.

[355] Guise JM, Butler M, Chang C, Viswanathan M, Pigott T, Tugwell P, Complex Interventions Workgroup (2017). AHRQ series on complex intervention systematic reviews – paper 7: PRISMA-CI elaboration and explanation. J Clin Epidemiol.

[356] Guise JM, Butler ME, Chang C, Viswanathan M, Pigott T, Tugwell P, Complex Interventions Workgroup (2017). AHRQ series on complex intervention systematic reviews – paper 6: PRISMA-CI extension statement and checklist. J Clin Epidemiol.

[357] France EF, Cunningham M, Ring N, Uny I, Duncan EAS, Jepson RG (2019). Improving reporting of meta-ethnography: the eMERGe reporting guidance. BMC Med Res Methodol.

[358] Montgomery P, Movsisyan A, Grant SP, Macdonald G, Rehfuess EA (2019). Considerations of complexity in rating certainty of evidence in systematic reviews: a primer on using the GRADE approach in global health. BMJ Glob Health.
